# Organocatalytic diastereo- and enantioselective conjugate addition of pyrazol-3-ones to 3-trifluoroethylidene oxindoles with a newly developed squaramide catalyst†

**DOI:** 10.1039/d2ra05088a

**Published:** 2022-09-21

**Authors:** Zhao Han, Jiaping Jin, Alemayehu Gashaw Woldegiorgis, Xufeng Lin

**Affiliations:** Department of Chemistry, Zhejiang University Hangzhou 310027 P. R. China lxfok@zju.edu.cn

## Abstract

An efficient organocatalytic conjugated addition reaction of pyrazol-3-ones with 3-trifluoroethylidene oxindoles has been developed for the synthesis of enantioenriched triflouromethylated indolin-2-ones bearing adjacent tertiary chiral centers in good yields and good to excellent diastereo- and enantioselectivities. The use of a newly developed chiral spirobiindane-derived squaramide catalyst is essential in achieving high diastereo- and enantioselectivities.

## Abstract 2

Organocatalytic diastereo- and enantioselective conjugate addition of pyrazol-3-ones to 3-trifluoroethylidene oxindoles with a newly developed squaramide catalyst has been developed.

## Graphical abstract

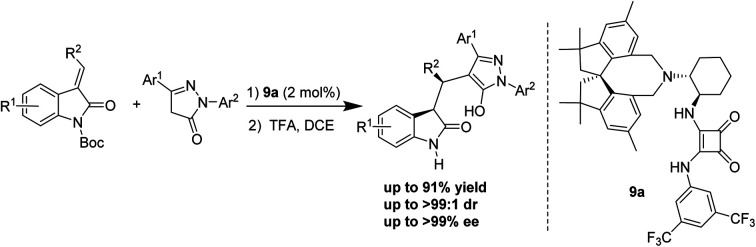

The development of efficient chiral catalysts for asymmetric organocatalytic transformations has become one of the greatest challenges in chemical synthesis.^1^ Since the pioneering work of Rawal and coworkers^2^ in 2008, chiral squaramide catalysis has enabled many enantioselective organic reactions.^3^ In particular, chiral bifunctional squaramide catalysts with cinchonine and BINOL backbones represent a great achievement in asymmetric synthesis, as they provide excellent stereoselectivity in many organic reactions.^4^ Notwithstanding this remarkable progress, there are still many synthetically useful transformations that remain unattainable in an asymmetric manner.^5^ The chiral framework of the catalyst plays a crucial role in its performance, thus the development of new and efficient bifunctional squaramide catalysts with different backbones is still highly valuable and desirable for asymmetric transformations. Based on our interest in the discovery of chiral spirobiindane-derived organocatalysts for asymmetric synthesis,^6^ a new chiral bifunctional squaramide catalyst based on spirobiindane was synthesized and applied in the asymmetric conjugate addition of pyrazol-3-ones with 3-trifluoroethylidene oxindoles.

Pyrazolones have a wide range of applications in dyes, pharmaceutical chemistry^7^ and possess enormous biological activities.^8^ Thus, some efficient asymmetric organocatalytic conjugate addition reactions of pyrazol-3-ones with various electrophiles have been reported.^9^ However, other electrophiles, especially those that could lead to biologically interesting scaffolds, are highly needed. Therefore, we selected 3-trifluoroethylidene oxindoles as electrophile to react with pyrazol-3-ones, providing triflouromethylated indolin-2-ones with adjacent tertiary chiral centers, which were found in biologically active natural products and pharmaceutically active compounds.^10,11^

Furthermore, the asymmetric synthesis of 3-substituted oxindole scaffolds with fluorine atoms has attracted considerable attention^12^ because fluorine-containing organic molecules serve as versatile and valuable motifs in the agrochemical industry, medicinalchemistry, and material sciences due to their lipophilicity, easy solubility, metabolic stability, and bioavailability.^13^ However, few synthetic strategies have been developed in asymmetric synthesis of triflouromethylated indolin-2-ones in the last decade.^14^ In 2016, the research group of Zhao and Hu reported the removal of Boc using trifluoroacetic acid (TFA) by intramolecular aminolysis of chiral dihydrocoumarin to obtain oxindole derivative in 98% yield and 97% ee, but resulted in decreased diastereoselectivity (2.5 : 1 dr) (Scheme 1a).^15^ In addition, the research group of Deng and Zhao disclosed the asymmetric synthesis of triflouromethylated oxindole derivative from chiral spirooxindole-containing γ-lactone with HCl/MeOH in high yield and enantioselectivity albeit very low diastereoselectivity (1.2 : 1) (Scheme 1b).^16^ Herein, we describe a new robust spirobiindane-derived squaramide, and demonstrate that this new chiral organocatalyst can be applied in highly stereoselective synthesis of triflouromethylated indolin-2-ones (Scheme 1c).

**Scheme 1:**
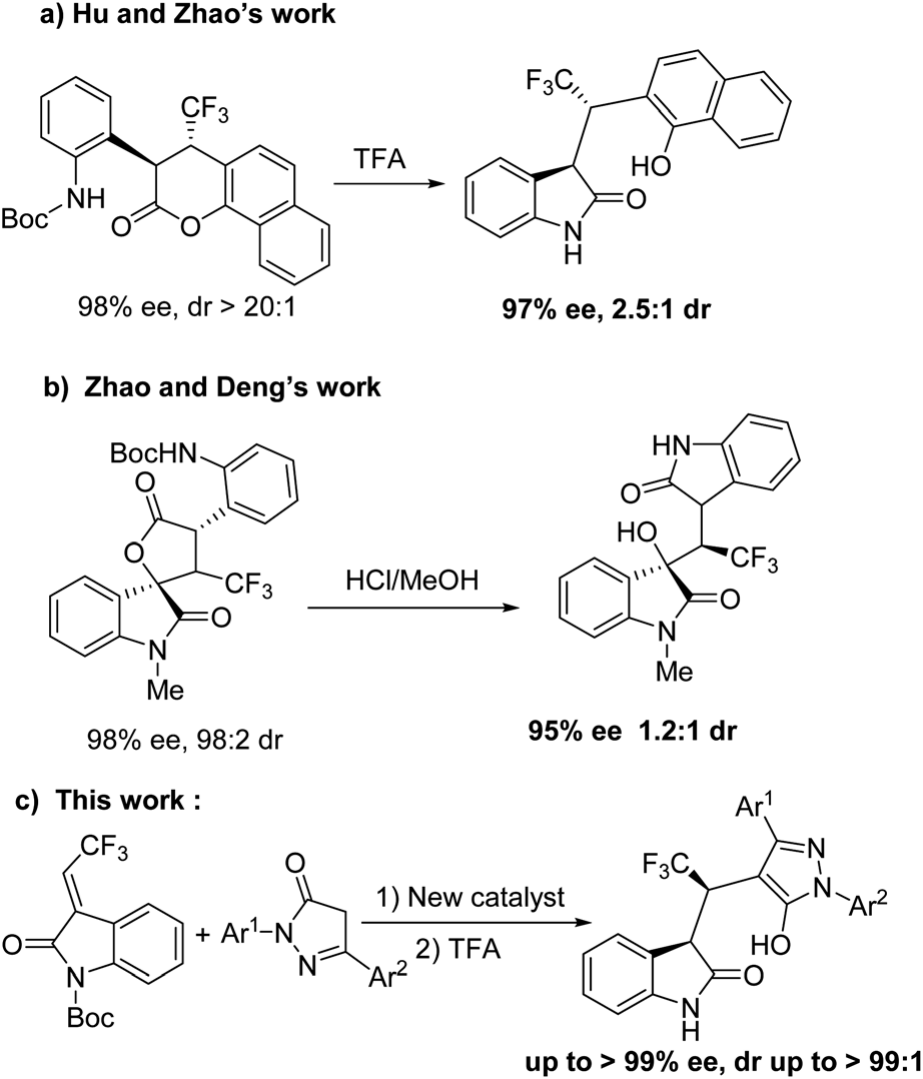
Asymmetric synthesis of triflouromethylated indolin-2-ones.

The new chiral spirobiindane-derived squaramide 9a was firstly prepared, as shown in Scheme 2. Following a modified procedure developed by our group,^6*b*,17^ we began the synthesis of 9a with hexamethyl-tetrahydro-1,1′-spirobi[indene]-6,6′-diol (6,6′-HMSIOL), which was prepared by acid-catalyzed rearrangement of bisphenol C. Then, (*R*)-1 was obtained in 92% yield with >99% ee by inclusion resolution using *N*-benzyl cinchonidine chloride as the resolution reagent in toluene. The chiral spirocyclic dialdehyde 4 was prepared from (*R*)-1 by Duff reaction, trifuloromesylation reaction and reduction reaction. The spirocyclic bisbromide 6 was obtained in 83% yield in two steps *via* reduction reaction followed by bromination. Finally, the desired chiral hexamethyl-1,1′-spirobiindane-based squaramide 9a was efficiently prepared in two steps by cyclization with (*R*,*R*)-1,2-diaminocyclohexane followed by an addition reaction with compound 8. To evaluate the effectiveness of our newly developed spirobiindane-derived squaramide organocatalyst 9a, we examined its performance in the first diastereo- and enantioselective conjugate addition of pyrazol-3-ones 11a to 3-trifluoroethylidene oxindoles 10a (Scheme 3). We observed that 2 mol% of 9a catalyzed this reaction smoothly in toluene at room temperature in 16 hours to give the desired product 12a in 70% yield with poor stability. Compound 12a slowly decomposes to produce complex mixtures, possibly due to the presence of both acid-sensitive the *N*-Boc group and acidic proton in the molecule. Followed by removing of Boc group using TFA, the stable product 13a could be obtained in 97% yield with high stereoselectivity (93% ee, 91 : 9 dr).

**Scheme 2:**
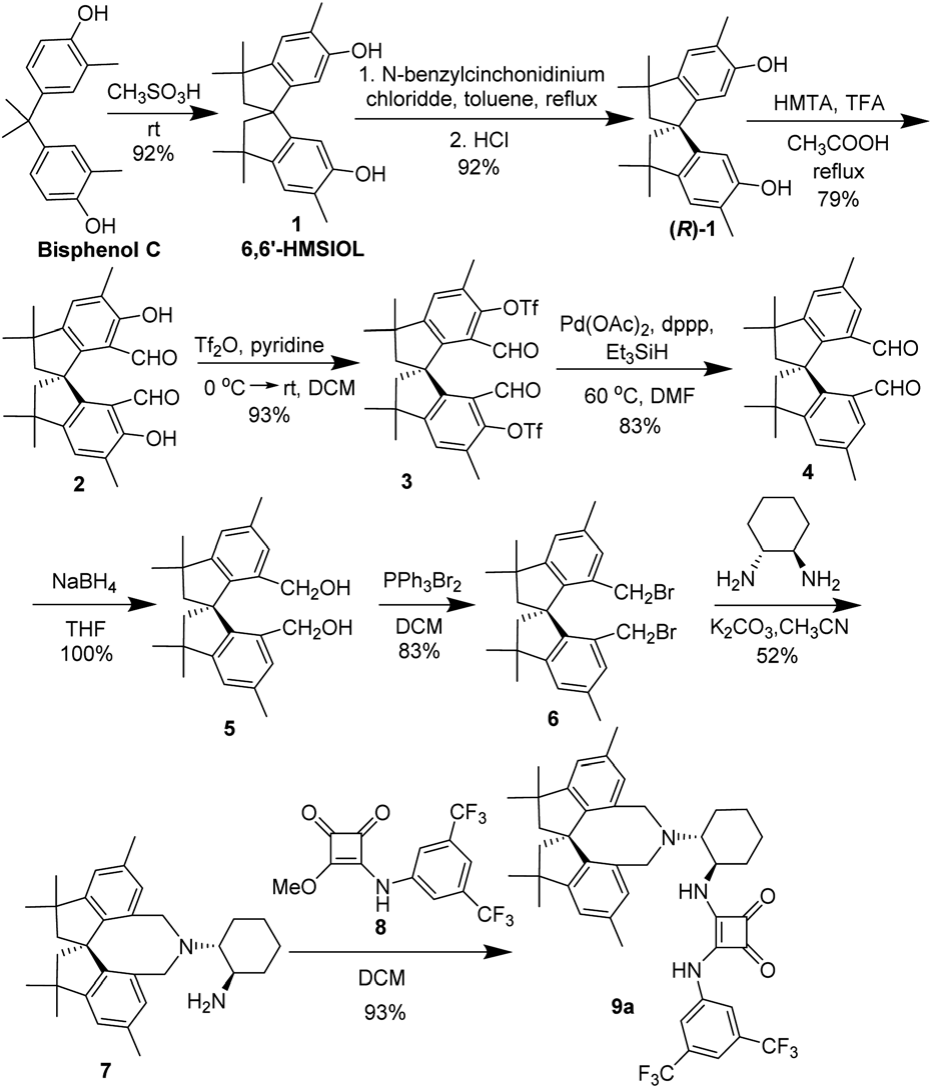
Synthesis of spirobiindane-based chiral bifunctional amine-squaramide organocatalyst.

**Scheme 3:**
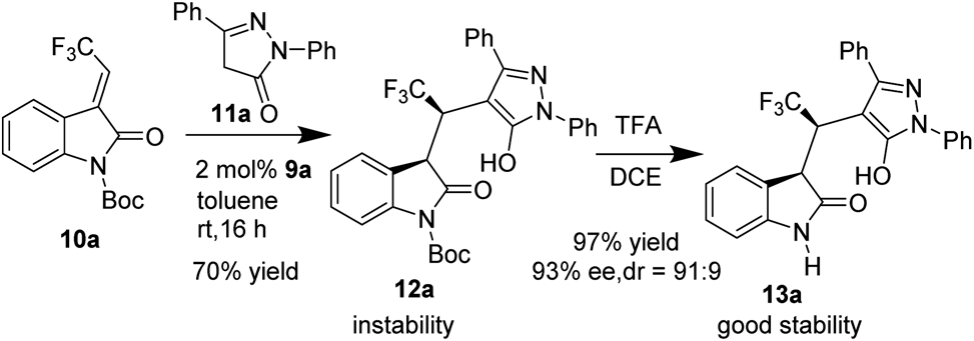
Initial catalytic test with new catalyst 9a.

As shown in Table 1, we then examined different solvents, such as DCM, DCE, 1,4-dioxane and tetrahydrofuran, and found that DCE was the optimal solvent for this asymmetric addition reaction to provide the desired product 13a in 91% yield with 95% ee and 94 : 6 dr (entries 1–5). Next, we investigated the catalyst loading at 1 mol%, but the yield was decreased to 67% and enantioselectivity was also decreased slightly to 88% ee (entry, 6). Furthermore, when the temperature was decreased to 0 °C, low yield (53%) and stereoselectivity (67% ee, 82 : 18 dr) were obtained (entry 7). In contrast, when the temperature was increased to 40 °C, the reaction rate improved but the corresponding enantioselectivity was lower (84% ee, entry 8). In addition, 3 Å molecular sieve (60 mg) was tested but gave lower enantioselectivity (80% ee, entry 9).

**Table 1:** Optimization of reaction conditions^a^

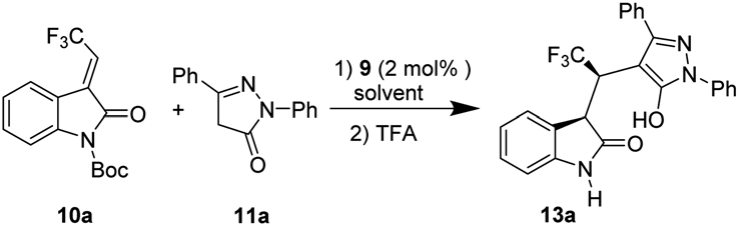						
Entry	Catalyst	Solvent	*T*/°C	Yield^b^ (%)	Dr^c^	Ee^d^ (%)
1	9a	Toluene	25	68	91 : 9	93
2	9a	DCM	25	84	93 : 7	88
3	9a	DCE	25	**91**	**94 : 6**	**95**
4	9a	1,4-Dioxane	25	Trace	—	—
5	9a	THF	25	Trace	—	—
6^e^	9a	DCE	25	67	96 : 4	88
7	9a	DCE	0	53	82 : 18	67
8^f^	9a	DCE	40	86	91 : 9	84
9^g^	9a	DCE	25	84	88 : 12	80
10	9b	DCE	25	74	93 : 7	80
11	9c	DCE	25	63	97 : 3	88
12	9d	DCE	25	71	97 : 3	5
13	9e	DCE	25	N.R	—	—
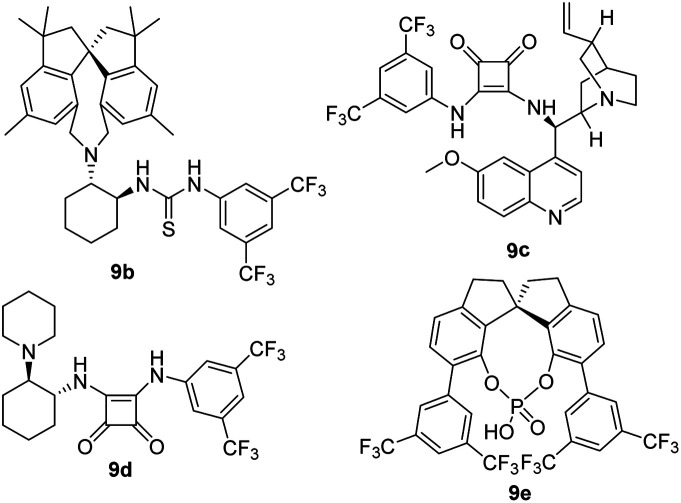						

a Reaction conditions: 10 (0.12 mmol), 11 (0.1 mmol) and catalyst 9 (2 mol%) in 1 mL solvent, 16 h.

b Isolated yield.

c Determined by ^19^F NMR in all cases using TFA as internal standard.

d Determined by chiral-phase HPLC analysis.

e With 9a (1 mol%).

f Reaction for 12 h.

g With 3 Å molecular sieves (60 mg) as an additive.

As a comparison, we also examined the known privileged chiral catalysts, such as chiral spirobiindane-derived thioureas (9b),^6*h*^ chiral squaramide catalysts (9c and 9d)^4^ and chiral phosphoric acid (9e)^6*a*^ (entries 10–13) in the model reaction to show their effectiveness in terms of reactivity and stereoselectivity. However, no better result was obtained. The newly developed chiral spirobiindane-derived squaramide catalyst 9a was the key to improving the stereoselectivity in the asymmetric conjugate addition for the synthesis of enantioenriched trifluoromethylated indolin-2-one. Thus, 2 mol% of catalyst 9a in DCE at 25 °C represented the optimal reaction conditions (entry 3).

Having the optimal reaction conditions in hand, we next examined the substrate scope (Table 2). In general, the reaction was applicable to a wide range of pyrazol-3-one derivatives 11, and different electronic properties and positions of the substituents on the aromatic ring of the substrates (11a–k) were all tolerated to give the corresponding products (13a–k) in good to excellent enantioselectivities (83–95% ee), and excellent diastereoselectivity (>10 : 1 dr). For example, when Cl group was substituted at different positions (*o*-, *m*- and *p*-) of the aromatic ring Ar^1^ on pyrazol-3-one, the corresponding products (13b–d) were obtained in moderate to high yields (51–91%) with excellent enantioselectivities (90–92% ee). When the electron-donating group Me was present in *para* and *meta* positions of Ar^1^ on pyrazol-3-one, the desired chiral products (13e and 13f) were also obtained in high yields and excellent stereoselectivities. Moreover, pyrazol-3-one with OMe group in *ortho* (11g) and *para* (11h) positions of Ar^2^ as the substrates, delivered the corresponding products in moderate yields (13g, 69%; 13h, 73%) and high enantioselectivity (13g, 94% ee; 13h, 96% ee). While the Cl group was attached to the *para*, *meta* and *ortho* positions of Ar^2^, the reaction proceeded smoothly and afforded the corresponding products 13i–k with good to excellent enantioselectivities (84–90% ee).

**Table 2:** Substrate scope^a^

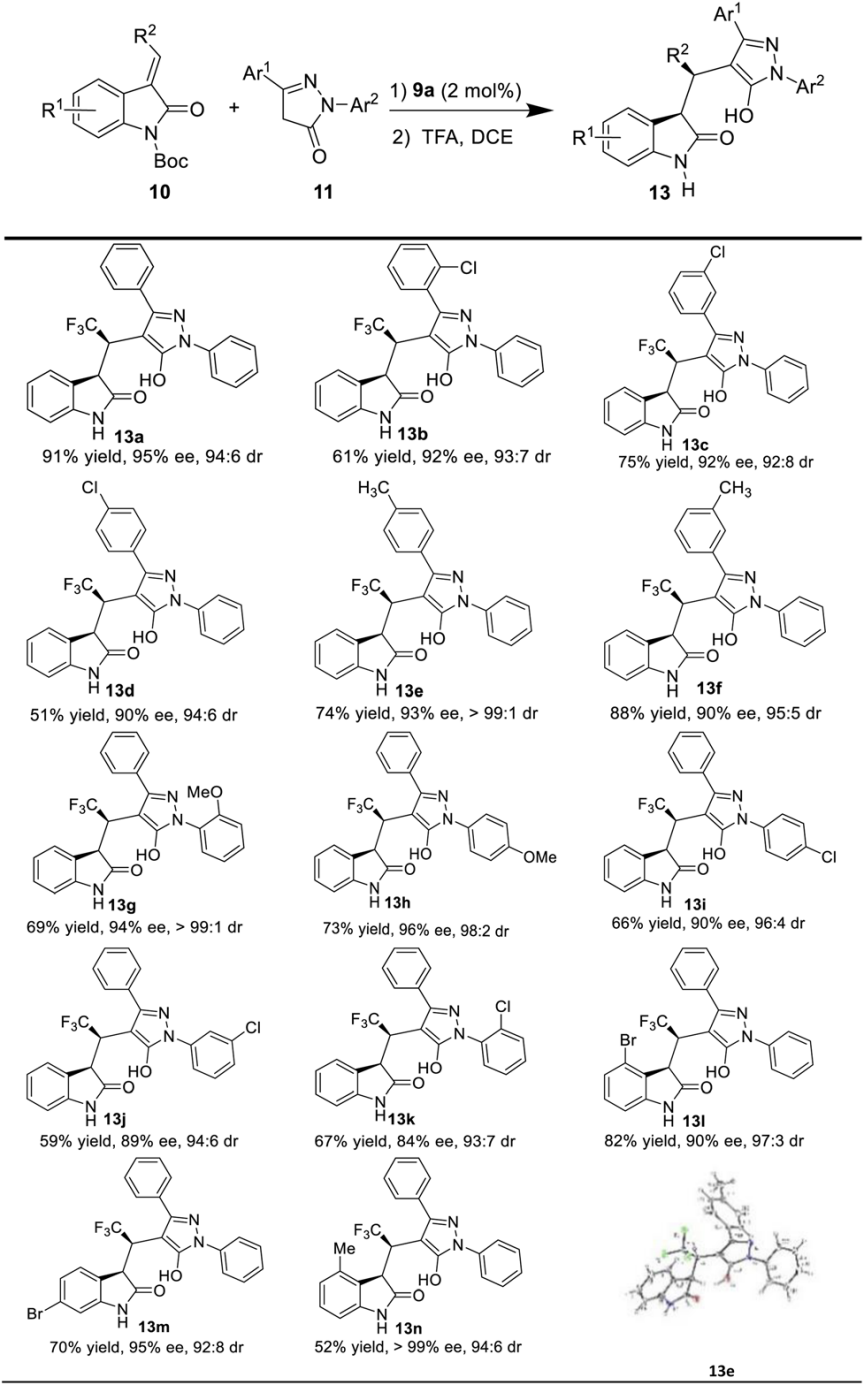

a Under the optimal conditions. Product 13 was obtained in isolated yield. The ee was determined by chiral-HPLC analysis. The dr was determined by ^19^F NMR.

We also evaluated the scope of the reaction with respect to the 3-ethyleneoxindole substrates 10, as shown in Table 1. To our delight, the substrate with bromo group was well tolerated, and the triflouromethylated indolin-2-one was isolated in good yield with high enantioselectivity (13l, 90% ee, dr = 97 : 3; 13m, 95% ee, 92 : 8 dr). Interestingly, 10d with a methyl group also worked well to afford the desired product 13n with good stereoselectivities (>99% ee, 94 : 6 dr) under the optimal reaction conditions. Moreover, the absolute configuration of the chiral indolin-2-one derivatives 13e was determined by the X-ray crystallographic analysis of a single crystal,^18^ and other products 13 were assigned by analogy.

In addition, 3-methylidene oxindoles (10e and 10f) bearing CO_2_Me or CO_2_Bn were also investigated under optimized conditions (Scheme 4). We were pleased to find the corresponding products were obtained with excellent enantioselectivities (13o, 95% ee; 13p, 98% ee) and high diastereoselectivies (13o, 95 : 5 dr; 13p, 91 : 9 dr) although in low yields.

**Scheme 4:**
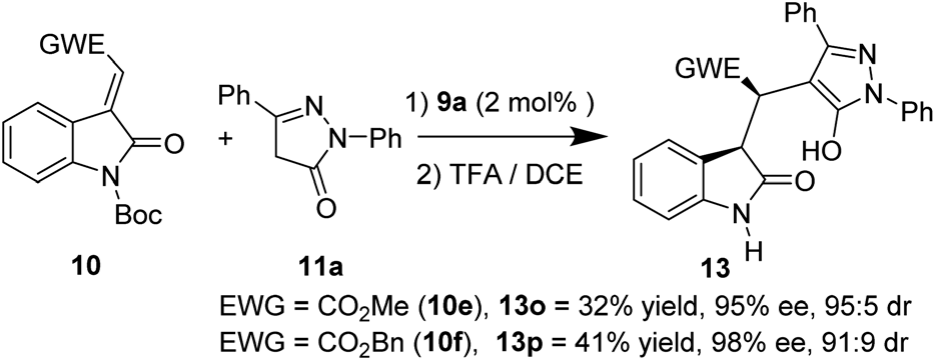
Study of 10 with different EWGs of the methyleneoxindoles.

On the basis of our above experimental observations and previous reported elegant works,^2–4^ a proposed reaction mechanism is elucidated in Fig. 1. The two squaramide N–H bonds of catalyst activated carbonyl of 3-ethylidene oxindole 10a*via* hydrogen bonding; concurrently, the enolized pyrazolone 11a′ form hydrogen bonding with tertiary amine moiety of catalyst. Sequentially, tertiary amine abstracts proton from 11a′ and pyrazol-5-ol 11a′ would attack the C_β_-position (Re-face) of 10a*via* the asymmetric Michael addition reaction. The formed carbanion then gains a proton to give the corresponding product 12a’ and then subjected to tautomerization to form the desired product 12a. Then, 13a could be obtained followed by removing of Boc group with trifiuoroacetic acid.

**Fig. 1:**
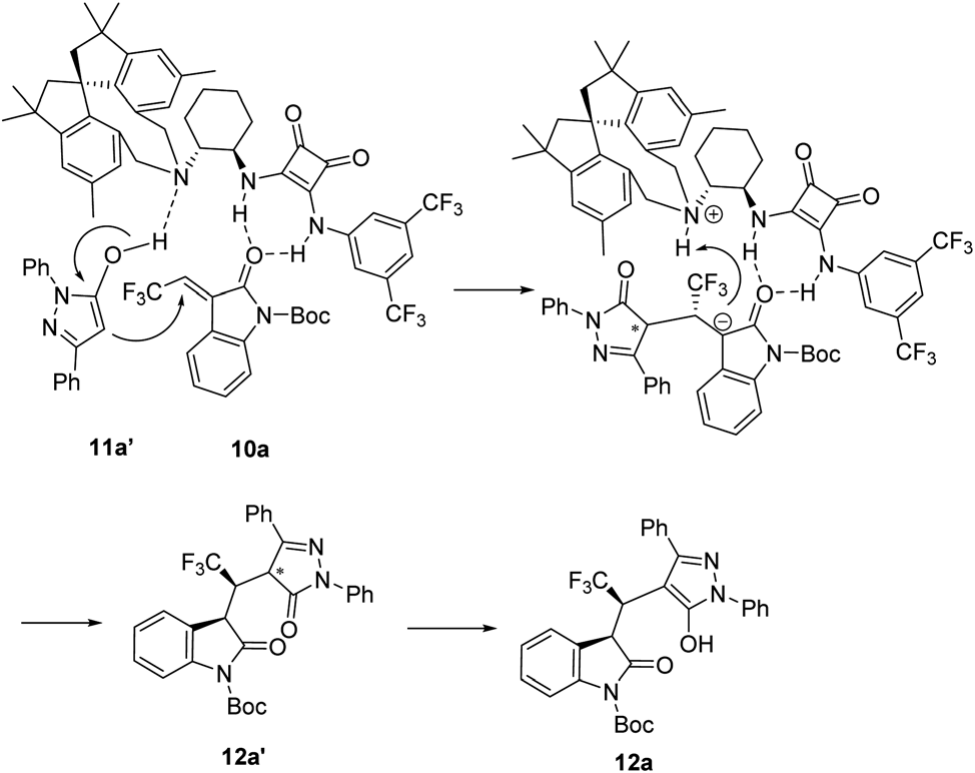
Proposed reaction mechanism.

## Conclusions

In summary, we have developed the first organocatalytic highly diastereo- and enantioselective conjugated addition reaction of pyrazol-3-ones with 3-trifluoroethylidene oxindoles. Under mild reaction conditions, the enantioenriched triflouromethylated indolin-2-ones bearing adjacent tertiary chiral centers were obtained in moderate to good yields with high to excellent diastereo- and enantioselectivities. The newly developed chiral spirobiindane-derived squaramide catalyst is the key point to improve the stereoselectivity.

## Experimental

### General information

All reactions were carried out in oven-dried glassware with magnetic stirring under ambient conditions. Unless otherwise noted, all reagents were purchased from commercial supplies and used without further purification, and all solvents were dried and purified according to standard methods prior to use. Substrates 10 (ref. 19) and 11 (ref. 20) were synthesized according to the literature methods. ^1^H NMR, ^13^C NMR, ^19^F NMR spectra were recorded on Bruker AVANCE III 400 MHz spectrometer instrument at 400 MHz for ^1^H NMR, 101 MHz for ^13^C NMR, 376 MHz for ^19^F NMR spectrometer; Bruker AVANCE III 500 MHz spectrometer instrument at 500 MHz for ^1^H NMR and 126 MHz for ^13^C NMR spectrometer; Bruker AVANCE III 600 MHz spectrometer instrument at 600 MHz for ^1^H NMR, 154 MHz for ^13^C NMR, 564 MHz for ^19^F NMR spectrometer, respectively. The chemical shifts (*δ*) were quoted in parts per million (ppm) downfield relative to internal standard TMS (0.0 ppm) and referenced to solvent peaks in the NMR solvent (CDCl_3_ = *δ* 7.26 ppm; *δ* 77.16 ppm; D_6_-DMSO = *δ* 2.50 ppm; *δ* 40.00 ppm; TFA = *δ* −76.55 ppm). Spin multiplicity were reported using the following abbreviations: s = singlet, d = doublet, t = triplet, dd = doublet of doublet, td = triplet of doublet, m = multiplet. Infrared spectra were recorded on an ATR-FTIR spectrometer. ESI-HRMS were recorded on a Water Micromass GCT Premier mass spectrometer. EI-HRMS were recorded on a Waters GCT Premier mass spectrometer. Optical rotations were measured on a PerkinElmer Model 341 polarimeter at 20 °C. Enantiomeric excess (ee) were measured by chiral HPLC analysis.

### Procedure for synthesis and resolution of 6,6′-HMSIOL (1)

Bisphenol C (50 g) was dissolved in methanesulfonic acid (250 mL), and the mixture was stirred at room temperature for 3 days. Then, additional 100 mL methanesulfonic acid was added to the reaction mixture, and the reaction ran for another 1 day. The reaction mixture was poured into the crushed ice and filtered, and the solid cake was washed with saturated solution of sodium bicarbonate and water. The residue was recrystallized with ethyl acetate/petroleum ether followed by ethanol/water, and dried to afford the white solid 1 (20.1 g, 92% yield). Then, a suspension of 1 (5 g, 15 mmol) and (8*S*,9*R*)-(−)-*N*-benzylcinchonidinium chloride (3.75 g, 9 mmol) in toluene (100 mL) was refluxed for 2 hours. A white solid was collected by filtration after the suspension was cooled slowly to room temperature, and then the above procedure was repeated once more. The solid precipitate was washed twice with toluene (30 mL) and dried in vacuum to afford the diastereomeric complex, which was added ethyl acetate (50 mL) and 1 M HCl (50 mL) to dissolve. The organic layer was separated, washed with saturated solution of brine, dried over anhydrous Na_2_SO_4_. Enantiomerically pure (*R*)-1 was afforded after the removal of the solvent.

#### (*R*)-3,3,3′,3′,5,5′-Hexamethyl-2,2′,3,3′-tetrahydro-1,1′-spirobi[indene]-6,6′-diol [(*R*)-1]^17^

White solid (2.30 g, 92% yield, >99% ee). HPLC analysis: Chiralpak AD-H (hexane/i-PrOH = 90/10, 0.8 mL min^−1^, 220 nm), *t*_R_ (major) 11.9 min, *t*_R_ (minor) 13.8 min. ^1^H NMR (500 MHz, CDCl_3_) *δ* 6.89 (s, 2H), 6.14 (s, 2H), 5.11 (s, 2H), 2.30 (d, *J* = 13.0 Hz, 2H), 2.23 (s, 6H), 2.14 (d, *J* = 13.0 Hz, 2H), 1.36 (s, 6H), 1.31 (s, 6H) ppm. ^13^C NMR (126 MHz, CDCl_3_) *δ* 153.1, 149.7, 144.7, 124.2, 122.9, 110.3, 59.7, 57.3, 43.1, 31.9, 30.7, 16.2 ppm.

### Procedure for synthesis of (*R*)-2

Hexamethylenetetramine (HMTA, 11.2 g, 80 mmol) was added to the solution of (*R*)-1 (3.4 g, 10 mmol) in trifluoroacetic acid (TFA, 120 mL), and the yellow solution was stirred and refluxed overnight under nitrogen. Glacial acetic acid (120 mL) was added once more to the above reaction mixture, which continued to reflux for 3 days. Then, 4 M HCl (120 mL) was added after cooling to 95 °C, and the mixture was stirred for 5 hours. After cooling to room temperature, the mixture was poured into water and finally filtered to give the desired product (*R*)-2.

#### (*R*)-6,6′-Dihydroxy-3,3,3′,3′,5,5′-hexamethyl-2,2′,3,3′-tetrahydro-1,1′-spirobi[indene]-7,7′-dicarbaldehyde [(*R*)-2]^17^

Yellow solid (3.10 g, 79% yield). ^1^H NMR (500 MHz, CDCl_3_) *δ* 12.00 (s, 2H), 9.56 (s, 2H), 7.20 (s, 2H), 2.57 (d, *J* = 13.5 Hz, 2H), 2.38 (d, *J* = 13.5 Hz, 2H), 2.26 (s, 6H), 1.37 (s, 6H), 1.35 (s, 6H) ppm. ^13^C NMR (126 MHz, CDCl_3_) *δ* 194.8, 162.5, 149.7, 141.9, 132.8, 128.1, 113.9, 60.5, 57.9, 43.1, 32.2, 30.2, 15.8 ppm.

### Procedure for synthesis of (*R*)-3

Triflic anhydride (3.4 mL, 20 mmol) was added dropwise to a solution of (*R*)-2 (1.97 g, 5 mmol) and pyridine (3.3 mL, 40 mmol) in dichloromethane (40 mL) at 0 °C under a nitrogen atmosphere, and the mixture was stirred overnight at room temperature. The reaction mixture was washed sequentially with 5% aqueous HCl, saturated solution of brine, saturated solution of NaHCO_3_, and saturated solution of brine, and then was dried over anhydrous Na_2_SO_4_. After removal of the solvent, the residue was purified by flash chromatography on a silica gel column (ethyl acetate/petroleum ether = 1/50) to give the product (*R*)-3.

#### (*R*)-7,7′-Diformyl-3,3,3′,3′,5,5′-hexamethyl-2,2′,3,3′-tetrahydro-1,1′-spirobi[indene]-6,6′-diyl bis(trifluoromethanesulfonate) [(*R*)-3]^17^

White solid (3.05 g, 93% yield). ^1^H NMR (500 MHz, CDCl_3_) *δ* 9.79 (s, 2H), 7.35 (s, 2H), 2.51 (d, *J* = 12.8 Hz, 2H), 2.44 (s, 6H), 2.42 (d, *J* = 12.8 Hz, 2H), 1.50 (s, 6H), 1.40 (s, 6H) ppm. ^13^C NMR (126 MHz, CDCl_3_) *δ* 187.6, 154.7, 149.5, 147.4, 131.8, 130.9, 124.7, 118.6 (q, *J* = 320.3 Hz), 58.9, 57.7, 43.3, 32.6, 29.1, 17.0 ppm.

### Procedure for synthesis of (*R*)-4

Triethylsilane 3 (10.8 mL, 67.5 mmol) was added slowly to a solution of (*R*)-4 (2.96 g, 4.5 mmol), Pd(OAc)_2_ (203 mg, 0.9 mmol), and 1,3-bis(diphenylphosphino)propane (372 mg, 0.9 mmol) in DMF (150 mL) under nitrogen, and the reaction ran at 60 °C for 6 hours. After cooling to room temperature, the resulting mixture was diluted with ether and washed sequentially with water, saturated solution of NaHCO_3_, and saturated solution of brine. The organic layer was dried over anhydrous Na_2_SO_4_ and concentrated under reduced pressure. The residue was purified by flash chromatography (ethyl acetate/petroleum ether = 1/15) to afford product (*R*)-4.

#### (*R*)-3,3,3′,3′,5,5′-Hexamethyl-2,2′,3,3′-tetrahydro-1,1′-spirobi[indene]-7,7′-dicarbaldehyde [(*R*)-4]^17^

Yellow solid (1.34 g, 83% yield). ^1^H NMR (500 MHz, CDCl_3_) *δ* 9.56 (s, 2H), 7.53 (s, 2H), 7.25 (s, 2H), 2.56 (d, *J* = 13.2 Hz, 2H), 2.43 (d, *J* = 13.4 Hz, 2H), 2.41 (s, 6H), 1.45 (s, 6H), 1.40 (s, 6H) ppm. ^13^C NMR (126 MHz, CDCl_3_) *δ* 190.7, 153.6, 150.3, 138.4, 130.7, 129.6, 129.4, 60.0, 57.4, 43.6, 32.6, 29.7, 21.3 ppm.

### Procedure for synthesis of (*R*)-5

NaBH_4_ (0.6 g, 16 mmol) was added to a solution of (*R*)-4 (1.14 g, 3.2 mmol) in THF (30 mL) at 0 °C, and the mixture was stirred for 10 min, then at room temperature for 3 hours. After cooling to 0 °C, 100 mL of water was added to the reaction mixture, which was stirred for 8 hours. The resulting mixture was diluted with ether and washed sequentially with water. The organic layer was dried over anhydrous Na_2_SO_4_ and concentrated under reduced pressure. The residue was purified by flash chromatography (ethyl acetate/petroleum ether = 1/4) to afford product (*R*)-5.

#### (*R*)-(3,3,3′,3′,5,5′-Hexamethyl-2,2′,3,3′-tetrahydro-1,1′-spirobi [indene]-7,7′-diyl) dimethanol [(*R*)-5]

White solid (1.16 g, 100% yield). Mp 82–84 °C. [*α*]^20^_D_ = 10.7 (*c* = 1.00, CH_2_Cl_2_). ^1^H NMR (400 MHz, CDCl_3_) *δ* 7.10 (s, 2H), 6.95 (s, 2H), 4.14 (dd, *J* = 25.2, 11.7 Hz, 4H), 2.38 (d, *J* = 11.4 Hz, 8H), 2.36 (s, 2H), 2.18 (d, *J* = 13.3 Hz, 2H), 1.39 (s, 6H), 1.33 (s, 6H) ppm. ^13^C NMR (101 MHz, CDCl_3_) *δ* 152.5, 144.6, 138.1, 136.0, 130.1, 123.1, 60.8, 58.9, 57.1, 43.2, 32.8, 30.2, 21.6 ppm. IR (film): *γ* = 3323, 2953, 2925, 2860, 1748, 1609, 1463, 1382, 1361, 1308, 1254, 1167, 1147, 1021, 861, 773 cm^−1^. HRMS (ESI^+^) calcd for [C_25_H_32_NaO_2_]^+^, *m*/*z* 387.2295, found 387.2295.

### Procedure for synthesis of (*R*)-6

(*R*)-5 (1.16 g, 3.2 mmol) and triphenylphosphine dibromide (7.1 g, 16 mmol) were dissolved in CH_2_Cl_2_ (30 mL) and stirred for 3 hours under nitrogen atmosphere, and 100 mL of water was added to quench the reaction. The mixture was extracted with CH_2_Cl_2_, washed by saturated solution of brine, dried over anhydrous Na_2_SO_4_, concentrated under reduced pressure. The residue was purified by flash chromatography (ethyl acetate/petroleum ether = 1/50) to afford product (*R*)-6.

#### (*R*)-7,7′-Bis(bromomethyl)-3,3,3′,3′,5,5′-hexamethyl-2,2′,3,3′-tetrahydro-1,1′-spirobi[indene] [(*R*)-6]

White solid (1.19 g, 83% yield). Mp 240–242 °C. [*α*]^20^_D_ = 117.1 (*c* = 1.00, CH_2_Cl_2_). ^1^H NMR (400 MHz, CDCl_3_) *δ* 7.07 (s, 2H), 6.95 (s, 2H), 4.02 (d, *J* = 10.2 Hz, 2H), 3.89 (d, *J* = 10.2 Hz, 2H), 2.51 (d, *J* = 13.4 Hz, 2H), 2.36 (s, 3H), 2.34 (d, *J* = 16.1 Hz, 8H), 1.43 (s, 6H), 1.34 (s, 6H) ppm. ^13^C NMR (101 MHz, CDCl_3_) *δ* 152.9, 144.5, 138.4, 133.6, 132.1, 124.1, 57.5, 57.0, 43.5, 32.8, 30.4, 30.1, 21.5 ppm. IR (film): *γ* = 3446, 2953, 2923, 1856, 1609, 1464, 1382, 1361, 1311, 1230, 1208, 1168, 869, 768, 668 cm^−1^. HRMS (EI, GC-TOF) calcd for [C_25_H_30_Br_2_]^+^, *m*/*z* 488.0714, found 488.0713.

### Procedure for synthesis of 7

(*R*)-3 (1.56 g, 3.2 mmol), (1*R*,2*R*)-cyclohexane-1,2-diamine (1.46 g, 12.8 mmol) and K_2_CO_3_ (1.33 g, 9.6 mmol) were mixed in CH_3_CN (70 mL) under nitrogen atmosphere, and the mixture was refluxed overnight. After removal of solvent under reduced pressure, the resulting mixture was diluted with ether and washed sequentially with saturated solution of NaHCO_3_ and brine. The organic layer was dried over anhydrous Na_2_SO_4_ and concentrated under reduced pressure. The residue was purified by flash chromatography (ethyl acetate/petroleum ether = 1/6 + 5% triethylamine) to afford the desired product 7.

#### (1*R*,2*R*)-2-(2,4,4,7,7,9-Hexamethyl-4,5,6,7-tetrahydro-11*H*-diindeno[7,1-*cd*:1′,7′-*ef*]azocin-12(13*H*)-yl)cyclohexan-1-amine (7)

White solid (737 mg, 52%). Mp 84–86 °C. [*α*]^20^_D_ = 183.6 (*c* = 1.00, CH_2_Cl_2_). ^1^H NMR (400 MHz, CDCl_3_) *δ* 6.87 (d, *J* = 8.4 Hz, 4H), 3.85 (d, *J* = 12.9 Hz, 2H), 3.26 (d, *J* = 13.1 Hz, 5H), 2.97–2.85 (m, 1H), 2.49 (t, *J* = 9.8 Hz, 1H), 2.36 (d, *J* = 15.9 Hz, 2H), 2.34 (s, 6H), 2.12 (d, *J* = 9.6 Hz, 1H), 1.90 (d, *J* = 12.5 Hz, 2H), 1.66 (s, 2H), 1.49 (s, 6H), 1.24 (s, 6H), 1.19–1.12 (m, 2H) ppm. ^13^C NMR (101 MHz, CDCl_3_) *δ* 151.1, 146.3, 137.5, 131.0, 130.6, 122.3, 71.6, 57.7, 57.6, 51.9, 48.4, 41.8, 34.0, 32.6, 30.4, 28.6, 26.2, 25.0, 21.4 ppm. IR (film): *γ* = 3726, 3473, 2925, 1959, 1028, 669, 655, 417, 410 cm^−1^. HRMS (ESI^+^) calcd for [C_31_H_43_N_2_]^+^, *m*/*z* 443.3421, found 443.3421.

### Procedure for synthesis of 9a

7 (88.6 mg, 0.2 mmol) and 3-((3,5-bis(trifluoromethyl)phenyl)amino)-4-methoxycyclobut-ene-1,2-dione 8 (67.8 mg, 0.2 mmol) were dissolved in 5 mL CH_2_Cl_2_, and the mixture was allowed to stir at room temperature for 2 days. The precipitate was filtered and washed with cold CH_3_CN then dried in vacuum to afford the desired product 9a.

#### 3-((3,5-Bis(trifluoromethyl)phenyl)amino)-4-(((1*R*,2*R*)-2-(2,4,4,7,7,9-hexamethyl-4,5,6,7-tetrahydro-11*H*-diindeno[7,1-*cd*:1′,7′-*ef*]azocin-12(13*H*)-yl)cyclohexyl)amino)cyclobut-3-ene-1,2-dione (9a)

White solid (139 mg, 93% yield). Mp 234–236 °C. [*α*]^20^_D_ = 62.5 (*c* = 1.00, CH_2_Cl_2_). ^1^H NMR (600 MHz, DMSO-d_6_) *δ* 10.09 (s, 1H), 8.02 (s, 2H), 7.67 (s, 1H), 7.63 (s, 1H), 6.81 (d, *J* = 10.6 Hz, 3H), 4.23 (s, 1H), 3.59 (d, *J* = 13.0 Hz, 2H), 3.29 (d, *J* = 13.0 Hz, 2H), 2.72–2.65 (m, 1H), 2.50 (d, *J* = 1.5 Hz, 6H), 2.31–2.23 (m, 2H), 2.19 (s, 4H), 2.03 (d, *J* = 12.8 Hz, 1H), 1.67 (dd, *J* = 36.9, 12.7 Hz, 3H), 1.57 (d, *J* = 7.9 Hz, 1H), 1.40 (s, 6H), 1.34–1.22 (m, 2H), 1.14 (s, 6H) ppm. ^13^C NMR (151 MHz, DMSO-d_6_) *δ* 184.3, 179.8, 169.2, 162.2, 150.2, 145.6, 141.1, 136.5, 131.3 (q, *J* = 34.4 Hz), 130.3, 130.0, 123.2(q, *J* = 272.6 Hz), 121.7, 120.4, 118.0, 114.6, 67.0, 57.1, 54.9, 47.3, 41.2, 33.8, 32.2, 29.9, 27.8, 24.4, 24.1, 20.9 ppm. ^19^F NMR (564 MHz, DMSO-d_6_) *δ* −61.8 ppm. IR (film): *γ* = 3451, 2922, 2847, 1959, 1619, 1032, 495, 412, 403 cm^−1^. HRMS (ESI^+^) calcd for [C_43_H_46_F_6_N_3_O_2_]^+^, *m*/*z* 750.3489, found 750.3489.

### Procedure for synthesis of 12a

To a solution of *tert*-butyl (*E*)-2-oxo-3-(2,2,2-trifluoroethylidene) indoline-1-carboxylate 10a (37.6 mg, 0.12 mmol, 1.2 eq.) and 2,5-diphenyl-2,4-dihydro-3*H*-pyrazol-3-one 11a (23.6 mg, 0.1 mmol, 1 eq.) in dichloroethane (1 mL) was added catalyst 9a (2 mol%, 0.002 mmol, 0.02 eq.). The resulting mixture was stirred at 25 °C for 16 h. After the reaction was completed, 12a was isolated and purified quickly by preparative chromatographic plate (ethyl acetate/petroleum ether = 1/4).

#### (*R*,*R*)-*tert*-Butyl 2-oxo-3-(2,2,2-trifluoro-1-(5-hydroxy-1,3-diphenyl-1*H*-pyrazol-4-yl)ethyl)indoline-1-carboxylate (12a)

Yellow solid (53 mg, 92% yield). Mp 97–99 °C. ^1^H NMR (400 MHz, CDCl_3_) *δ* 11.23 (s, 1H), 7.91 (d, *J* = 8.0 Hz, 2H), 7.85 (d, *J* = 8.2 Hz, 1H), 7.60 (dd, *J* = 8.1, 1.3 Hz, 2H), 7.55 (t, *J* = 7.3 Hz, 2H), 7.52–7.49 (m, 1H), 7.47 (t, *J* = 7.9 Hz, 2H), 7.38–7.35 (m, 1H), 7.31 (t, *J* = 7.4 Hz, 1H), 7.22 (t, *J* = 7.4 Hz, 1H), 7.06 (t, *J* = 9.2 Hz, 1H), 4.40–4.26 (m, 1H), 4.15 (s, 1H), 1.66 (s, 9H) ppm. ^13^C NMR (101 MHz, CDCl_3_) *δ* 177.3, 152.0, 151.7, 148.3, 139.3, 138.7, 133.1, 129.4, 129.0 (q, *J* = 11.1 Hz), 126.7, 126.0, 125.6, 122.8, 122.7, 119.2, 115.6, 92.5, 86.1, 50.3, 42.2 (q, *J* = 28.3 Hz), 31.6, 30.3, 28.1, 28.1, 27.0 ppm. ^19^F NMR (376 MHz, CDCl_3_) *δ* −66.2 ppm. IR (film): *γ* = 3727, 3474, 2926, 1959, 1610, 1350, 1149, 669 cm^−1^. HRMS (ESI^+^) calcd for [C_30_H_26_F_3_N_3_NaO_4_]^+^, *m*/*z* 572.1768, found 572.1768.

### General procedure for synthesis of 13

To a solution of 10 (0.12 mmol, 1.2 eq.) and 11 (0.1 mmol, 1 eq.) in dichloroethane (1 mL) was added catalyst 9a (2 mol%, 0.002 mmol, 0.02 eq.). The resulting mixture was stirred at 25 °C for 16 h. Then, CF_3_COOH (114 mg, 1.0 mmol) was added at room temperature and the reaction mixture was stirred for 2 h. After the reaction was completed, saturated solution of sodium carbonate was added to quench the reaction. The mixture was extracted with ethyl acetate and washed with saturated solution of brine, and then the organic phase was separated and dried over anhydrous Na_2_SO_4_. The corresponding product was isolated and purified by preparative chromatographic plate (ethyl acetate/petroleum ether = 1/4) to afford the desired product 13.

#### (*R*)-3-((*R*)-2,2,2-Trifluoro-1-(5-hydroxy-1,3-diphenyl-1*H*-pyrazol-4-yl)ethyl)indolin-2-one (13a)

White solid (41 mg, 91% yield, 95% ee). Mp 184–185 °C. [*α*]^20^_D_ = −18.6 (*c* = 1.00, CH_2_Cl_2_). The enantiomeric excess was determined by HPLC (Daicel Chiralpak IC-3, hexane/i-PrOH = 95 : 5(v/v), *λ* = 254 nm, flow rate = 1.5 mL min^−1^, 25 °C): *t*_R_ (major) 8.28 min, *t*_R_ (minor) 6.60 min. ^1^H NMR (600 MHz, CDCl_3_) *δ* 12.20 (s, 1H), 8.49 (s, 1H), 7.92 (d, *J* = 7.9 Hz, 2H), 7.61 (d, *J* = 7.3 Hz, 2H), 7.53 (t, *J* = 7.4 Hz, 2H), 7.50–7.42 (m, 3H), 7.29 (dd, *J* = 14.7, 7.4 Hz, 2H), 7.12 (t, *J* = 7.4 Hz, 1H), 7.08 (d, *J* = 7.2 Hz, 1H), 6.91 (d, *J* = 7.7 Hz, 1H), 4.32 (q, *J* = 9.3 Hz, 1H), 4.05 (s, 1H) ppm. ^13^C NMR (101 MHz, CDCl_3_) *δ* 179.3, 152.2, 151.9, 139.7, 138.0, 137.8, 132.4, 129.0 (q, *J* = 5.5 Hz), 127.9, 126.9, 126.0 (q, *J* = 283.3 Hz), 124.0, 123.0, 123.0, 110.9, 92.8, 49.6, 41.2 (q, *J* = 28.4 Hz), 29.7, 21.3, 19.7 ppm. ^19^F NMR (564 MHz, CDCl_3_, TFA) *δ* −65.7 ppm. IR (film): *γ* = 3397, 2933, 2857, 1959, 1667, 1212, 1163, 1040, 796, 742, 691 cm^−1^. HRMS (ESI^+^) calcd for [C_25_H_19_F_3_N_3_O_2_]^+^, *m*/*z* 450.1424, found 450.1426.

#### (*R*)-3-((*R*)-1-(3-(2-Chlorophenyl)-5-hydroxy-1-phenyl-1*H*-pyrazol-4-yl)-2,2,2-trifluoroethyl)indolin-2-one (13b)

Yellow solid (33 mg, 61% yield, 92% ee). Mp 158–160 °C. [*α*]^20^_D_ = −82.0 (*c* = 1.00, CH_2_Cl_2_). The enantiomeric excess was determined by HPLC (Daicel Chiralpak IA, hexane/i-PrOH = 90 : 10 (v/v), *λ* = 254 nm, flow rate = 1 mL min^−1^, 25 °C): *t*_R_ (major) 9.96 min, *t*_R_ (minor) 14.55 min. ^1^H NMR (400 MHz, CDCl_3_) *δ* 12.90 (s, 1H), 8.34 (s, 1H), 7.84 (d, *J* = 7.6 Hz, 2H), 7.62 (dd, *J* = 8.0, 1.0 Hz, 1H), 7.56 (dd, *J* = 7.4, 1.6 Hz, 1H), 7.48 (dd, *J* = 7.9, 4.0, 2.0 Hz, 3H), 7.41 (td, *J* = 7.4, 1.1 Hz, 1H), 7.35 (t, *J* = 7.5 Hz, 2H), 7.32–7.28 (m, 1H), 7.17 (t, *J* = 7.4 Hz, 1H), 6.95 (d, *J* = 7.7 Hz, 1H), 4.23 (s, 1H), 3.85 (q, *J* = 9.4 Hz, 1H) ppm. ^13^C NMR (101 MHz, CDCl_3_) *δ* 179.6, 151.9, 150.1, 139.8, 138.7, 133.4, 133.2, 132.3, 130.6, 129.0 (q, *J* = 5.6 Hz), 127.6, 126.7, 126.0 (q, *J* = 283.4 Hz), 124.1, 123.8, 122.7, 110.7, 93.3, 60.6, 48.9, 41.7 (d, *J* = 28.6 Hz), 21.2, 14.3 ppm. ^19^F NMR (376 MHz, CDCl_3_, TFA) *δ* −66.7 ppm. IR (film): *γ* = 3064, 2964, 2920, 1959, 1683, 1266, 1145, 1115, 797, 753, 694 cm^−1^. HRMS (ESI^+^) calcd for [C_25_H_18_ClF_3_N_3_O_2_]^+^, *m*/*z* 484.1034, found 484.1038.

#### (*R*)-3-((*R*)-1-(3-(3-Chlorophenyl)-5-hydroxy-1-phenyl-1*H*-pyrazol-4-yl)-2,2,2-trifluoroethyl)indolin-2-one (13c)

Yellow solid (37 mg, 75% yield, 92% ee). Mp 120–122 °C. [*α*]^20^_D_ = −130.0 (*c* = 1.00, CH_2_Cl_2_). The enantiomeric excess was determined by HPLC (Daicel Chiralpak IC-3, hexane/i-PrOH = 90 : 10 (v/v), *λ* = 254 nm, flow rate = 1 mL min^−1^, 25 °C): *t*_R_ (major) 4.46 min, *t*_R_ (minor) 3.80 min. ^1^H NMR (400 MHz, CDCl_3_) *δ* 12.29 (s, 1H), 8.10 (s, 1H), 7.87 (d, *J* = 7.7 Hz, 2H), 7.60 (s, 1H), 7.48 (dd, *J* = 10.0, 6.0 Hz, 5H), 7.33 (t, *J* = 7.4 Hz, 2H), 7.17 (d, *J* = 4.9 Hz, 2H), 6.97 (d, *J* = 7.8 Hz, 1H), 4.34–4.21 (m, 1H), 4.07 (s, 1H) ppm. ^13^C NMR (101 MHz, CDCl_3_) *δ* 179.2, 152.2, 150.6, 139.7, 138.6, 135.1, 134.9, 130.3, 129.1 (q, *J* = 8.6 Hz), 128.0, 127.1, 126.9, 126.1 (q, *J* = 281.6 Hz), 124.3, 123.3, 122.8, 110.9, 92.6, 60.6, 49.6, 41.4 (q, *J* = 28.8 Hz), 21.2, 14.3 ppm. ^19^F NMR (376 MHz, CDCl_3_, TFA) *δ* −65.9 ppm. IR (film): *γ* = 3727, 2962, 2919, 1959, 1682, 1264, 1143, 1115, 795, 751, 691 cm^−1^. HRMS (ESI^+^) calcd for [C_25_H_18_ClF_3_N_3_O_2_]^+^, *m*/*z* 484.1034, found 484.1037.

#### (*R*)-3-((*R*)-1-(3-(4-Chlorophenyl)-5-hydroxy-1-phenyl-1*H*-pyrazol-4-yl)-2,2,2-trifluoroethyl)indolin-2-one (13d)

Yellow solid (25 mg, 51% yield, 90% ee). Mp 185–186 °C. [*α*]^20^_D_ = −98.2 (*c* = 1.00, CH_2_Cl_2_). The enantiomeric excess was determined by HPLC (Daicel Chiralpak IA, hexane/i-PrOH = 80 : 20 (v/v), *λ* = 254 nm, flow rate = 1 mL min^−1^, 25 °C): *t*_R_ (major) 4.88 min, *t*_R_ (minor) 4.30 min. ^1^H NMR (400 MHz, CDCl_3_) *δ* 8.23 (s, 1H), 7.78 (d, *J* = 7.7 Hz, 2H), 7.54 (s, 4H), 7.49 (t, *J* = 7.8 Hz, 2H), 7.38 (t, *J* = 7.5 Hz, 1H), 7.33 (t, *J* = 7.7 Hz, 1H), 7.18 (t, *J* = 7.6 Hz, 1H), 7.06 (d, *J* = 7.5 Hz, 1H), 6.98 (d, *J* = 7.8 Hz, 1H), 4.26–4.17 (m, 1H), 4.05 (s, 1H) ppm. ^13^C NMR (101 MHz, CDCl_3_) *δ* 179.1, 152.2, 150.8, 139.6, 138.6, 134.9, 131.8, 130.7, 130.4, 129.1 (q, *J* = 25.5 Hz), 128.0, 125.5 (q, *J* = 272.3 Hz), 123.3, 122.8, 110.9, 92.5, 49.7, 41.5 (q, *J* = 28.4 Hz), 29.8, 22.8, 14.3 ppm. ^19^F NMR (376 MHz, CDCl_3_, TFA) *δ* −65.9 ppm. IR (film): *γ* = 3445, 2963, 2919, 1959, 1688, 1262, 1111, 1016, 798, 752, 693 cm^−1^. HRMS (ESI^+^) calcd for [C_25_H_18_ClF_3_N_3_O_2_]^+^, *m*/*z* 484.1034, found 484.1037.

#### (*R*)-3-((*R*)-2,2,2-Trifluoro-1-(5-hydroxy-1-phenyl-3-(*p*-tolyl)-1*H*-pyrazol-4-yl)ethyl)indolin-2-one (13e)

Yellow solid (34 mg, 74% yield, 93% ee). Mp 190–191 °C. [*α*]^20^_D_ = −205.5 (*c* = 1.00, CH_2_Cl_2_). The enantiomeric excess was determined by HPLC (Daicel Chiralpak IC-3, hexane/i-PrOH = 90 : 10 (v/v), *λ* = 254 nm, flow rate = 1 mL min^−1^, 25 °C): *t*_R_ (major) 7.91 min, *t*_R_ (minor) 6.56 min. ^1^H NMR (400 MHz, CDCl_3_) *δ* 12.12 (s, 1H), 7.96 (s, 1H), 7.91 (d, *J* = 7.7 Hz, 2H), 7.47 (dd, *J* = 17.6, 8.0 Hz, 4H), 7.32 (dd, *J* = 18.3, 7.7 Hz, 4H), 7.19–7.10 (m, 2H), 6.96 (d, *J* = 7.7 Hz, 1H), 4.33 (q, *J* = 9.7 Hz, 1H), 4.06 (s, 1H), 2.46 (s, 3H) ppm. ^13^C NMR (151 MHz, CDCl_3_) *δ* 179.3, 152.6, 151.8, 139.7, 139.6, 136.8, 129.9, 129.1 (q, *J* = 19.0 Hz), 127.9, 126.0 (q, *J* = 283.4 Hz), 124.3, 123.7, 123.3, 111.1, 93.4, 60.8, 49.5, 41.1 (q, *J* = 28.7 Hz), 32.1, 29.8, 21.6 ppm. ^19^F NMR (376 MHz, CDCl_3_, TFA) *δ* −66.0 ppm.

IR (film): *γ* = 3471, 2917, 2849, 1959, 1689, 1261, 1107, 1021, 800, 742, 695 cm^−1^. HRMS (ESI^+^) calcd for [C_26_H_21_F_3_N_3_O_2_]^+^, *m*/*z* 464.158, found 464.1583.

#### (*R*)-3-((*R*)-2,2,2-Trifluoro-1-(5-hydroxy-1-phenyl-3-(*m*-tolyl)-1*H*-pyrazol-4-yl)ethyl)indolin-2-one (13f)

Yellow solid (33 mg, 88% yield, 90% ee). Mp 136–138 °C. [*α*]^20^_D_ = −136.5 (*c* = 1.00, CH_2_Cl_2_). The enantiomeric excess was determined by HPLC (Daicel Chiralpak IC-3, hexane/i-PrOH = 90 : 10 (v/v), *λ* = 254 nm, flow rate = 1 mL min^−1^, 25 °C): *t*_R_ (major) 5.78 min, *t*_R_ (minor) 4.40 min. ^1^H NMR (400 MHz, CDCl_3_) *δ* 12.23 (s, 1H), 8.79 (s, 1H), 7.91 (d, *J* = 7.7 Hz, 2H), 7.45 (dd, *J* = 13.2, 7.6 Hz, 3H), 7.42–7.35 (m, 2H), 7.30 (t, *J* = 7.4 Hz, 4H), 7.12 (d, *J* = 4.5 Hz, 2H), 6.88 (d, *J* = 7.7 Hz, 1H), 4.35 (dd, *J* = 18.7, 9.2 Hz, 1H), 4.05 (s, 1H), 2.45 (s, 3H) ppm. ^13^C NMR (101 MHz, CDCl_3_) *δ*^13^C NMR (101 MHz, CDCl_3_) *δ* 179.4, 152.2, 152.1, 139.8, 138.8, 133.1, 129.9, 129.5, 128.9 (q, *J* = 7.0 Hz), 128.2, 126.6, 126.3 (q, *J* = 276.3 Hz), 126.0, 124.1, 123.2, 122.8, 110.9, 92.7, 52.9, 49.8, 46.5, 41.3 (q, *J* = 28.6 Hz), 34.8, 21.6 (s) ppm. ^19^F NMR (376 MHz, CDCl_3_, TFA) *δ* −65.8 ppm. IR (film): *γ* = 3445, 2918, 2849, 1959, 1688, 1263, 1165, 1112, 796, 741, 693 cm^−1^. HRMS (ESI^+^) calcd for [C_26_H_21_F_3_N_3_O_2_]^+^, *m*/*z* 464.158, found 464.1584.

#### (*R*)-3-((*R*)-2,2,2-Trifluoro-1-(5-hydroxy-1-(2-methoxyphenyl)-3-phenyl-1*H*-pyrazol-4-yl)ethyl)indolin-2-one (13g)

Yellow solid (32 mg, 69% yield, 94% ee). Mp 126–128 °C. [*α*]^20^_D_ = −124.1 (*c* = 1.00, CH_2_Cl_2_). The enantiomeric excess was determined by HPLC (Daicel Chiralpak IC-3, hexane/i-PrOH = 90 : 10 (v/v), *λ* = 254 nm, flow rate = 1 mL min^−1^, 25 °C): *t*_R_ (major) 9.67 min, *t*_R_ (minor) 8.17 min. ^1^H NMR (400 MHz, CDCl_3_) *δ* 8.43 (s, 1H), 7.72 (d, *J* = 9.0 Hz, 2H), 7.59 (d, *J* = 6.8 Hz, 2H), 7.56–7.43 (m, 3H), 7.29 (d, *J* = 7.6 Hz, 1H), 7.13 (t, *J* = 7.4 Hz, 1H), 7.07 (d, *J* = 7.4 Hz, 1H), 6.98 (d, *J* = 9.0 Hz, 2H), 6.92 (d, *J* = 7.7 Hz, 1H), 4.30 (q, *J* = 9.4 Hz, 1H), 4.05 (s, 1H), 3.85 (s, 3H) ppm. ^13^C NMR (101 MHz, CDCl_3_) *δ* 179.2, 158.8, 152.0, 151.5, 139.6, 132.3, 131.0, 129.2, 129.1 (q, *J* = 6.8 Hz), 128.0, 127.1 (q, *J* = 280.7 Hz), 125.0, 124.6, 124.2, 123.3, 114.2, 110.9, 92.7, 55.7, 49.6, 49.3, 41.3 (q, *J* = 28.6 Hz), 29.8, 26.8 ppm. ^19^F NMR (376 MHz, CDCl_3_, TFA) *δ* −66.0 ppm. IR (film): *γ* = 3727, 2962, 2849, 1959, 1686, 1260, 1026, 799, 753, 669 cm^−1^. HRMS (ESI^+^) calcd for [C_26_H_21_F_3_N_3_O_3_]^+^, *m*/*z* 480.153, found 480.1533.

#### (*R*)-3-((*R*)-2,2,2-Trifluoro-1-(5-hydroxy-1-(4-methoxyphenyl)-3-phenyl-1*H*-pyrazol-4-yl)ethyl)indolin-2-one (13h)

Yellow solid (35 mg, 73% yield, 96% ee). Mp 138–140 °C. [*α*]^20^_D_ = −98.5 (*c* = 1.00, CH_2_Cl_2_). The enantiomeric excess was determined by HPLC (Daicel Chiralpak IA, hexane/i-PrOH = 75 : 25 (v/v), *λ* = 254 nm, flow rate = 1.2 mL min^−1^, 25 °C): *t*_R_ (major) = 5.12 min, *t*_R_ (minor) 7.01 min. ^1^H NMR (400 MHz, CDCl_3_) *δ* 8.52 (s, 1H), 7.61 (d, *J* = 6.9 Hz, 2H), 7.54–7.46 (m, 4H), 7.42 (t, *J* = 7.9 Hz, 1H), 7.29–7.23 (m, 1H), 7.12–7.01 (m, 4H), 6.89 (d, *J* = 7.7 Hz, 1H), 4.34 (dd, *J* = 18.8, 9.3 Hz, 1H), 4.08 (s, 1H), 3.84 (s, 3H) ppm. ^13^C NMR (101 MHz, CDCl_3_) *δ* 155.1, 153.2, 151.8, 140.0, 139.8, 132.4, 130.7, 129.1 (q, *J* = 21.0 Hz), 128.1, 126.2 (q, *J* = 282.5 Hz), 124.0, 123.4, 120.9, 112.6, 110.8, 88.4, 56.2, 49.5, 44.4, 41.3 (q, *J* = 28.9 Hz), 38.6, 27.6 ppm. ^19^F NMR (376 MHz, CDCl_3_, TFA) *δ* −66.0 ppm. IR (film): *γ* = 3065, 2962, 2922, 1959, 1686, 1258, 1171, 1029, 799, 739, 702 cm^−1^. HRMS (ESI^+^) calcd for [C_26_H_21_F_3_N_3_O_3_]^+^, *m*/*z* 480.153, found 480.1529.

#### (*R*)-3-((*R*)-1-(1-(4-Chlorophenyl)-5-hydroxy-3-phenyl-1*H*-pyrazol-4-yl)-2,2,2-trifluoroethyl)indolin-2-one (13i)

Yellow solid (31 mg, 66% yield, 89% ee). Mp 193–194 °C. [*α*]^20^_D_ = −98.2 (*c* = 1.00, CH_2_Cl_2_). The enantiomeric excess was determined by HPLC (Daicel Chiralpak IC-3, hexane/i-PrOH = 95 : 5 (v/v), *λ* = 254 nm, flow rate = 1 mL min^−1^, 25 °C): *t*_R_ (major) 7.09 min, *t*_R_ (minor) 6.02 min. ^1^H NMR (400 MHz, CDCl_3_) *δ* 8.34 (s, 1H), 7.77 (d, *J* = 8.8 Hz, 2H), 7.60–7.52 (m, 5H), 7.45 (d, *J* = 8.8 Hz, 2H), 7.32 (t, *J* = 7.7 Hz, 2H), 7.16 (t, *J* = 7.5 Hz, 1H), 7.06 (d, *J* = 7.5 Hz, 1H), 6.97 (d, *J* = 7.8 Hz, 1H), 4.28 (q, *J* = 8.8 Hz, 1H), 4.06 (s, 1H) ppm. ^13^C NMR (101 MHz, CDCl_3_) *δ* 179.3, 152.3, 152.2, 139.6, 137.4, 133.0, 132.0, 129.2, 129.0 (q, *J* = 8.4 Hz), 128.1, 126.2 (q, *J* = 282.4 Hz), 124.2, 123.6, 123.3, 110.9, 92.9, 51.9, 49.7, 41.4 (q, *J* = 29.2 Hz), 38.7, 29.8 ppm. ^19^F NMR (376 MHz, CDCl_3_, TFA) *δ* −65.9 ppm. IR (film): *γ* = 3065, 2597, 2360, 1953, 1684, 1266, 1141, 1111, 832, 750, 702 cm^−1^. HRMS (ESI^+^) calcd for [C_25_H_18_ClF_3_N_3_O_2_]^+^, *m*/*z* 484.1034, found 484.1035.

#### (*R*)-3-((*R*)-1-(1-(3-Chlorophenyl)-5-hydroxy-3-phenyl-1*H*-pyrazol-4-yl)-2,2,2-trifluoroethyl)indolin-2-one (13j)

Yellow solid (28 mg, 59% yield, 89% ee). Mp 147–148 °C. [*α*]^20^_D_ = −130.0 (*c* = 1.00, CH_2_Cl_2_). The enantiomeric excess was determined by HPLC (Daicel Chiralpak IC-3, hexane/i-PrOH = 92 : 8 (v/v), *λ* = 254 nm, flow rate = 1 mL min^−1^, 25 °C): *t*_R_ (major) 5.01 min, *t*_R_ (minor) 3.81 min. ^1^H NMR (400 MHz, CDCl_3_) *δ* 8.34 (s, 1H), 7.90 (s, 1H), 7.77 (d, *J* = 8.2 Hz, 1H), 7.56 (dd, *J* = 9.6, 3.4 Hz, 5H), 7.41 (t, *J* = 8.1 Hz, 1H), 7.35–7.29 (m, 2H), 7.15 (t, *J* = 7.6 Hz, 1H), 7.07 (d, *J* = 7.4 Hz, 1H), 6.97 (d, *J* = 7.8 Hz, 2H), 4.29 (q, *J* = 9.3 Hz, 1H), 4.06 (s, 1H) ppm. ^13^C NMR (101 MHz, CDCl_3_) *δ* 179.2, 152.5, 152.4, 139.3, 138.4, 134.7, 130.0, 129.4, 129.3, 129.0 (q, *J* = 10.1 Hz), 127.7, 127.3, 125.8 (q, *J* = 272.2 Hz), 124.3, 123.2, 121.0, 110.9, 92.0, 58.5, 49.4, 41.2, 41.0 (q, *J* = 30.8 Hz), 31.5 ppm. ^19^F NMR (376 MHz, CDCl_3_, TFA) *δ* −65.9 ppm. IR (film): *γ* = 3066, 2925, 2593, 1959, 1686, 1264, 1140, 1114, 798, 750, 702 cm^−1^. HRMS (ESI^+^) calcd for [C_25_H_18_ClF_3_N_3_O_2_]^+^, *m*/*z* 484.1034, found 484.1038.

#### (*R*)-3-((*R*)-1-(1-(2-Chlorophenyl)-5-hydroxy-3-phenyl-1*H*-pyrazol-4-yl)-2,2,2-trifluoroethyl)indolin-2-one (13k)

Yellow solid (32 mg, 67% yield, 84% ee). Mp 168–169 °C. [*α*]^20^_D_ = −82.0 (*c* = 1.00, CH_2_Cl_2_). The enantiomeric excess was determined by HPLC (Daicel Chiralpak IC-3, hexane/i-PrOH = 90 : 10 (v/v), *λ* = 254 nm, flow rate = 1 mL min^−1^, 25 °C): *t*_R_ (major) 10.03 min, *t*_R_ (minor) 12.78 min. ^1^H NMR (400 MHz, CDCl_3_) *δ* 8.11 (s, 1H), 7.61 (d, *J* = 7.0 Hz, 2H), 7.57–7.48 (m, 3H), 7.46–7.38 (m, 2H), 7.34–7.28 (m, 2H), 7.19–7.04 (m, 3H), 6.96 (d, *J* = 7.8 Hz, 1H), 4.34 (dd, *J* = 18.8, 9.3 Hz, 1H), 4.09 (s, 1H) ppm. ^13^C NMR (101 MHz, CDCl_3_) *δ* 179.2, 153.1, 152.5, 139.8, 135.5, 132.7, 130.8, 130.4, 129.9, 129.0 (q, *J* = 10.6 Hz), 127.7, 127.5, 126.2 (q, *J* = 282.5 Hz), 126.1, 124.0, 123.2, 110.9, 91.5, 53.6, 49.8, 41.3 (q, *J* = 28.6 Hz), 38.3, 29.8 ppm. ^19^F NMR (376 MHz, CDCl_3_, TFA) *δ* −65.9 ppm; IR (film): *γ* = 3064, 2963, 2605, 1959, 1683, 1262, 1141, 1113, 799, 752, 702 cm^−1^. HRMS (ESI^+^) calcd for [C_25_H_18_ClF_3_N_3_O_2_]^+^, *m*/*z* 484.1034, found 484.1035.

#### (*R*)-4-Bromo-3-((*R*)-2,2,2-trifluoro-1-(5-hydroxy-1,3-diphenyl-1*H*-pyrazol-4-yl)ethyl)indolin-2-one (13l)

Brown solid (43 mg, 82% yield, 90% ee). Mp 197–198 °C. [*α*]^20^_D_ = −36.7 (*c* = 1.00, CH_2_Cl_2_). The enantiomeric excess was determined by HPLC (Daicel Chiralpak IC-3, hexane/i-PrOH = 92 : 8 (v/v), *λ* = 254 nm, flow rate = 1 mL min^−1^, 25 °C): *t*_R_ (major) 4.55 min, *t*_R_ (minor) 7.17 min. ^1^H NMR (400 MHz, CDCl_3_) *δ* 12.46 (s, 1H), 8.68 (s, 1H), 7.85 (d, *J* = 7.9 Hz, 2H), 7.61 (d, *J* = 3.9 Hz, 2H), 7.46 (dd, *J* = 16.9, 6.4 Hz, 5H), 7.34 (t, *J* = 7.3 Hz, 1H), 7.11 (s, 2H), 6.80 (d, *J* = 3.0 Hz, 1H), 4.91 (q, *J* = 9.7 Hz, 1H), 3.88 (s, 1H) ppm. ^13^C NMR (101 MHz, CDCl_3_) *δ* 178.6, 151.9, 151.8, 138.6, 138.5, 132.9, 132.0, 130.0, 128.8 (q, *J* = 13.7 Hz), 126.6, 126.5, 126.0 (q, *J* = 289.6 Hz), 122.7, 116.6, 112.2, 92.3, 49.6, 46.7, 41.3 (q, *J* = 28.6 Hz), 29.7, 14.2 ppm. ^19^F NMR (376 MHz, CDCl_3_, TFA) *δ* −65.1 ppm. IR (film): *γ* = 3065, 2963, 2916, 1959, 1698, 1261, 1106, 1027, 800, 757, 696 cm^−1^. HRMS (ESI^+^) calcd for [C_25_H_18_BrF_3_N_3_O_2_]^+^, *m*/*z* 528.0529, found 528.0530.

#### (*R*)-6-Bromo-3-((*R*)-2,2,2-trifluoro-1-(5-hydroxy-1,3-diphenyl-1*H*-pyrazol-4-yl)ethyl)indolin-2-one (13m)

Brown solid (37 mg, 70% yield, 95% ee). Mp 125–126 °C. [*α*]^20^_D_ = −26.3 (*c* = 1.00, CH_2_Cl_2_). The enantiomeric excess was determined by HPLC (Daicel Chiralpak IC-3, hexane/i-PrOH = 90 : 10 (v/v), *λ* = 254 nm, flow rate = 1 mL min^−1^, 25 °C): *t*_R_ (major) 5.83 min, *t*_R_ (minor) 11.38 min; ^1^H NMR (400 MHz, CDCl_3_) *δ* 8.30 (s, 1H), 7.77 (d, *J* = 7.8 Hz, 2H), 7.55 (s, 5H), 7.49–7.46 (m, 2H), 7.39 (d, *J* = 7.3 Hz, 1H), 7.29 (s, 1H), 7.12 (s, 1H), 6.90 (d, *J* = 8.0 Hz, 1H), 4.23 (q, *J* = 8.8 Hz, 1H), 4.00 (s, 1H) ppm. ^13^C NMR (101 MHz, CDCl_3_) *δ* 179.0, 152.0, 151.9, 140.9, 138.7, 133.1, 129.0 (q, *J* = 5.7 Hz), 127.1, 127.0, 126.8, 125.8 (q, *J* = 275.8 Hz), 124.6, 122.8, 122.6, 114.3, 92.4, 49.4, 46.5, 41.4 (q, *J* = 28.6 Hz), 29.8, 14.3 ppm. ^19^F NMR (376 MHz, CDCl_3_, TFA) *δ* −65.8 ppm. IR (film): *γ* = 3471, 2962, 2925, 1959, 1691, 1261, 1106, 1026, 800, 757, 692 cm^−1^. HRMS (ESI^+^) calcd for [C_25_H_18_BrF_3_N_3_O_2_]^+^, *m*/*z* 528.0529, found 528.0530.

#### (*R*)-4-Methyl-3-((*R*)-2,2,2-trifluoro-1-(5-hydroxy-1,3-diphenyl-1*H*-pyrazol-4-yl)ethyl)indolin-2-one (13n)

Yellow solid (24 mg, 52% yield, >99% ee). Mp 108–109 °C. [*α*]^20^_D_ = −124.6 (*c* = 1.00, CH_2_Cl_2_). The enantiomeric excess was determined by HPLC (Daicel Chiralpak IC-3, hexane/i-PrOH = 90 : 10 (v/v), *λ* = 254 nm, flow rate = 1 mL min^−1^, 25 °C): *t*_R_ (major) 4.12 min, *t*_R_ (minor) 6.18 min. ^1^H NMR (400 MHz, CDCl_3_) *δ* 8.13 (s, 1H), 7.84 (d, *J* = 7.9 Hz, 2H), 7.63–7.44 (m, 7H), 7.34 (t, *J* = 7.4 Hz, 1H), 6.94 (s, 1H), 6.77 (s, 1H), 4.27 (dd, *J* = 18.4, 9.0 Hz, 1H), 4.03 (s, 1H), 2.36 (s, 3H) ppm. ^13^C NMR (101 MHz, CDCl_3_) *δ* 179.2, 152.0, 139.9, 138.8, 134.4, 133.2, 129.6, 129.2, 128.9 (q, *J* = 20.7 Hz), 128.4, 126.6, 126.5 (q, *J* = 282.7 Hz), 126.3, 126.1, 122.7, 108.3, 93.4, 49.8, 46.0, 39.9 (q, *J* = 28.0 Hz), 29.8, 17.7 ppm. ^19^F NMR (376 MHz, CDCl_3,_ TFA) *δ* −65.8 ppm. IR (film): *γ* = 3066, 2963, 2919, 1955, 1682, 1265, 1169, 1109, 775, 756, 701 cm^−1^. HRMS (ESI^+^) calcd for [C_26_H_21_F_3_N_3_O_2_]^+^, *m*/*z* 464.158, found 464.1577.

#### Methyl (*R*)-2-(5-hydroxy-1,3-diphenyl-1*H*-pyrazol-4-yl)-2-((*R*)-2-oxoindolin-3-yl)acetate (13o)

Yellow solid (14 mg, 32% yield, 95% ee). Mp 153–154 °C. [*α*]^20^_D_ = −65.1 (*c* = 1.00, CH_2_Cl_2_). The enantiomeric excess was determined by HPLC (Daicel Chiralpak IC-3, hexane/i-PrOH = 85 : 15 (v/v), *λ* = 254 nm, flow rate = 1 mL min^−1^, 25 °C): *t*_R_ (major) 13.20 min, *t*_R_ (minor) 10.44 min. ^1^H NMR (600 MHz, CDCl_3_) *δ* 11.37 (s, 1H), 8.03–7.80 (m, 3H), 7.60 (d, *J* = 6.8 Hz, 2H), 7.52–7.47 (m, 2H), 7.47–7.41 (m, 2H), 7.29 (t, *J* = 7.3 Hz, 1H), 7.24 (s, 1H), 7.10–6.98 (m, 2H), 6.92 (d, *J* = 7.8 Hz, 1H), 4.47 (d, *J* = 3.4 Hz, 1H), 3.97 (d, *J* = 3.4 Hz, 1H), 3.64 (s, 3H) ppm. ^13^C NMR (151 MHz, CDCl_3_) *δ* 184.0, 175.0, 153.8, 153.6, 143.1, 141.4, 136.0, 131.4, 131, 130.6, 128.9, 126.2, 125.9, 124.8, 112.9, 99.3, 55.6, 53.1, 44.8, 32.4, 25.3, 16.8 ppm. IR (film): *γ* = 3447, 2921, 2850, 1959, 1682, 1472, 1455, 1224, 753, 692, 668 cm^−1^. HRMS (ESI^+^) calcd for [C_26_H_22_N_3_O_4_]^+^, *m*/*z* 440.1606, found 440.1605.

#### Benzyl (*R*)-2-(5-hydroxy-1,3-diphenyl-1*H*-pyrazol-4-yl)-2-((*R*)-2-oxoindolin-3-yl)acetate (13p)

Yellow solid (21 mg, 41% yield, 98% ee). Mp 126–128 °C; [*α*]^20^_D_ = −45.7 (*c* = 1.00, CH_2_Cl_2_). The enantiomeric excess was determined by HPLC (Daicel Chiralpak IC-3, hexane/i-PrOH = 80 : 20 (v/v), *λ* = 254 nm, flow rate = 1 mL min^−1^, 25 °C): *t*_R_ (major) 12.23 min, *t*_R_ (minor) 8.89 min. ^1^H NMR (600 MHz, CDCl_3_) *δ* 8.50 (s, 1H), 7.82 (d, *J* = 7.9 Hz, 2H), 7.58 (d, *J* = 7.2 Hz, 2H), 7.51–7.39 (m, 5H), 7.34 (d, *J* = 7.9 Hz, 2H), 7.25 (s, 2H), 7.22 (t, *J* = 7.5 Hz, 1H), 7.13–6.94 (m, 4H), 6.81 (d, *J* = 7.7 Hz, 1H), 5.04 (q, *J* = 12.4 Hz, 2H), 4.51 (d, *J* = 3.1 Hz, 1H), 3.97 (s, 1H) ppm. ^13^C NMR (151 MHz, CDCl_3_) *δ* 184.3, 173.5, 154.4, 153.7, 143.1, 140.1, 137.5, 134.5, 131.6, 131.5, 131.4, 131.1, 130.9, 130.5, 129.8, 126.1, 126.0, 125.6, 113.4, 99.6, 70.2, 53.1, 44.5, 32.4, 25.4, 16.8 ppm. IR (film): *γ* = 3064, 2925, 2854, 2063, 1622, 1472, 1455, 1170, 752, 734, 696 cm^−1^. HRMS (ESI^+^) calcd for [C_32_H_25_N_3_NaO_4_]^+^, *m*/*z* 538.1739, found 538.1737.

## Conflicts of interest

There are no conflicts to declare.

## Supplementary Material

RA-012-D2RA05088A-s001

RA-012-D2RA05088A-s002
