# Supplementary material for: Organocatalytic diastereo- and enantioselective conjugate addition of pyrazol-3-ones to 3-trifluoroethylidene oxindoles with a newly developed squaramide catalyst
Source: RSC Adv. 2022 Sep 21;12(41):27012–21. doi: 10.1039/d2ra05088a (PMC9490773; doi:10.1039/d2ra05088a)
Supplement: RA-012-D2RA05088A-s001 [file RA-012-D2RA05088A-s001.pdf]

Electronic Supplementary Material (ESI) for RSC Advances.
This journal is © The Royal Society of Chemistry 2022

# Supporting Information

# Organocatalytic Diastereo- and Enantioselective Conjugate Addition of Pyrazol-3-ones to 3-Trifluoroethylidene Oxindoles with A Newly Developed Squaramide Catalyst

Zhao Han, Jiaping Jin, Alemayehu Gashaw Woldegiorgis and Xufeng Lin*
Department of Chemistry, Zhejiang University, Hangzhou 310027, P. R of China; E-mail: lxfok@zju.edu.cn

## Table of contents

1 Copies of NMR spectra . . . . . . . . . . . . . . . . . . . . . . . . . . . . . . . . . . . . . . . . . . . . . 2

2 Copies of HRMS Spectra . . . . . . . . . . . . . . . . . . . . . . . . . . . . . . . . . . . . . . . . . . . 35

3 Copies of HPLC spectra . . . . . . . . . . . . . . . . . . . . . . . . . . . . . . . . . . . . . . . . . . . . 46

4 X-ray data for compound 13e . . . . . . . . . . . . . . . . . . . . . . . . . . . . . . . . . . . . . . . 62

# 1 Copies of NMR spectra

**(*R*)-3,3,3',3',5,5'-hexamethyl-2,2',3,3'-tetrahydro-1,1'-spirobi[indene]-6,6'-diol [(*R*)-1]**

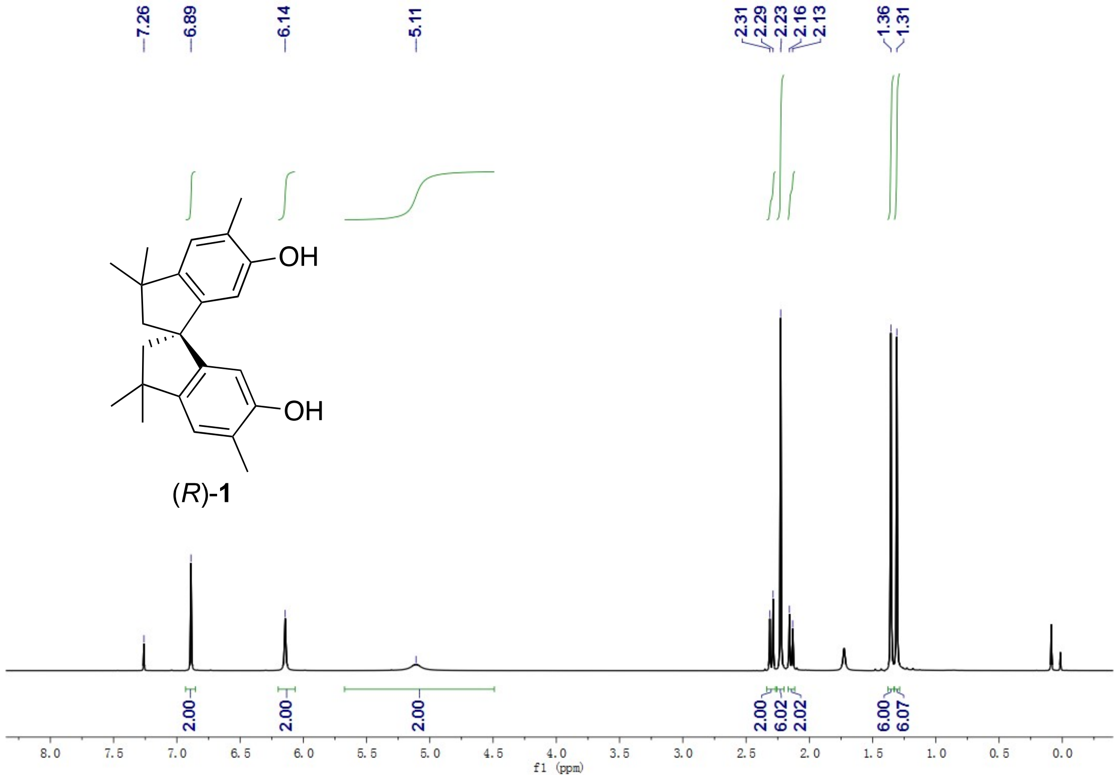


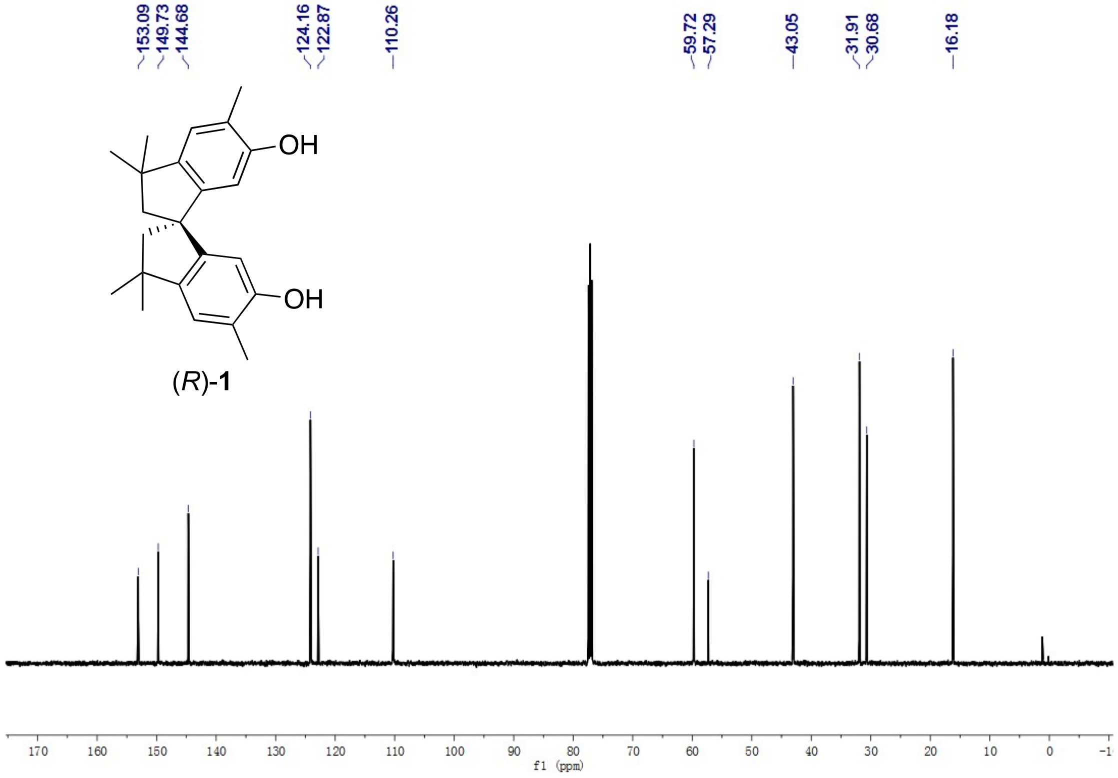

**(*R*)-6,6'-dihydroxy-3,3,3',3',5,5'-hexamethyl-2,2',3,3'-tetrahydro-1,1'-spirobi[indene]-7,7'-dicarbaldehyde [(*R*)-2]**

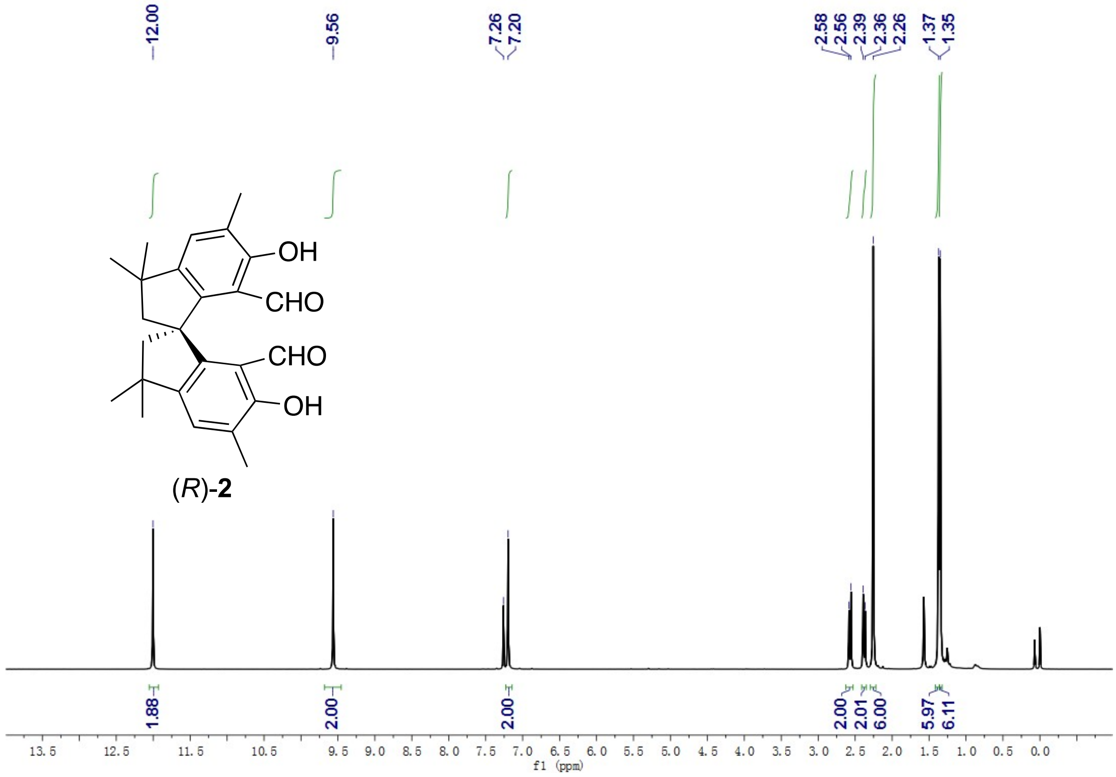


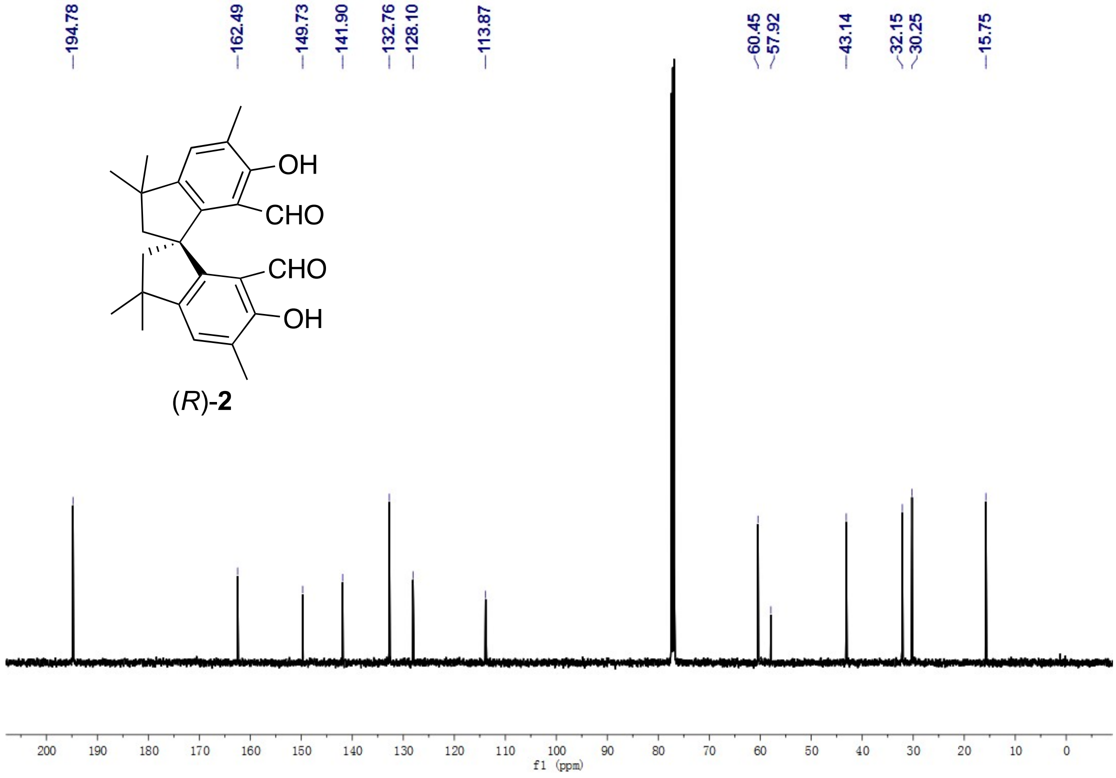

**(*R*)-7,7'-diformyl-3,3,3',3',5,5'-hexamethyl-2,2',3,3'-tetrahydro-1,1'-spirobi[indene]-6,6'-diyl bis(trifluoromethanesulfonate) [(*R*)-3]**

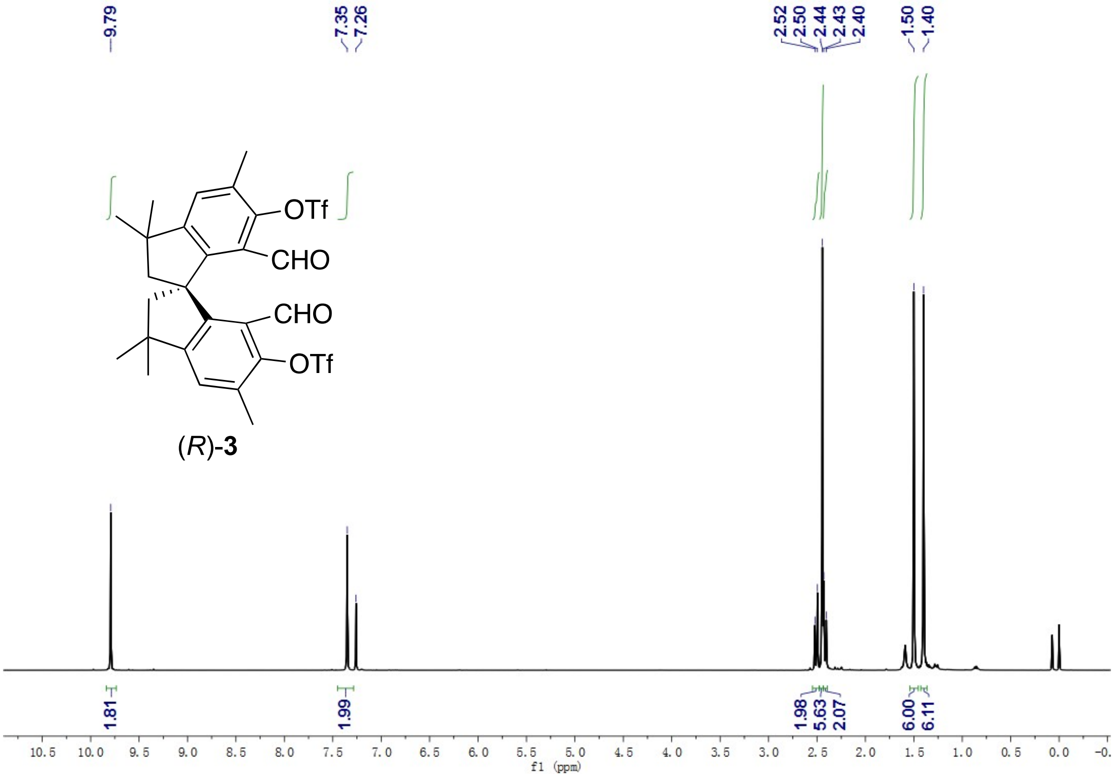


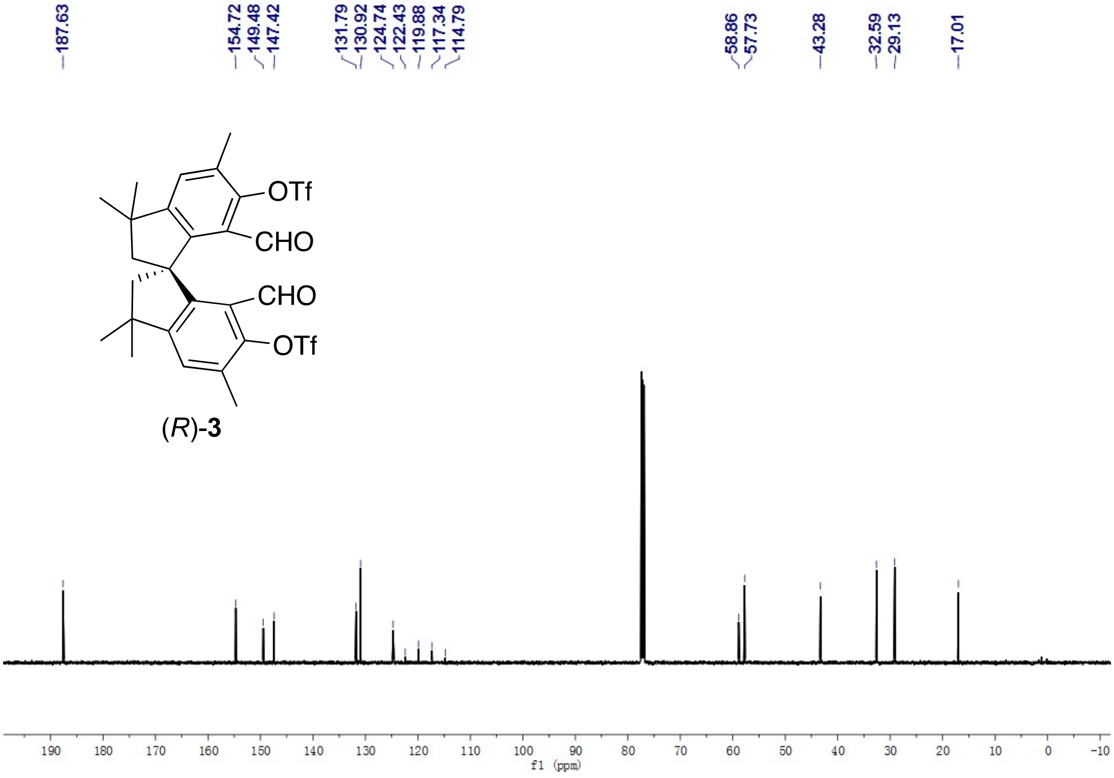

**(*R*)-3,3,3',3',5,5'-hexamethyl-2,2',3,3'-tetrahydro-1,1'-spirobi[indene]-7,7'-dicarbaldehyde [(*R*)-4]**

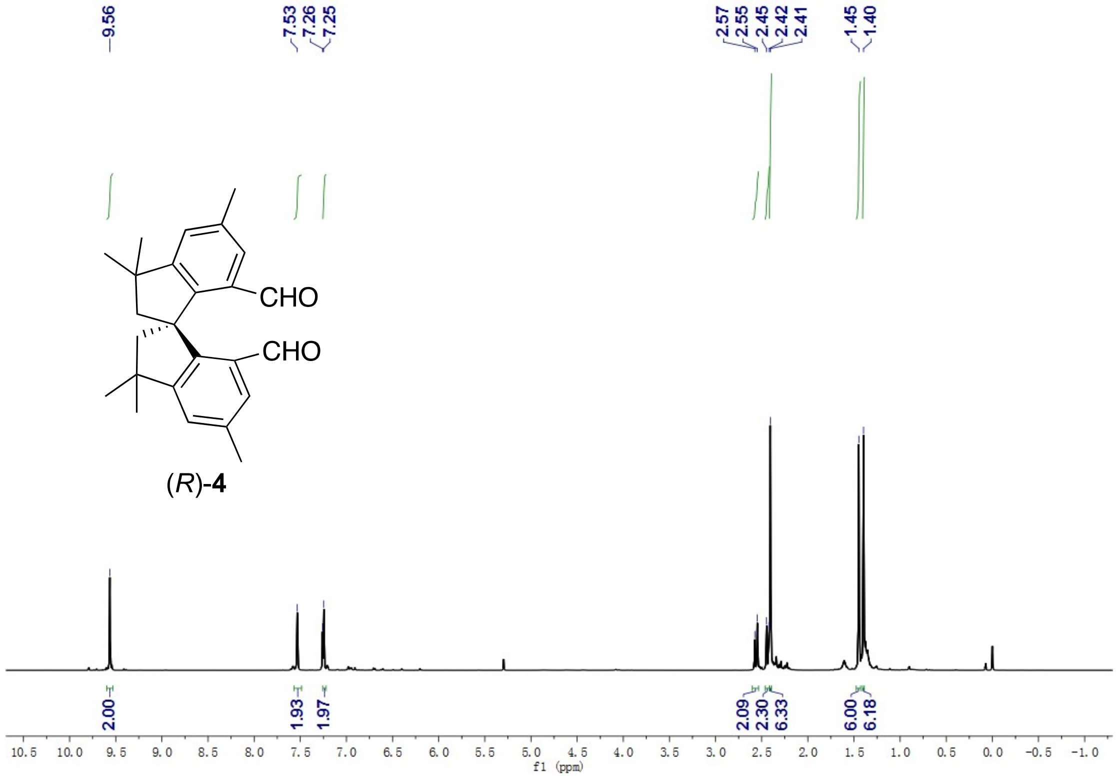


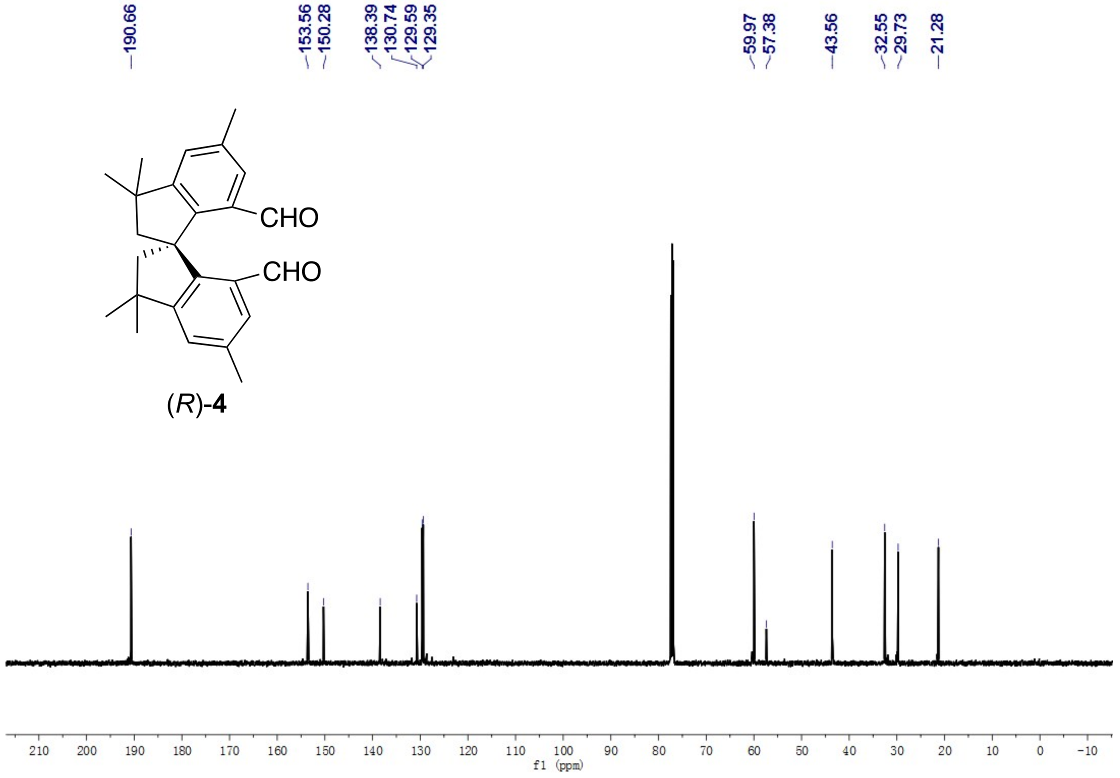

**(*R*)-(3,3,3',3',5,5'-hexamethyl-2,2',3,3'-tetrahydro-1,1'-spirobi[indene]-7,7'-diyl)dimethanol [(*R*)-5]**

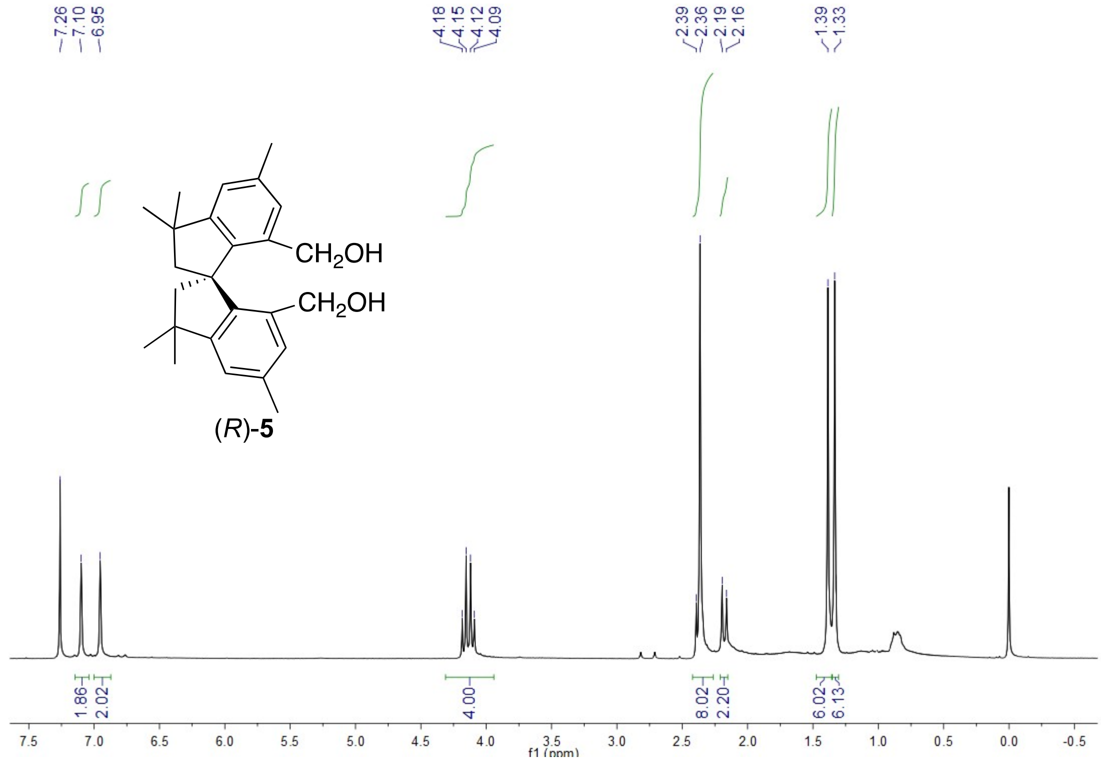


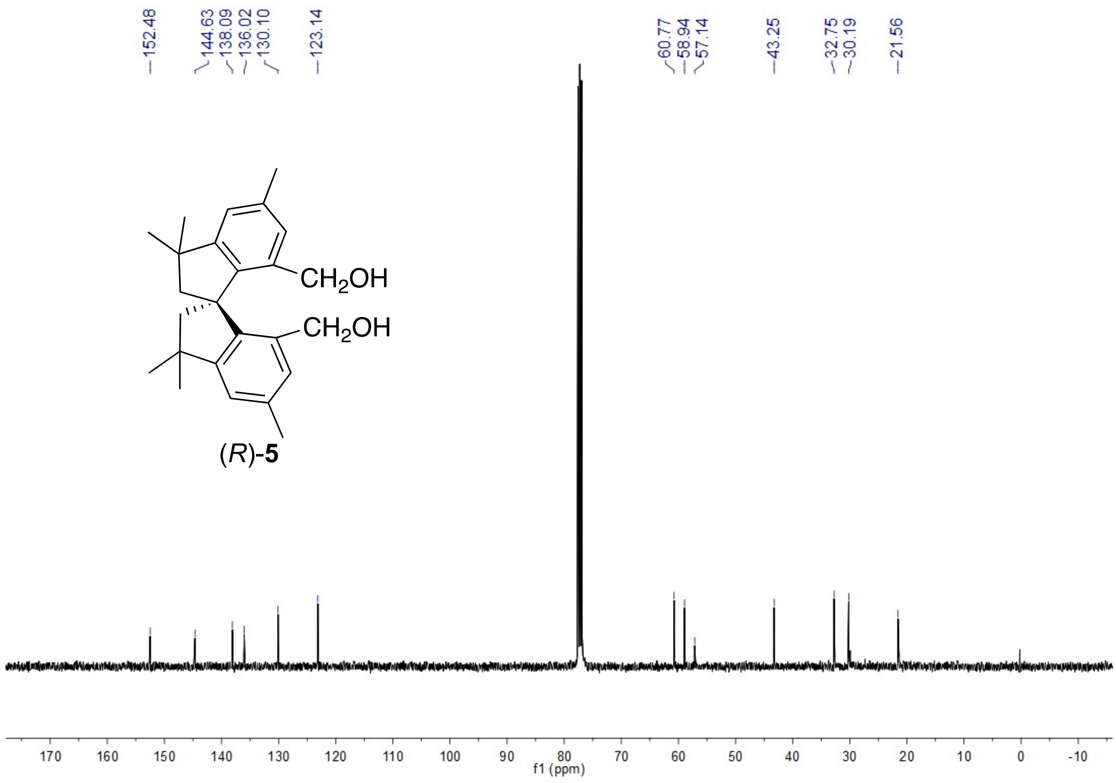

**(*R*)-7,7'-bis(bromomethyl)-3,3,3',3',5-pentamethyl-2,2',3,3'-tetrahydro-1,1'-spirobi[indene] [(*R*)-6]**

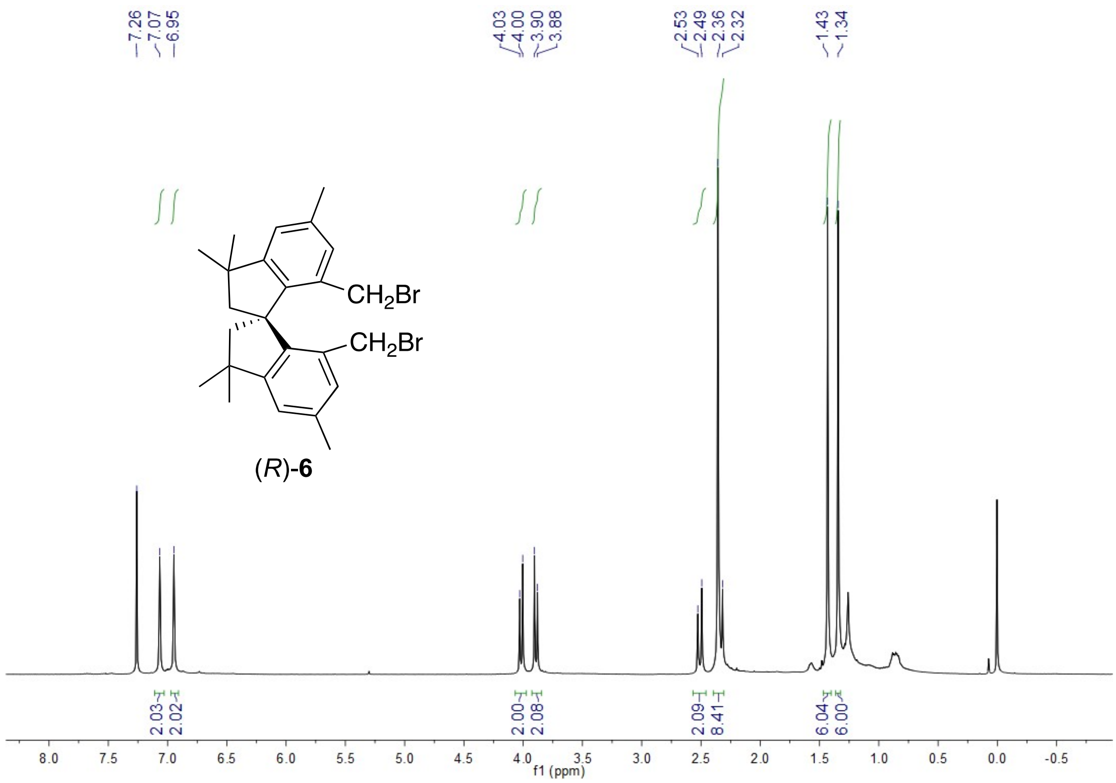


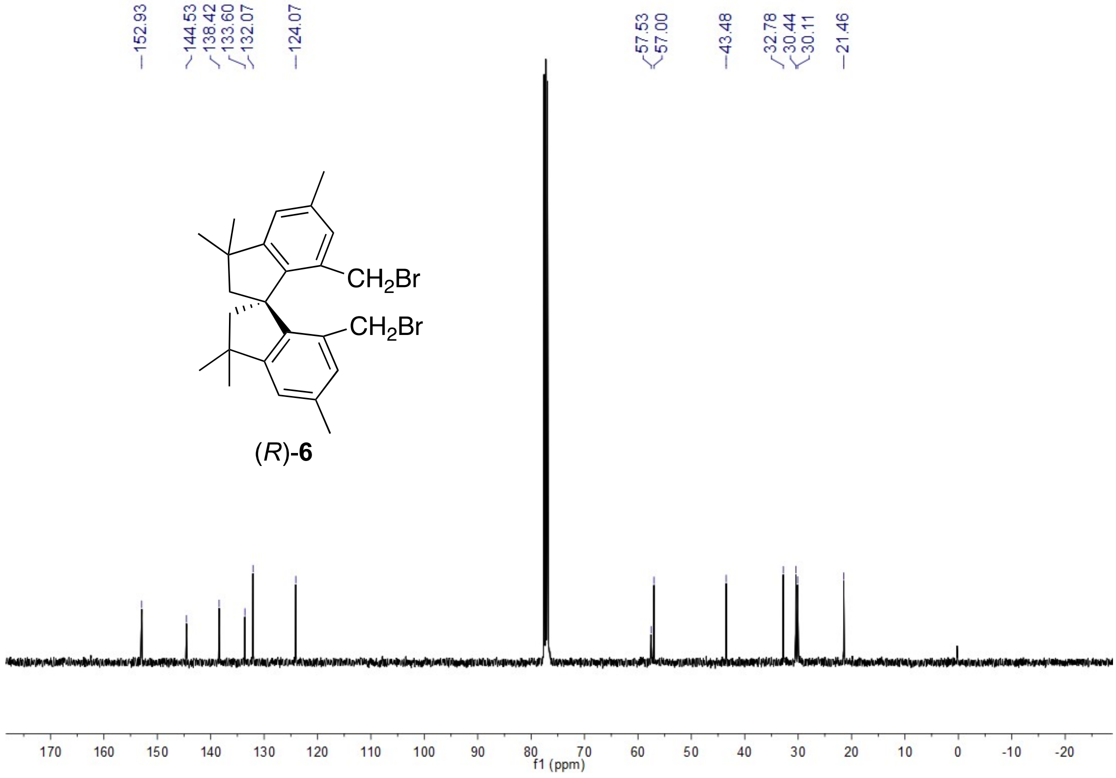

**(1R,2R)-2-(2,4,4,7,7,9-hexamethyl-4,5,6,7-tetrahydro-11H-diindeno[7,1-cd:1',7'-ef]azocin-12(13H)-yl)cyclohexan-1-amine (7)**

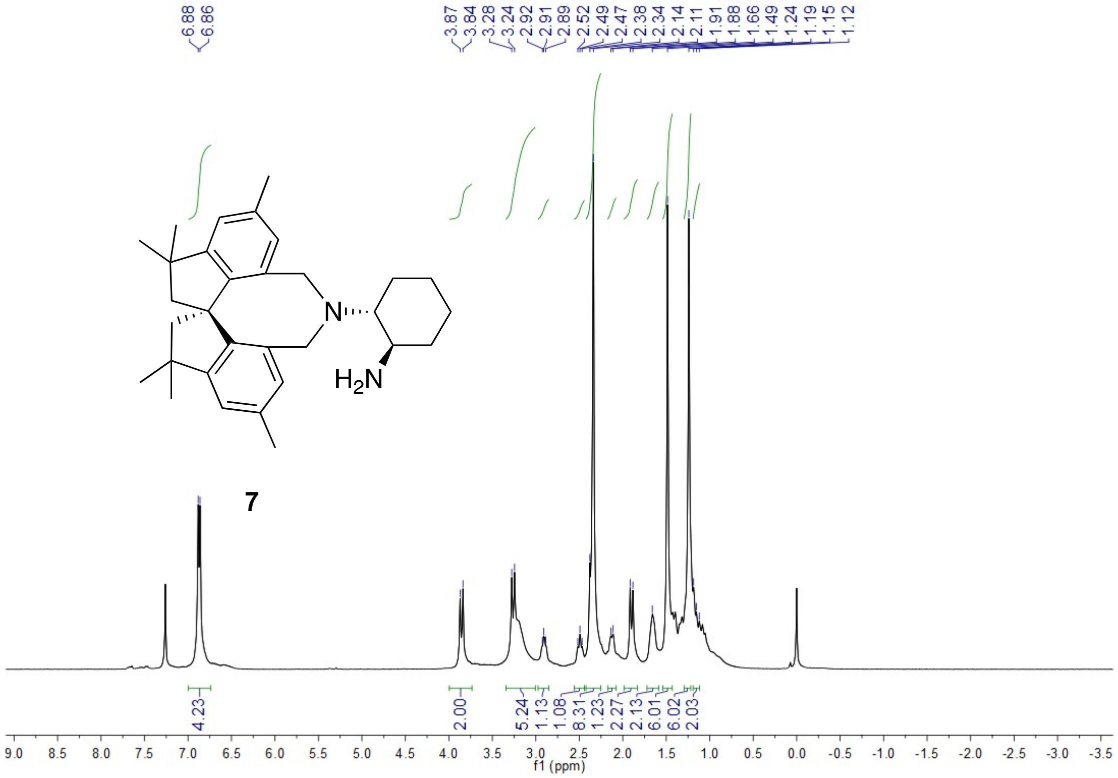


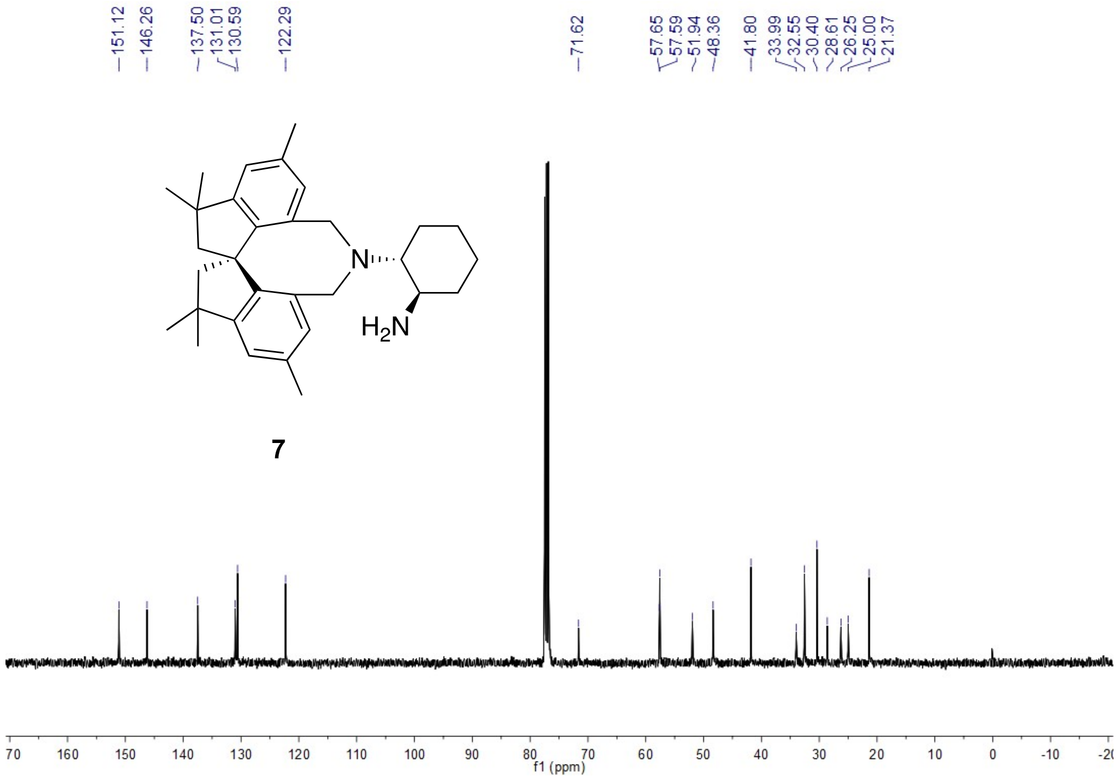

**3-((3,5-bis(trifluoromethyl)phenyl)amino)-4-(((1R,2R)-2-(2,4,4,7,7,9-hexamethyl-4,5,6,7-tetrahydro-11H-diindeno[7,1-cd:1',7'-ef]azocin-12(13H)-yl)cyclohexyl)amino)cyclobut-3-ene-1,2-dione (9a)**

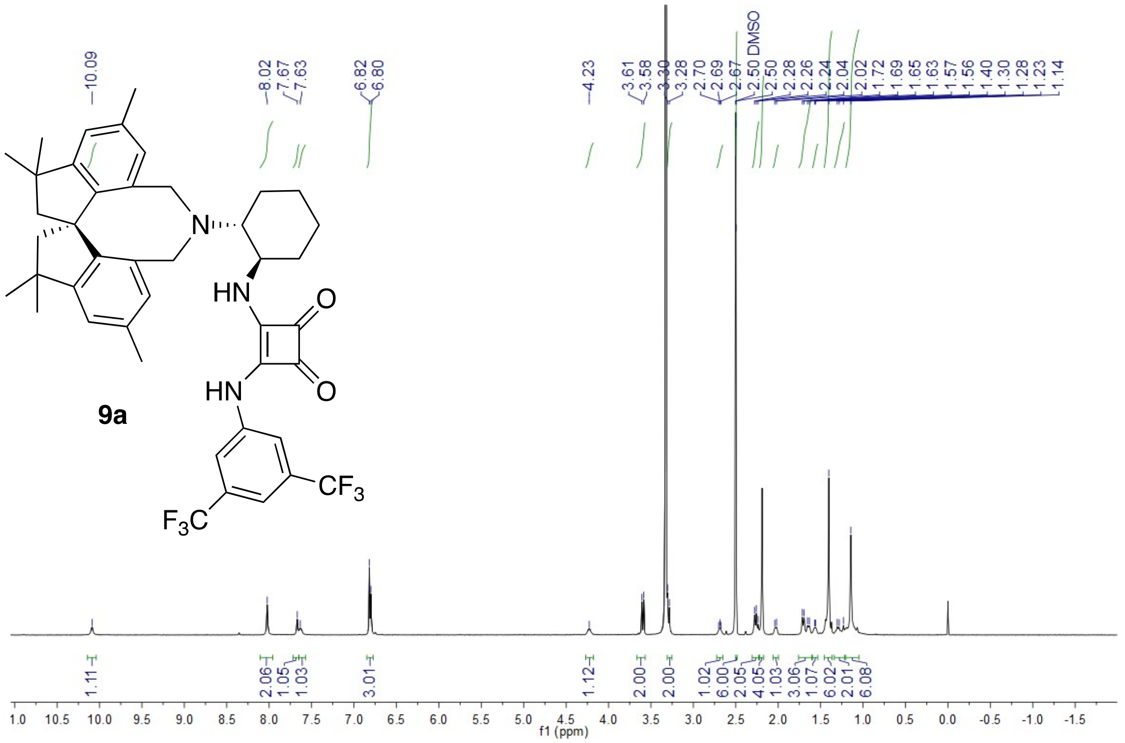

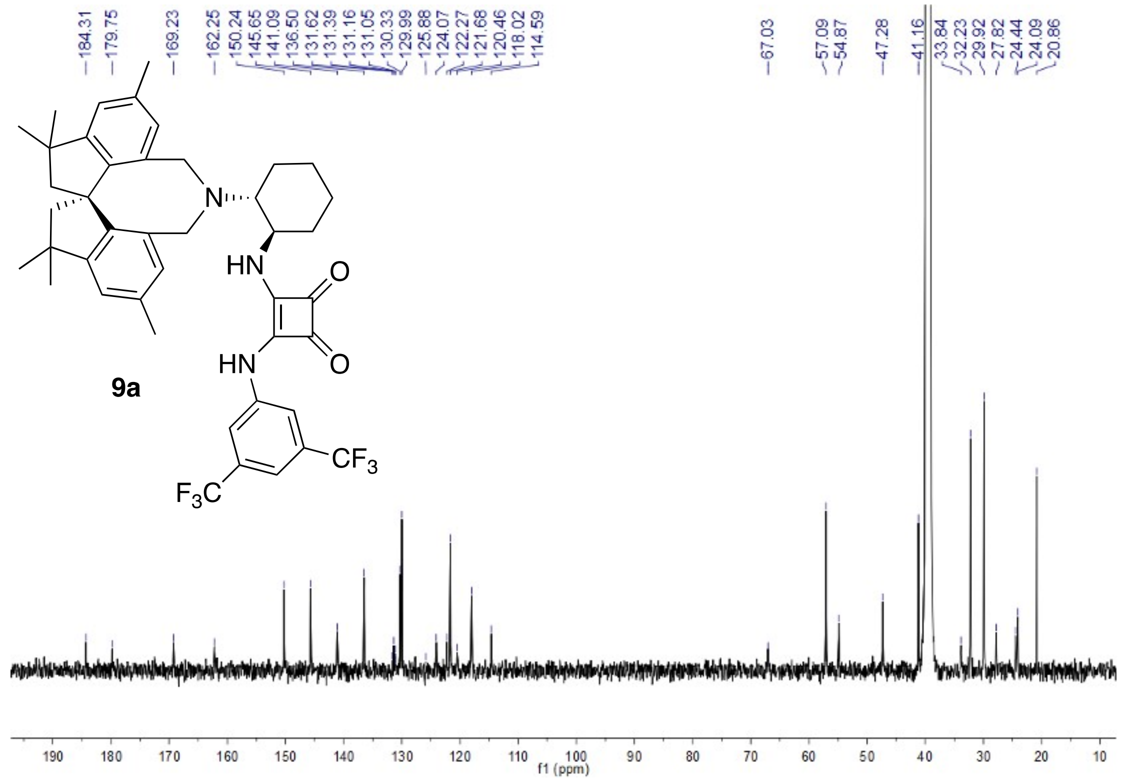

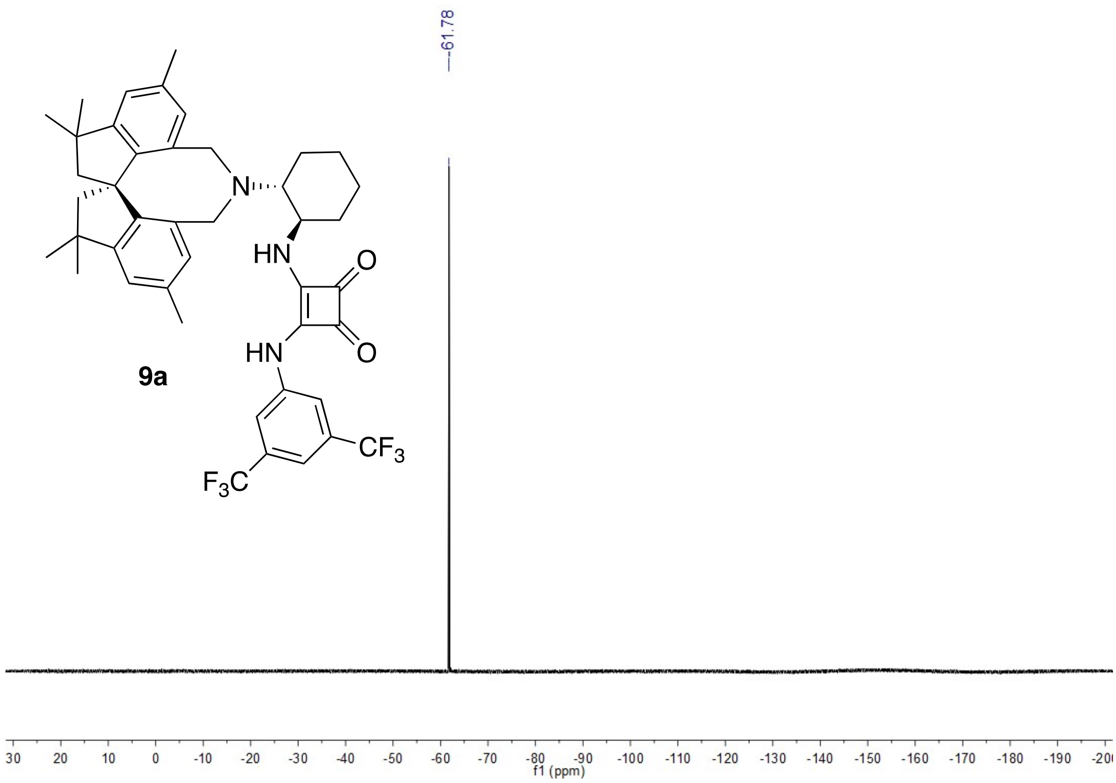


**Tert-butyl 2-oxo-3-(2,2,2-trifluoro-1-(5-hydroxy-1,3-diphenyl-1H-pyrazol-4-yl)ethyl)indoline-1-carboxylate (12a)**

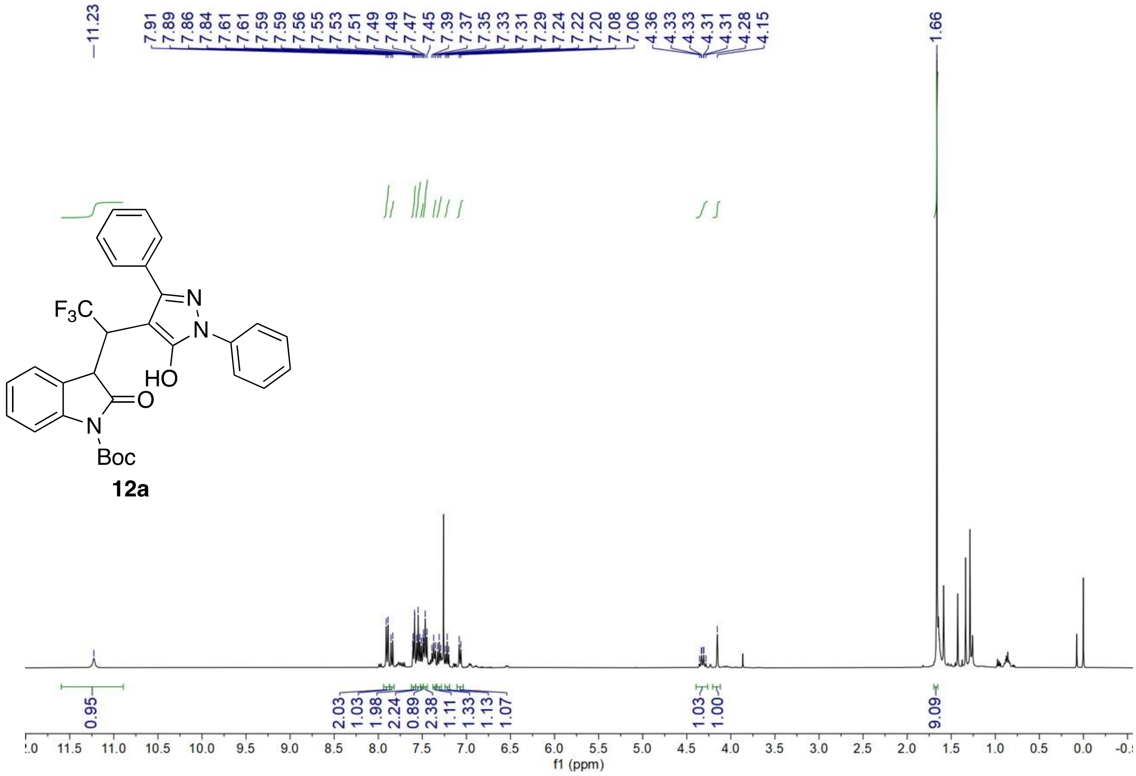

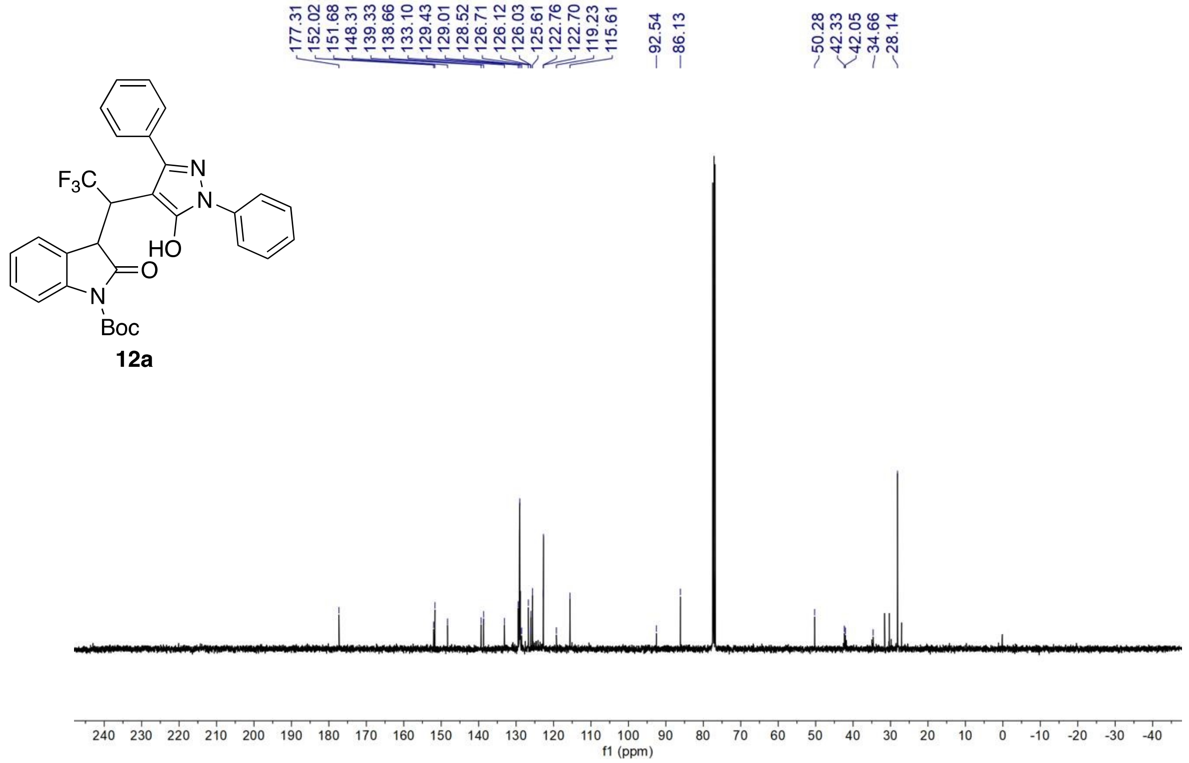
177.31
152.02
151.68
148.31
139.33
138.66
133.10
129.43
129.01
128.52
126.71
126.12
126.03
125.61
122.76
122.70
119.23
115.61
92.54
86.13
50.28
42.33
42.05
34.66
28.14
$F_3C$
N
N
HO
O
N
Boc
12a
240 230 220 210 200 190 180 170 160 150 140 130 120 110 100 90 80 70 60 50 40 30 20 10 0 -10 -20 -30 -40
f1 (ppm)

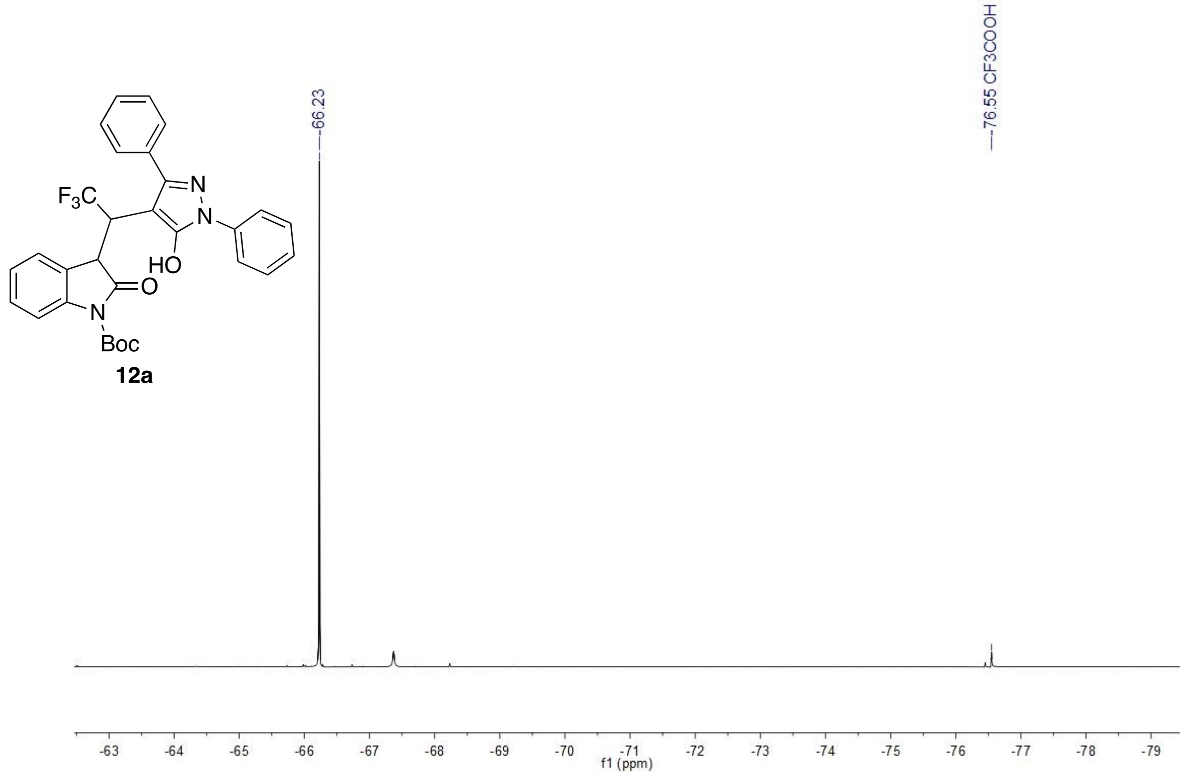
-66.23
-76.55 CF3COOH
$F_3C$
N
N
HO
O
N
Boc
12a
-63 -64 -65 -66 -67 -68 -69 -70 -71 -72 -73 -74 -75 -76 -77 -78 -79
f1 (ppm)

**(*R*)-3-((*R*)-2,2,2-trifluoro-1-(5-hydroxy-1,3-diphenyl-1*H*-pyrazol-4-yl)ethyl)indolin-2-one (13a).**

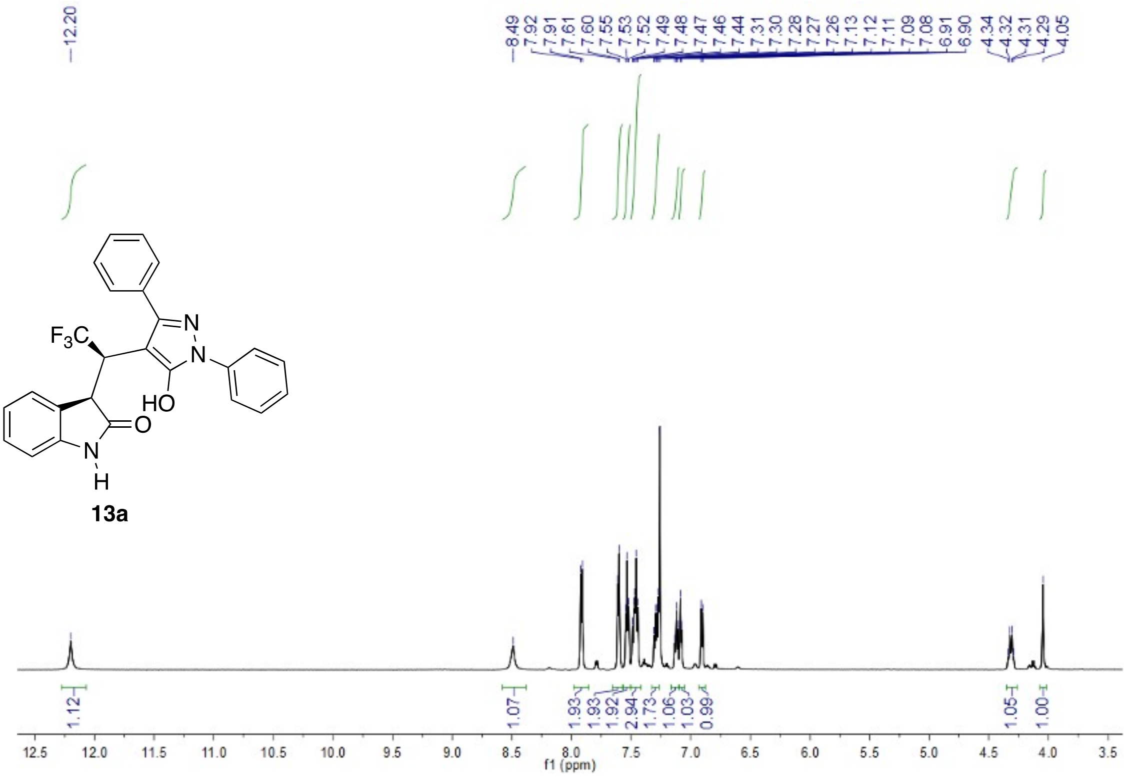

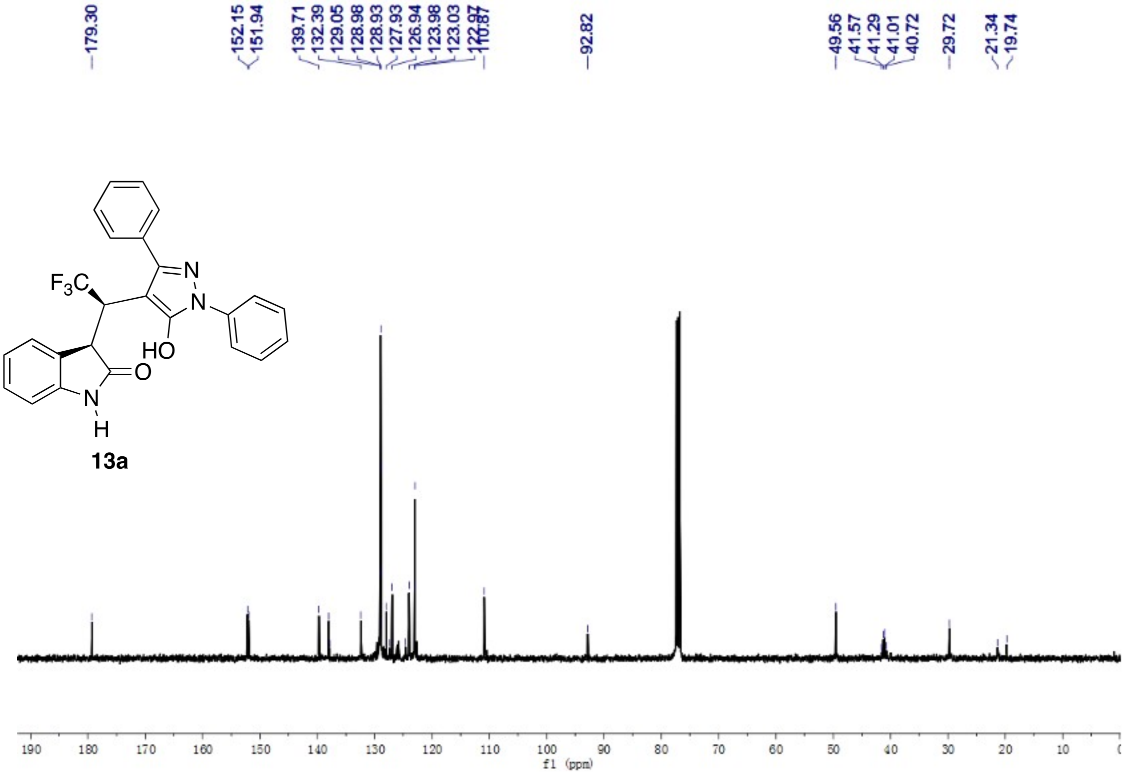

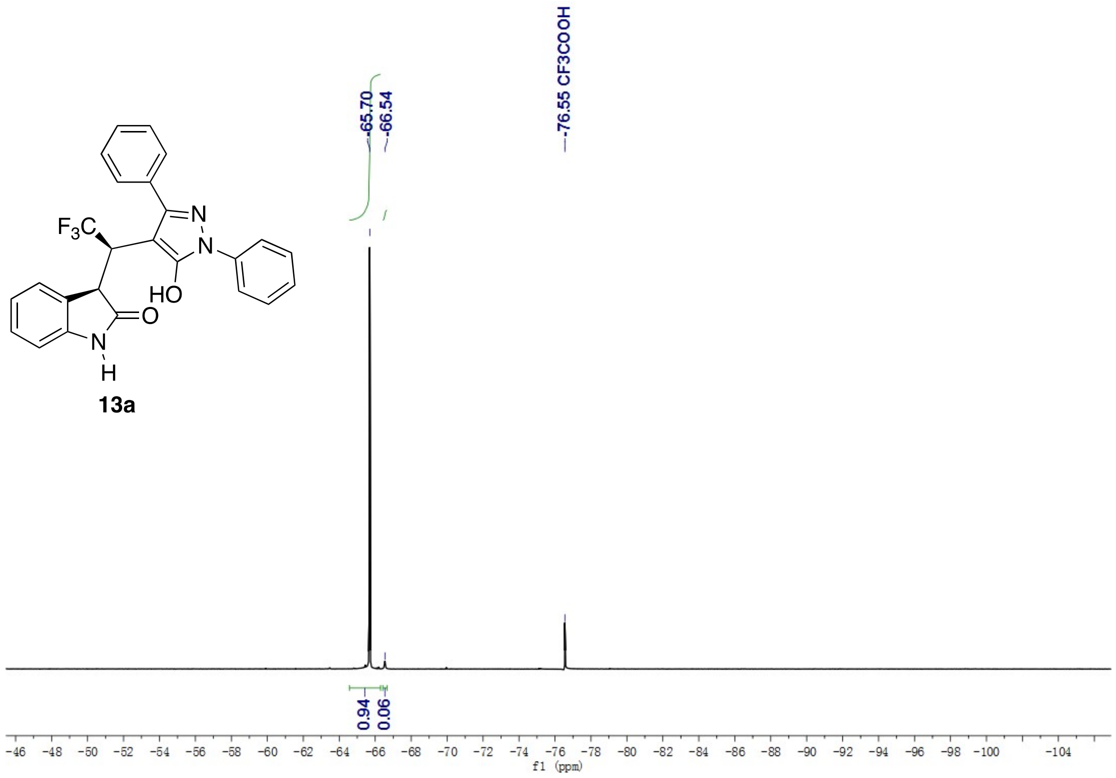


**(*R*)-3-((*R*)-1-(3-(2-chlorophenyl)-5-hydroxy-1-phenyl-1*H*-pyrazol-4-yl)-2,2,2-trifluoroethyl)indolin-2-one (13b)**

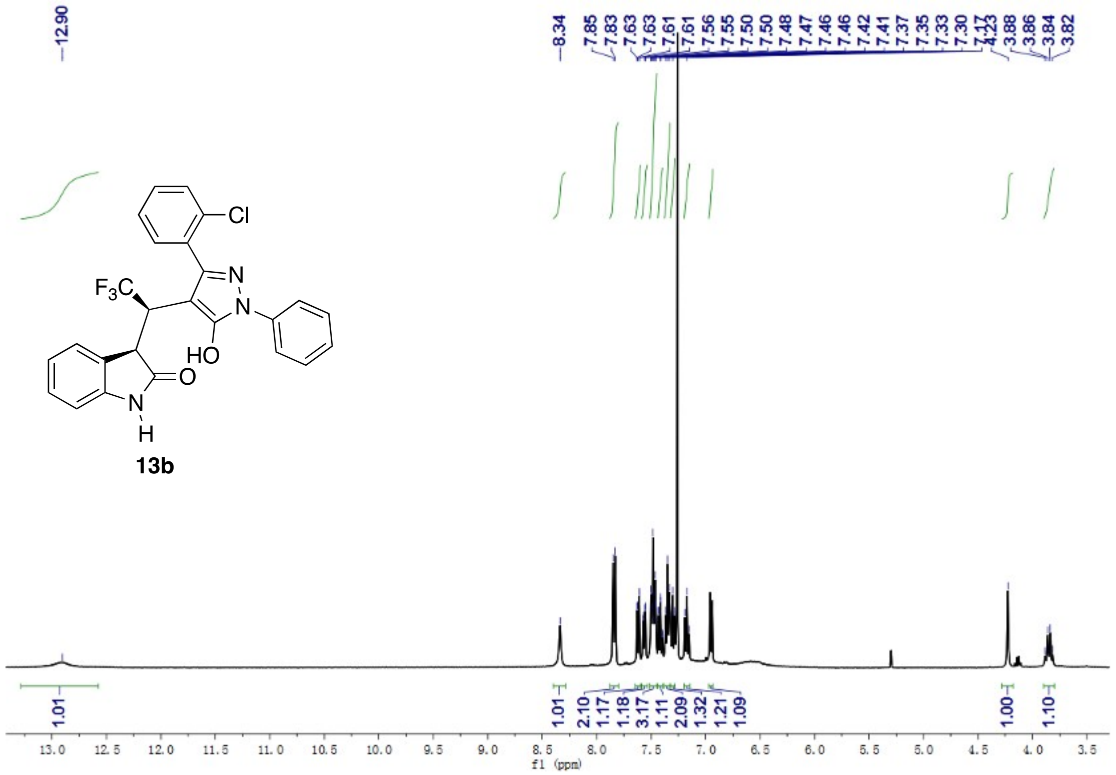

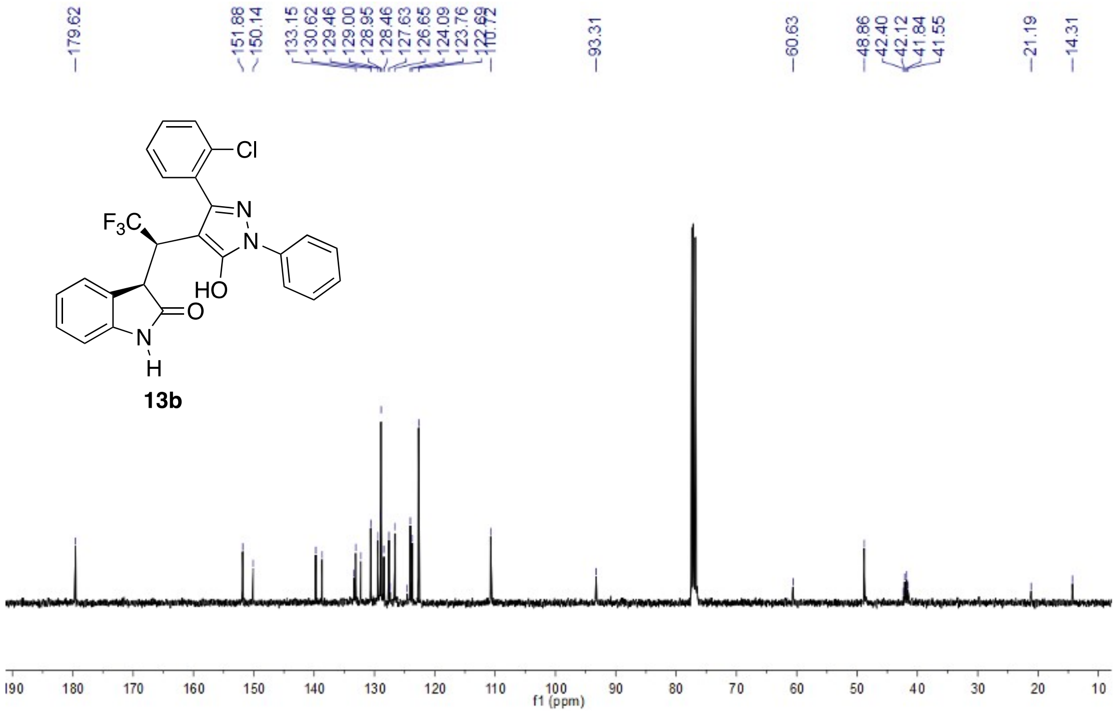
179.62
151.88
150.14
133.15
130.62
129.46
129.00
128.95
128.46
127.63
126.65
124.09
123.76
122.69
110.72
93.31
60.63
48.86
42.40
42.12
41.84
41.55
21.19
14.31
Cl
N
$F_3C$
N
HO
O
N
H
13b
190 180 170 160 150 140 130 120 110 100 90 80 70 60 50 40 30 20 10
f1 (ppm)

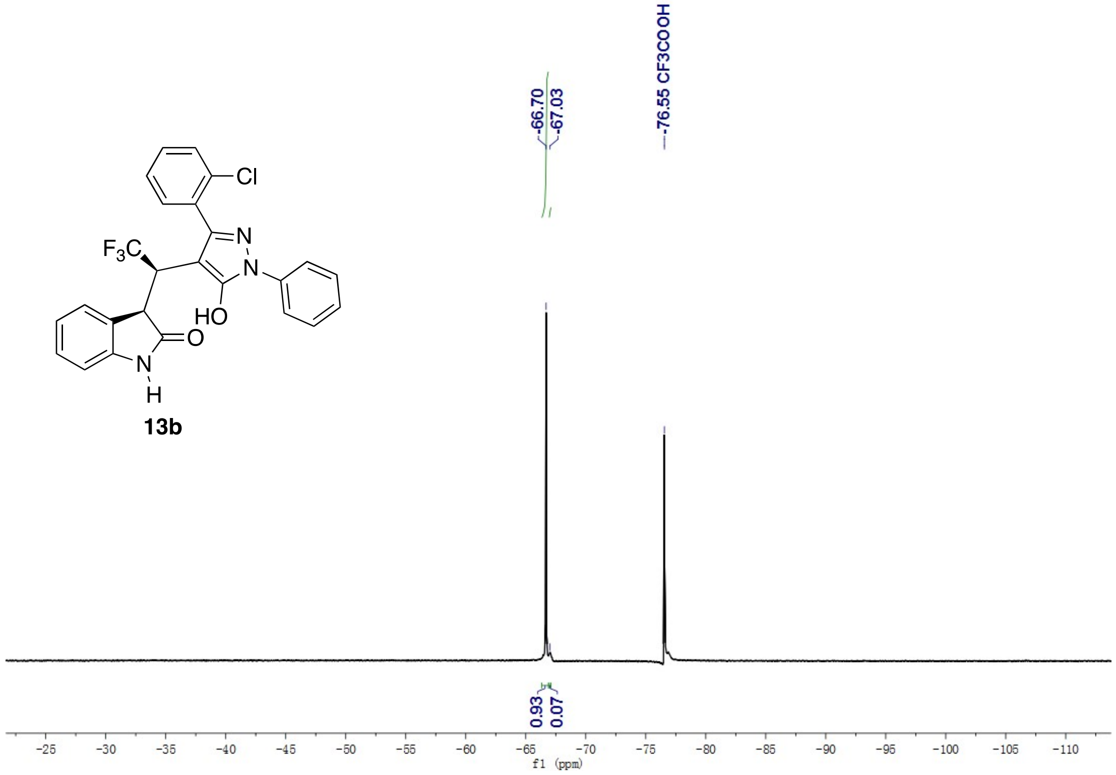
-66.70
-67.03
-76.55 CF3COOH
Cl
N
$F_3C$
N
HO
O
N
H
13b
0.93
0.07
-25 -30 -35 -40 -45 -50 -55 -60 -65 -70 -75 -80 -85 -90 -95 -100 -105 -110
f1 (ppm)

**(*R*)-3-((*R*)-1-(3-(3-chlorophenyl)-5-hydroxy-1-phenyl-1*H*-pyrazol-4-yl)-2,2,2-trifluoroethyl)indolin-2-one (13c)**

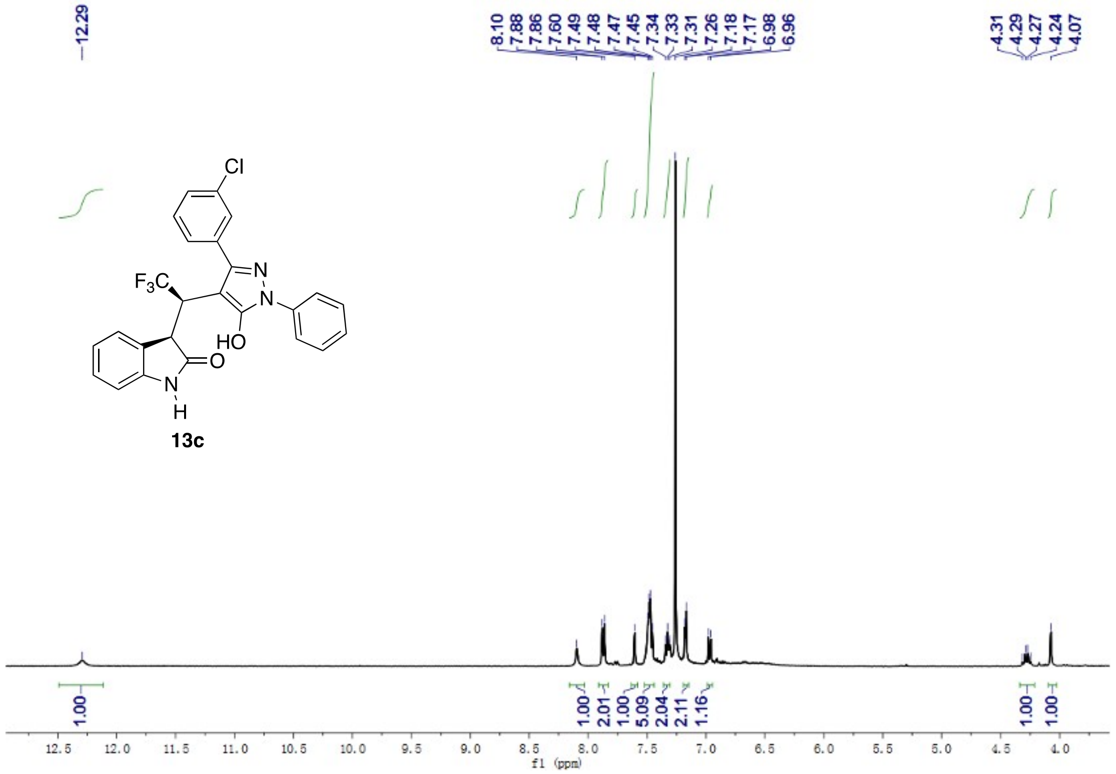


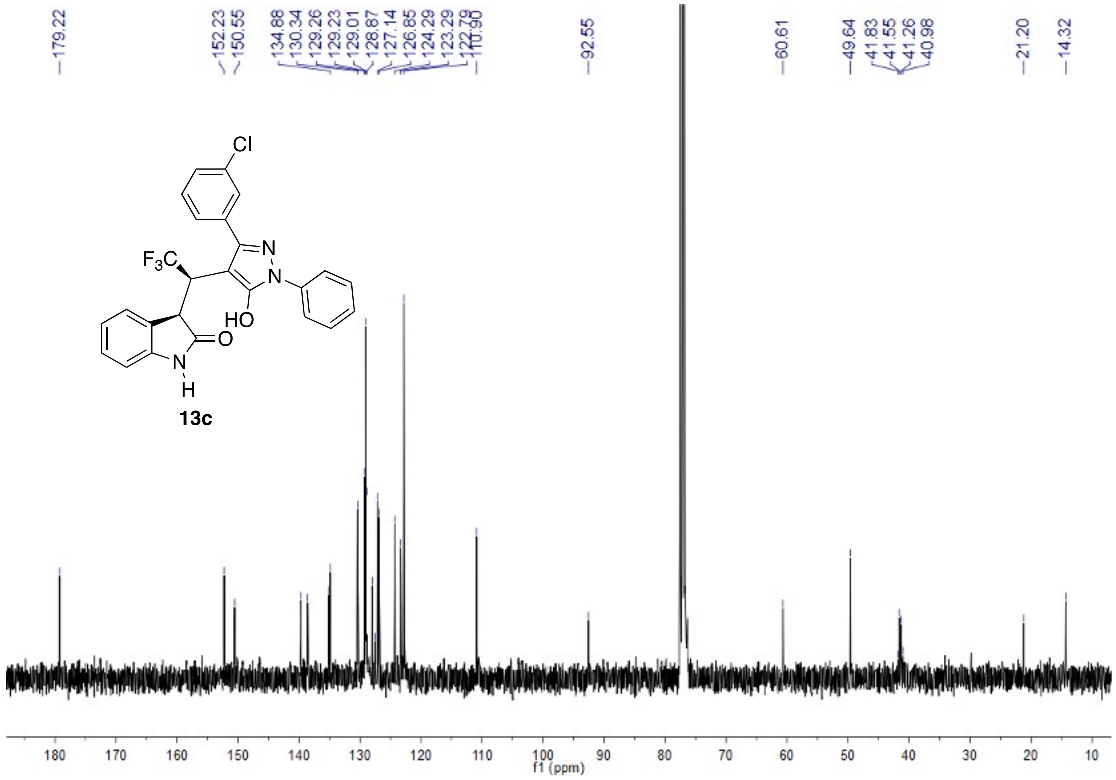

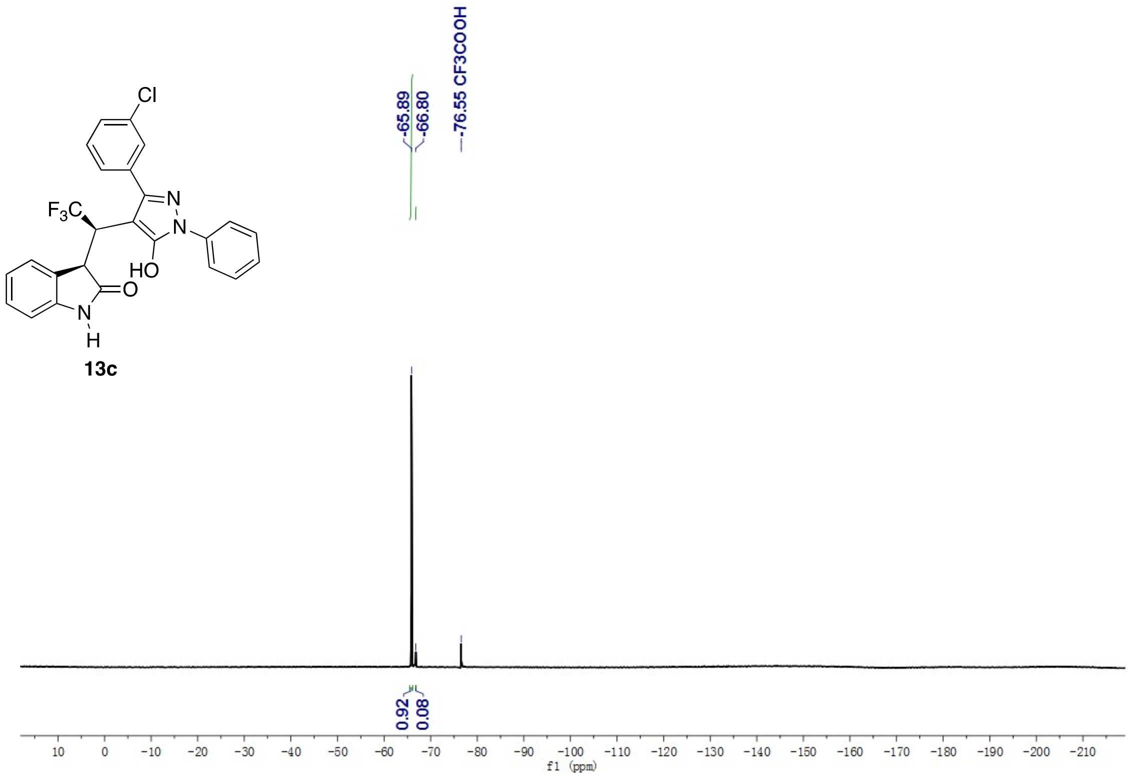


**(*R*)-3-((*R*)-1-(3-(4-chlorophenyl)-5-hydroxy-1-phenyl-1*H*-pyrazol-4-yl)-2,2,2-trifluoroethyl)indolin-2-one (13d)**

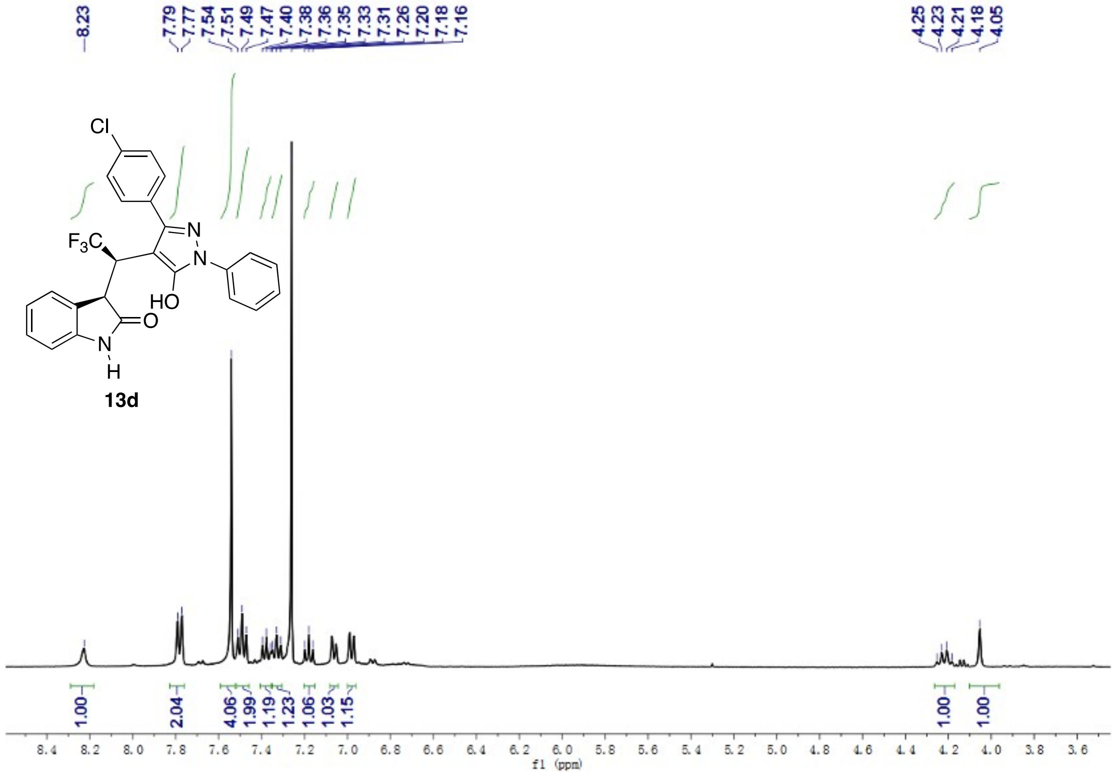

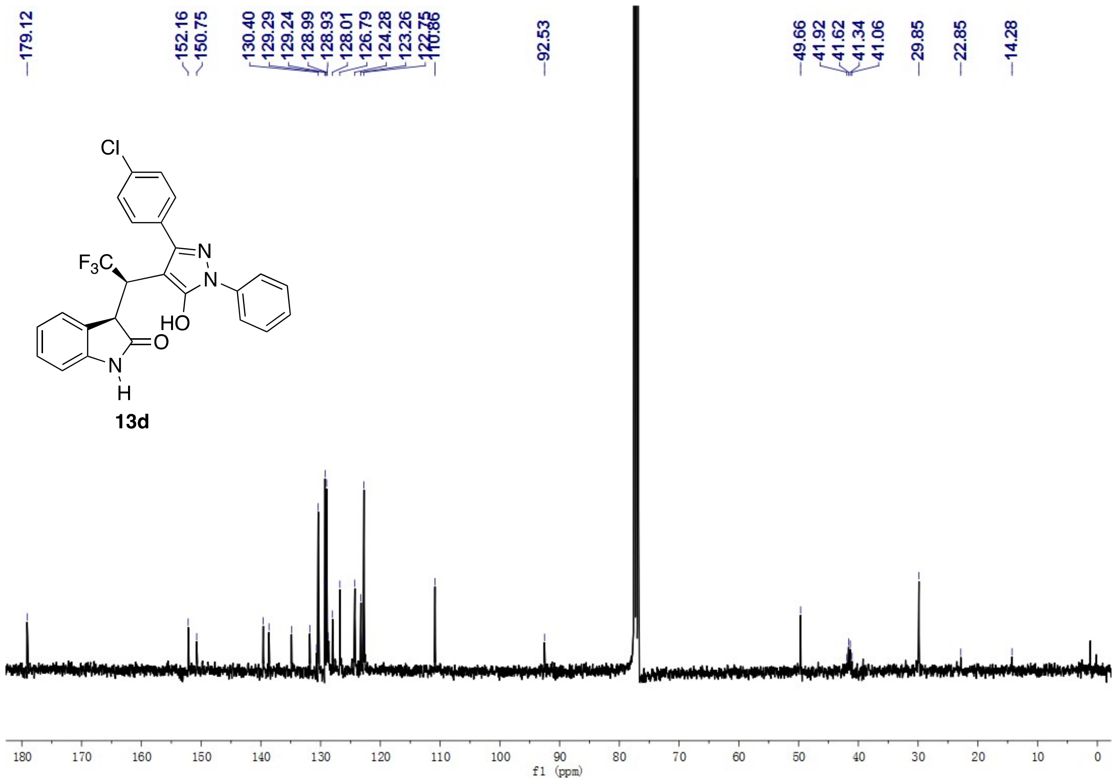

179.12
152.16
150.75
130.40
129.29
129.24
128.99
128.93
128.01
126.79
124.28
123.26
122.75
110.86
92.53
49.66
41.92
41.62
41.34
41.06
29.85
22.85
14.28
Cl
F3C
N
N
HO
O
N
H
13d
f1 (ppm)


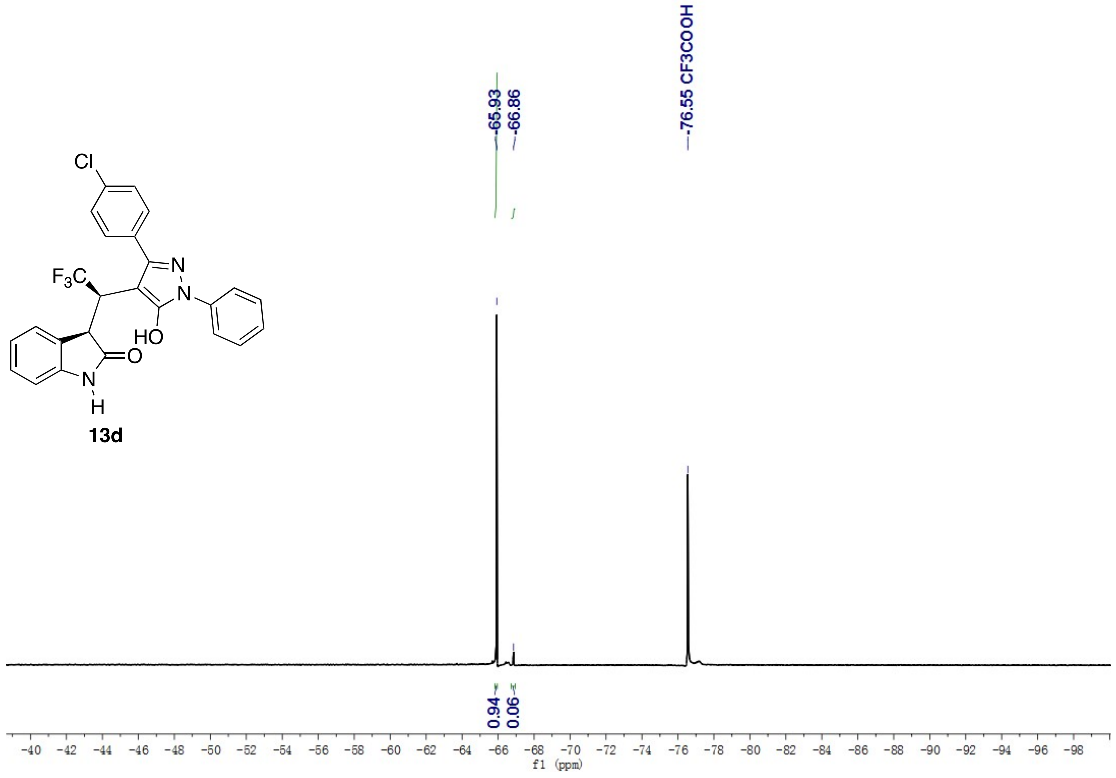

-65.93
-66.86
-76.55 CF3COOH
Cl
F3C
N
N
HO
O
N
H
13d
0.94
0.06
f1 (ppm)

**(*R*)-3-((*R*)-2,2,2-trifluoro-1-(5-hydroxy-1-phenyl-3-(*p*-tolyl)-1*H*-pyrazol-4-yl)ethyl)indolin-2-one (13e)**

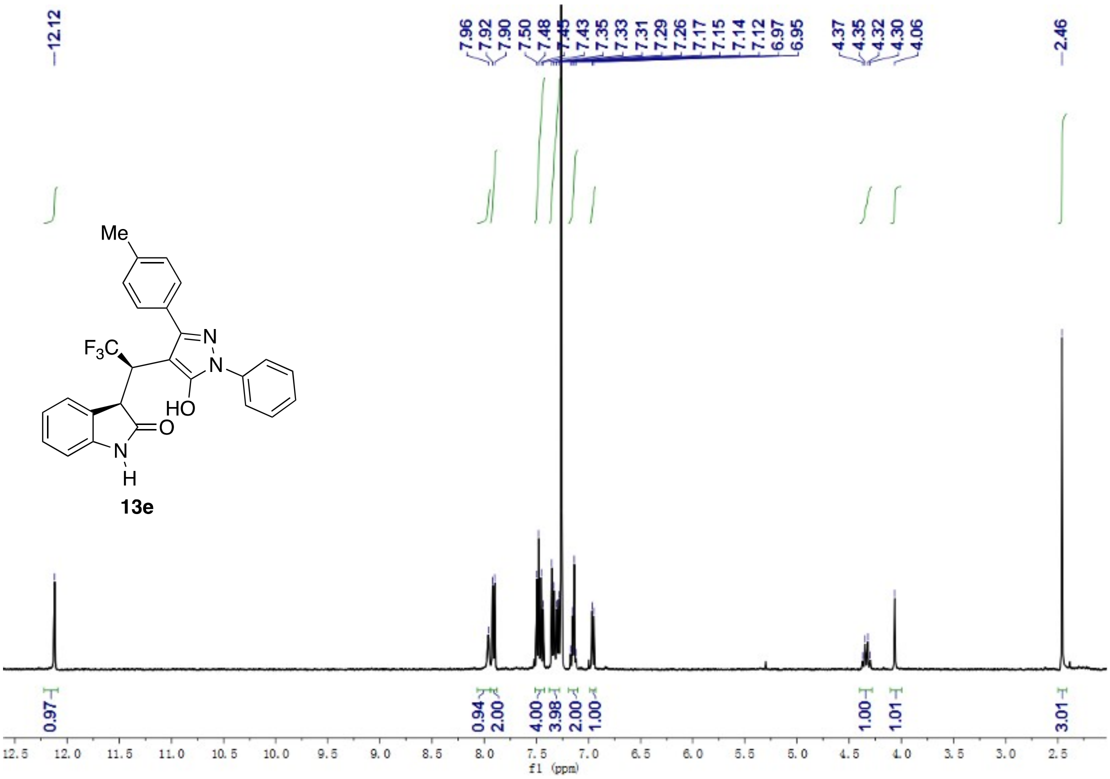


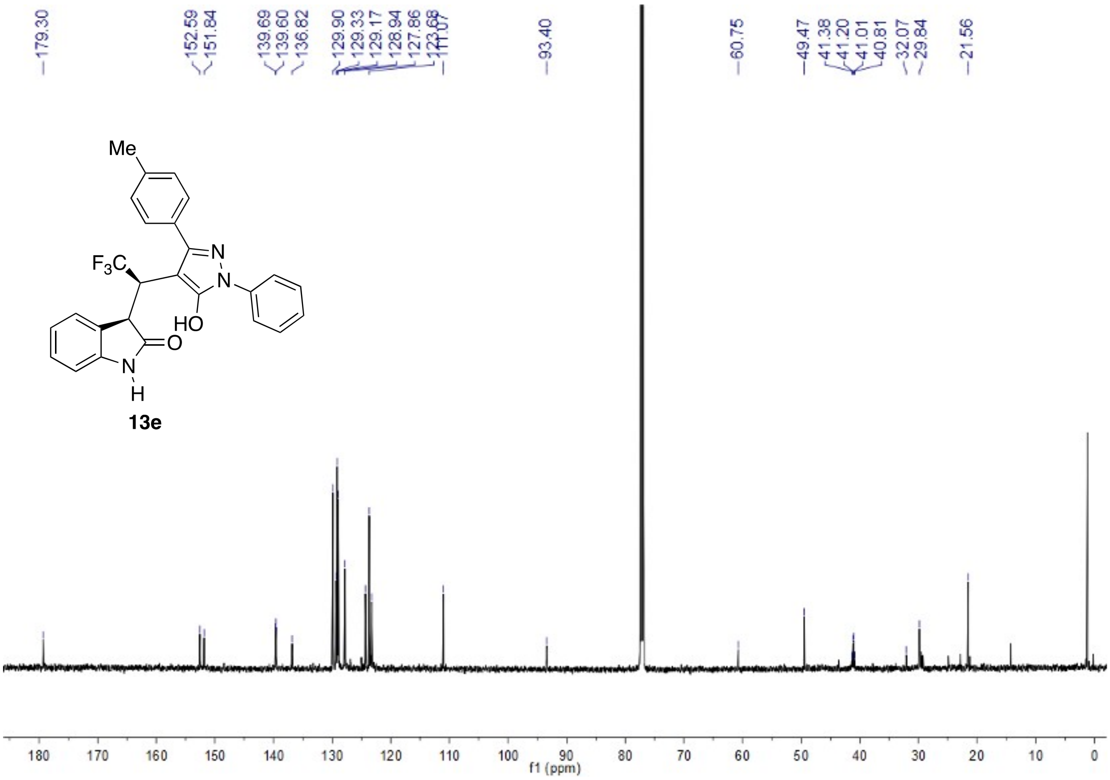

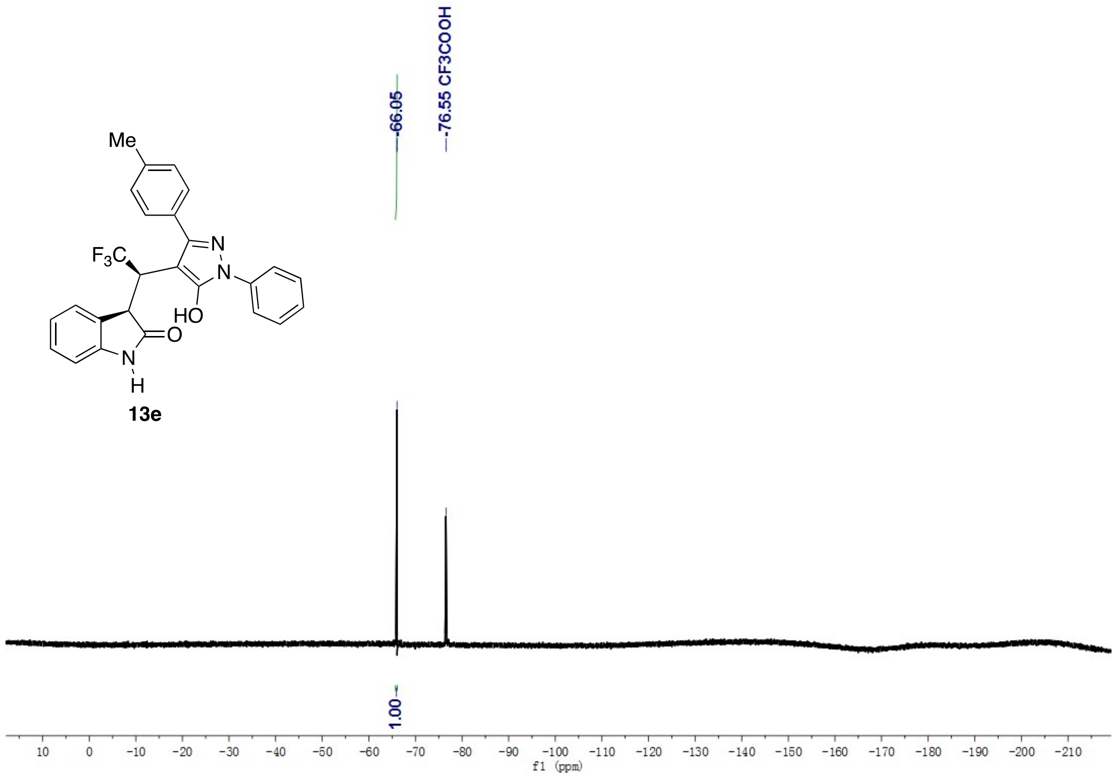


**(*R*)-3-((*R*)-2,2,2-trifluoro-1-(5-hydroxy-1-phenyl-3-(*m*-tolyl)-1*H*-pyrazol-4-yl)ethyl)indolin-2-one (13f)**

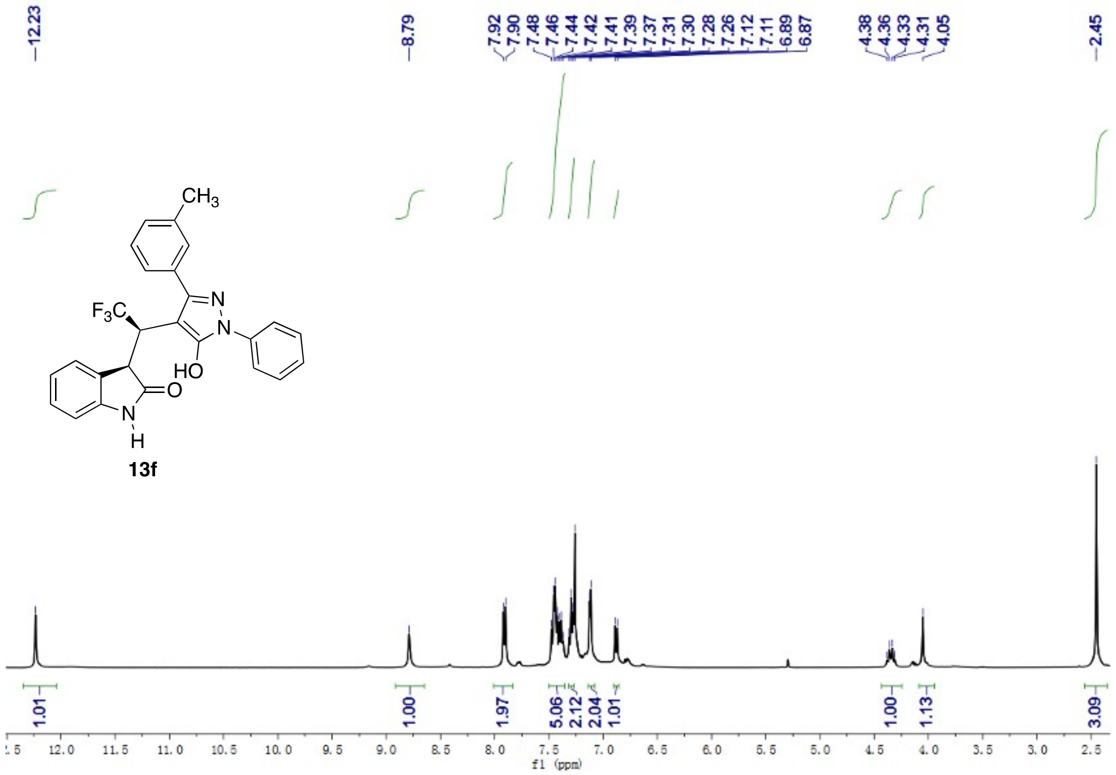

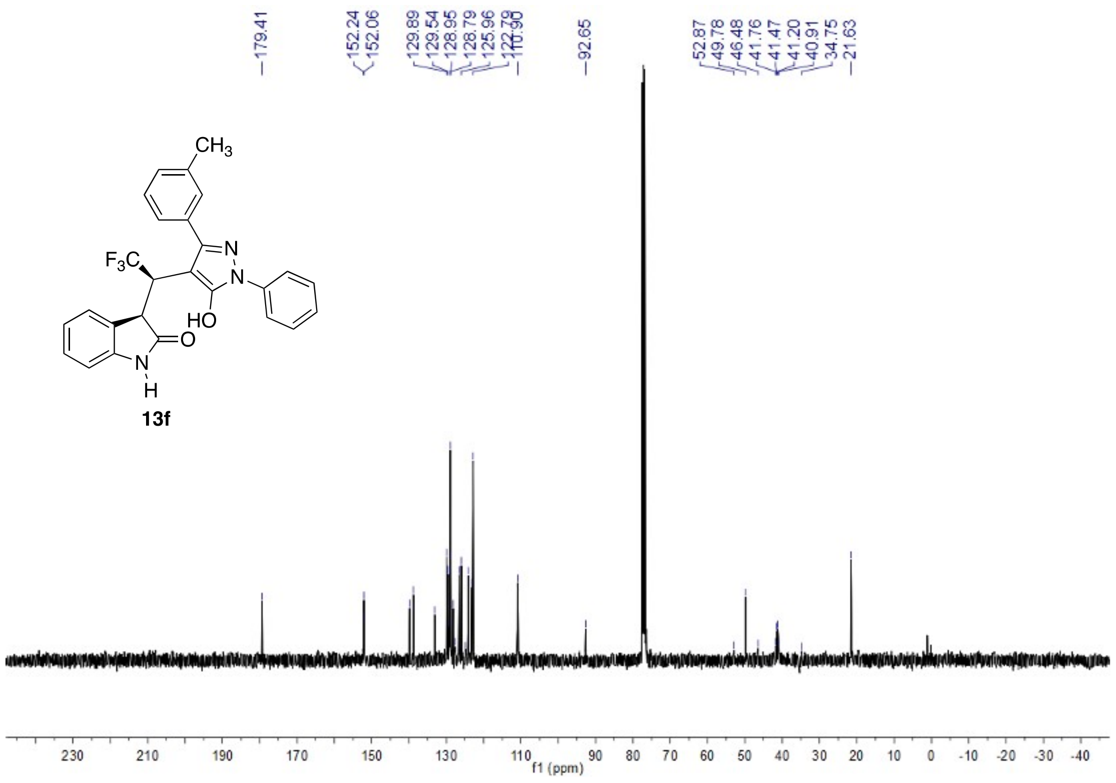

179.41
152.24
152.06
129.89
129.54
128.95
128.79
125.96
122.79
110.90
92.65
52.87
49.78
46.48
41.76
41.47
41.20
40.91
34.75
21.63
CH3
F3C
N
N
HO
O
N
H
13f
f1 (ppm)


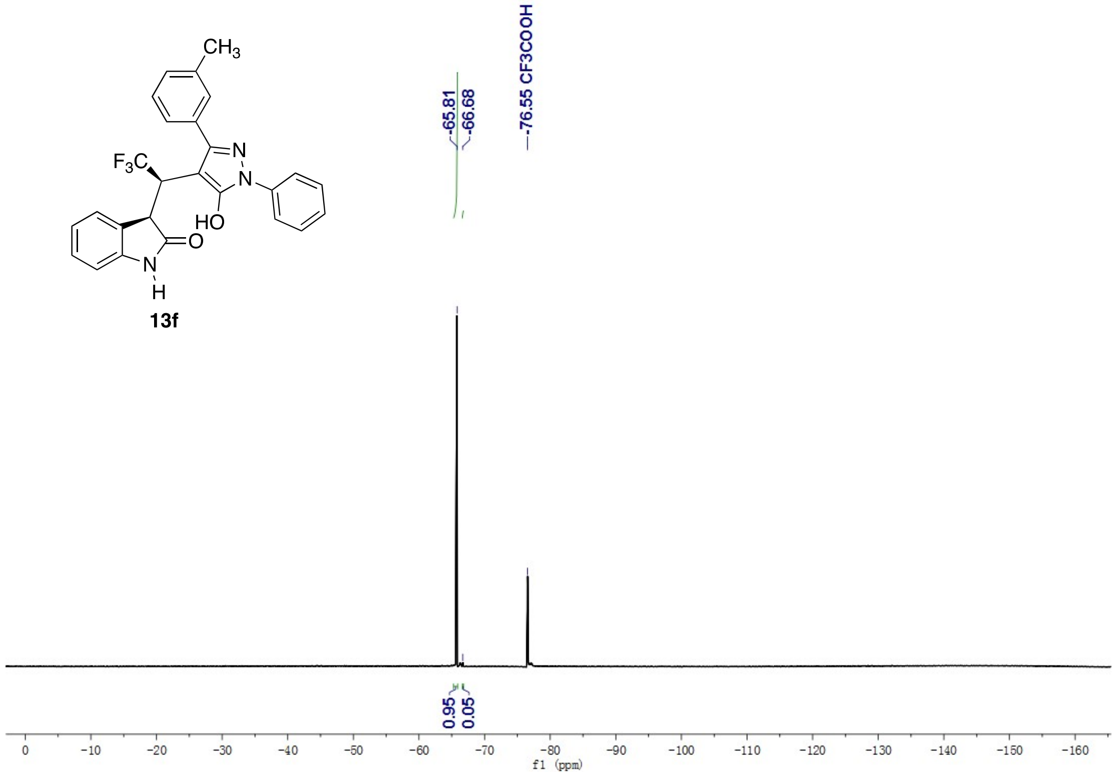

-65.81
-66.68
-76.55 CF3COOH
CH3
F3C
N
N
HO
O
N
H
13f
0.95
0.05
f1 (ppm)

**(*R*)-3-((*R*)-2,2,2-trifluoro-1-(5-hydroxy-1-(2-methoxyphenyl)-3-phenyl-1*H*-pyrazol-4-yl)ethyl)indolin-2-one (13g)**

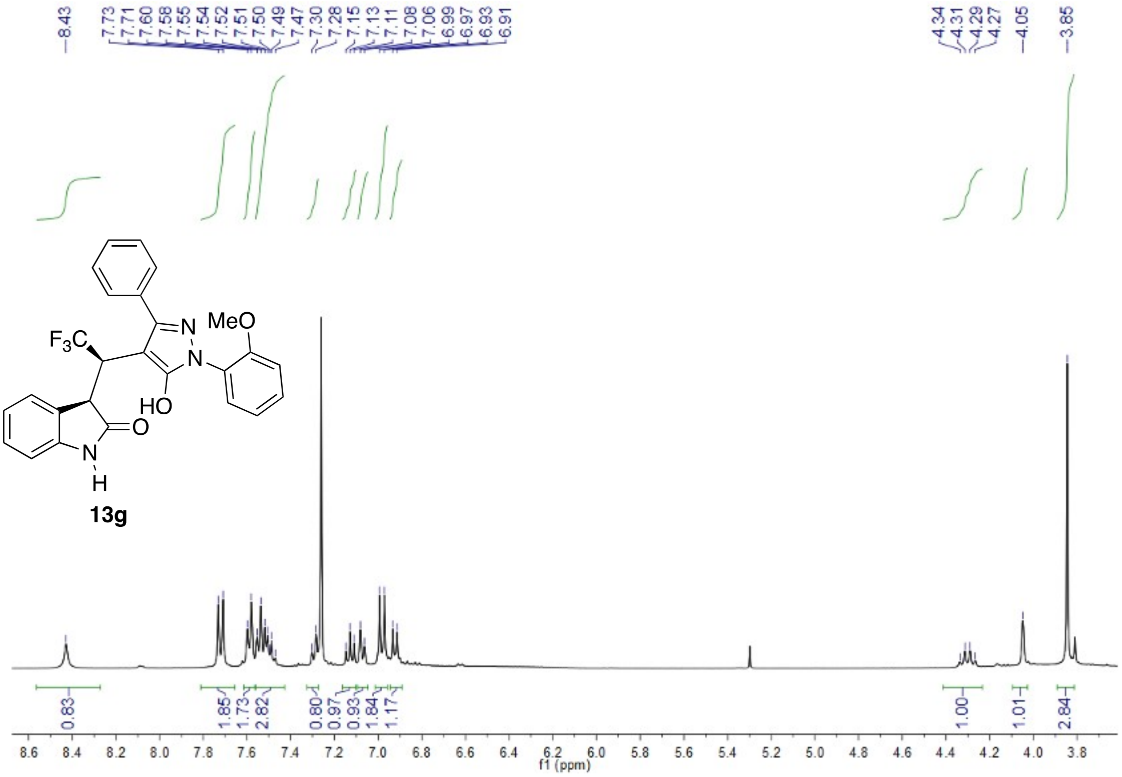


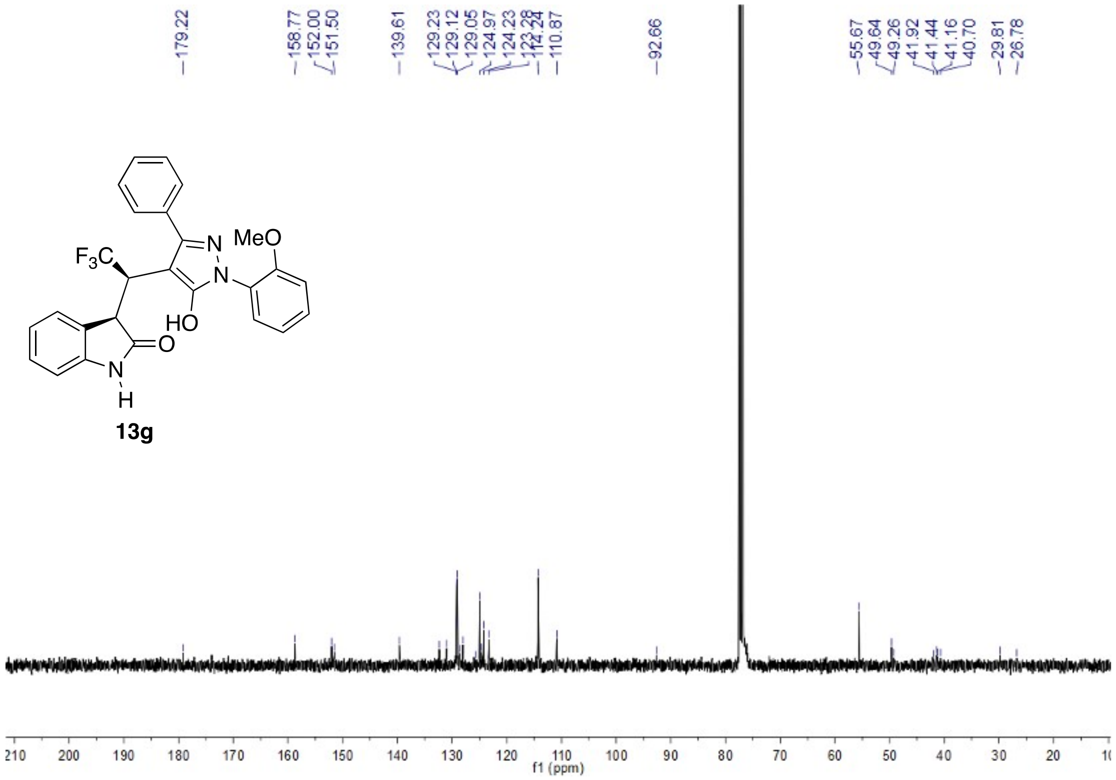

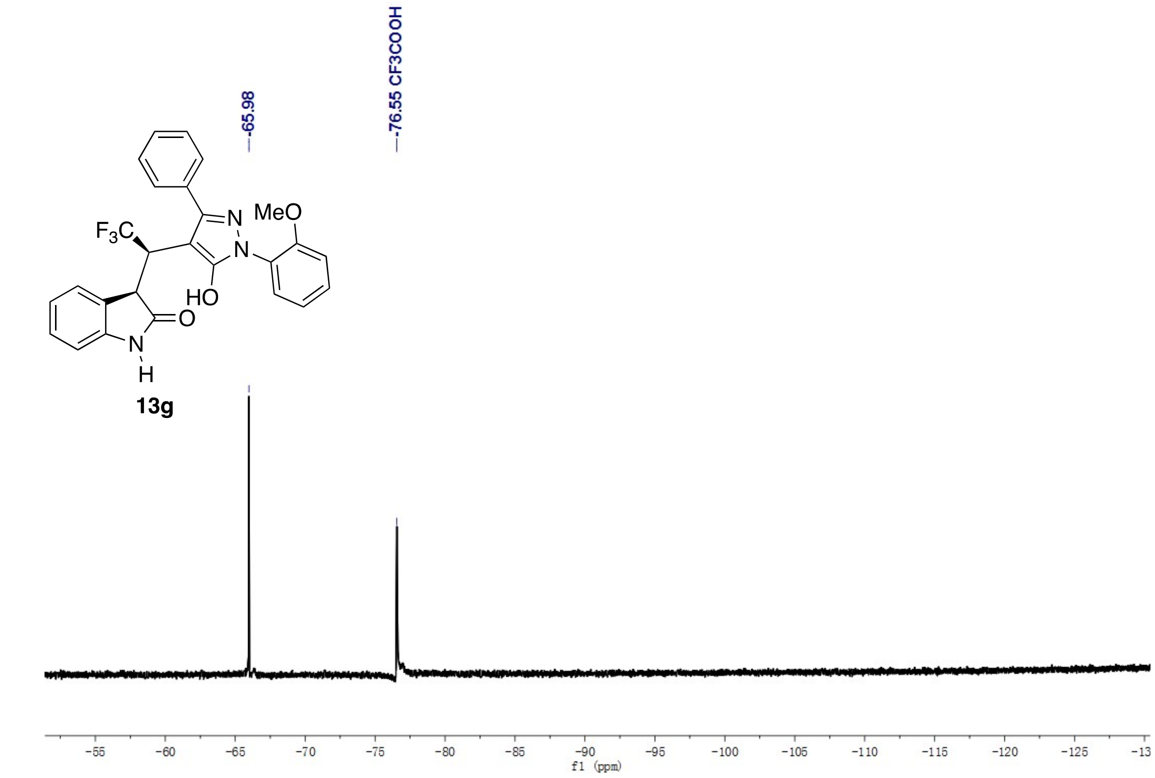


**(*R*)-3-((*R*)-2,2,2-trifluoro-1-(5-hydroxy-1-(4-methoxyphenyl)-3-phenyl-1*H*-pyrazol-4-yl)ethyl)indolin-2-one (13h)**

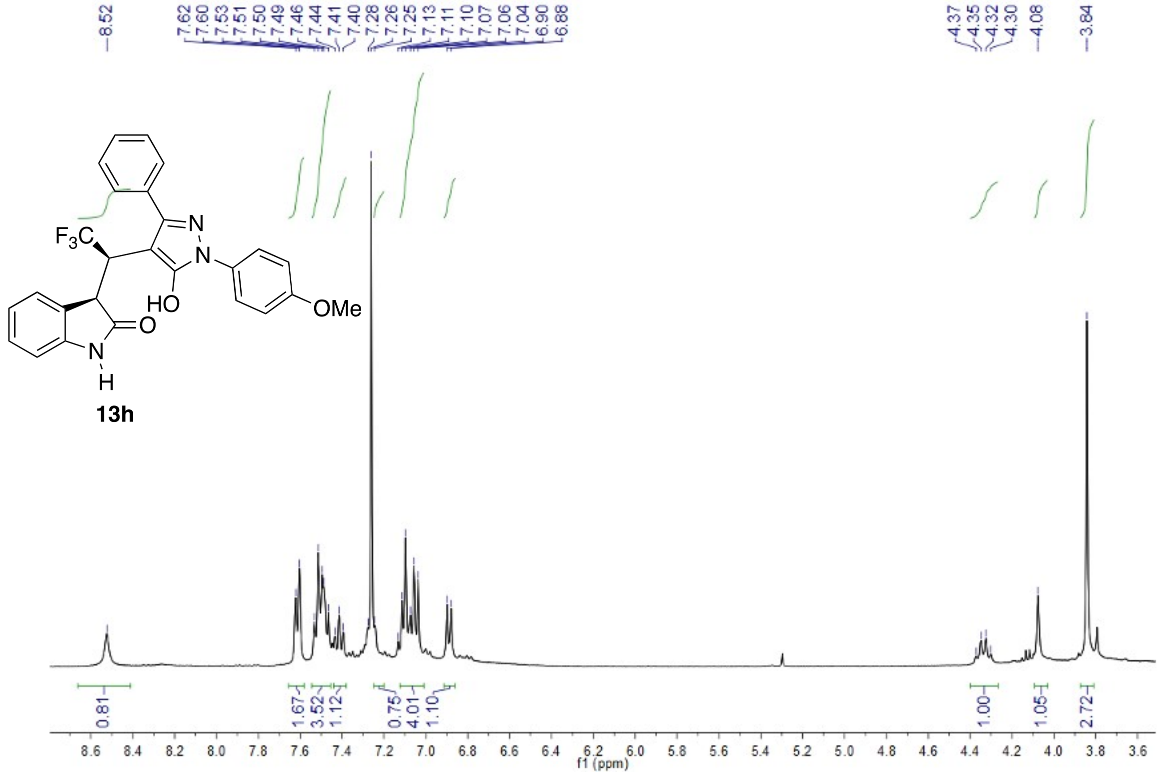

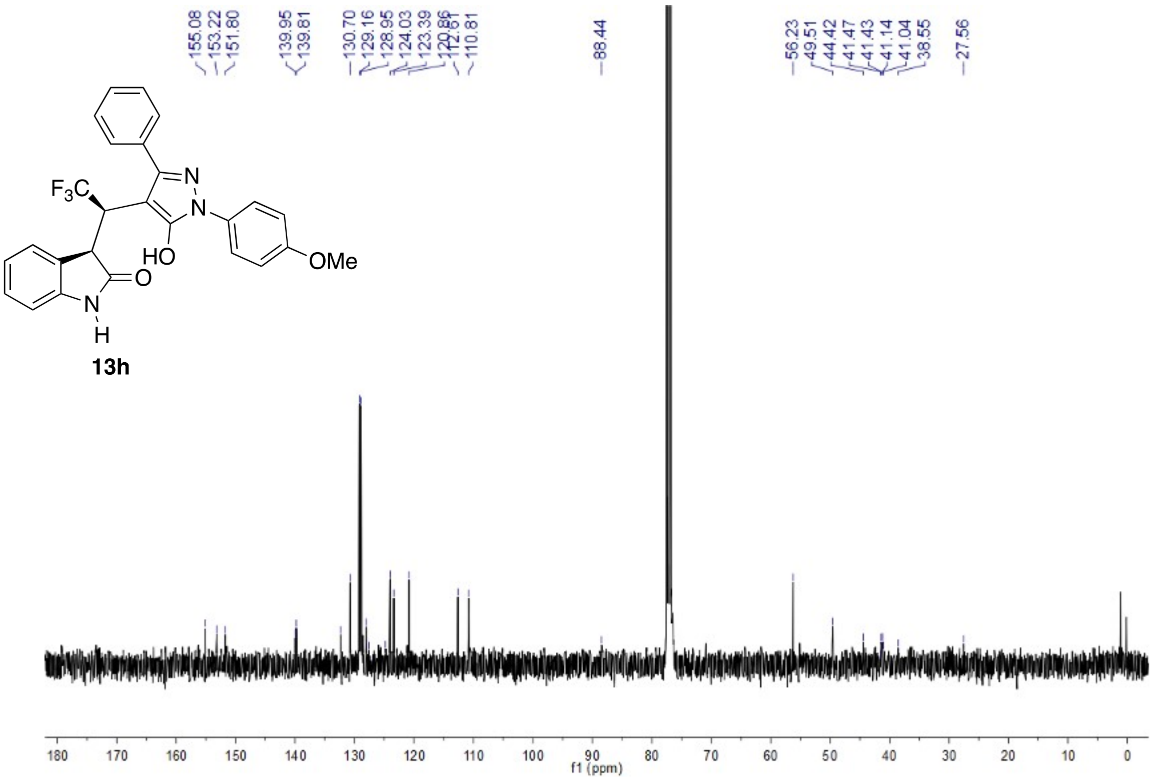
F3C
N
N
HO
OMe
O
N
H
13h
155.08
153.22
151.80
139.95
139.81
130.70
129.16
128.95
124.03
123.39
120.86
112.61
110.81
88.44
56.23
49.51
44.42
41.47
41.43
41.14
41.04
38.55
27.56
180 170 160 150 140 130 120 110 100 90 80 70 60 50 40 30 20 10 0
f1 (ppm)

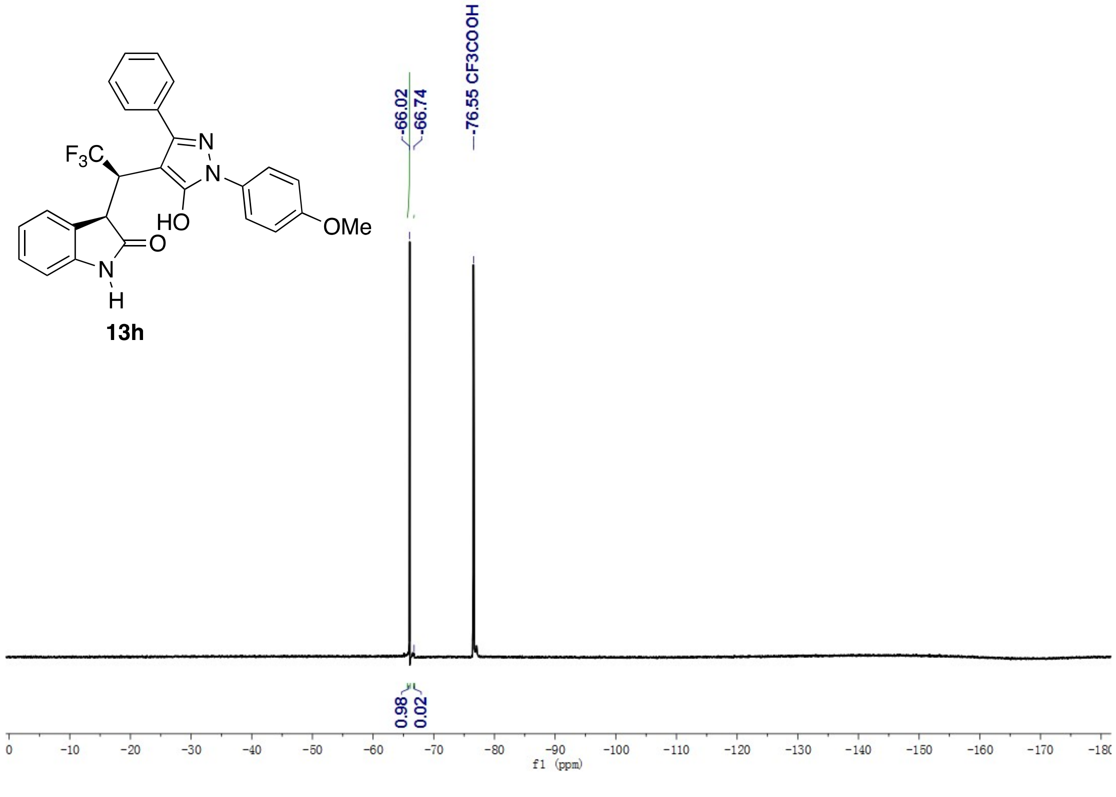
F3C
N
N
HO
OMe
O
N
H
13h
-66.02
-66.74
-76.55 CF3COOH
0.98
0.02
0 -10 -20 -30 -40 -50 -60 -70 -80 -90 -100 -110 -120 -130 -140 -150 -160 -170 -180
f1 (ppm)

**(*R*)-3-((*R*)-1-(1-(4-chlorophenyl)-5-hydroxy-3-phenyl-1*H*-pyrazol-4-yl)-2,2,2-trifluoroethyl)indolin-2-one (13i)**

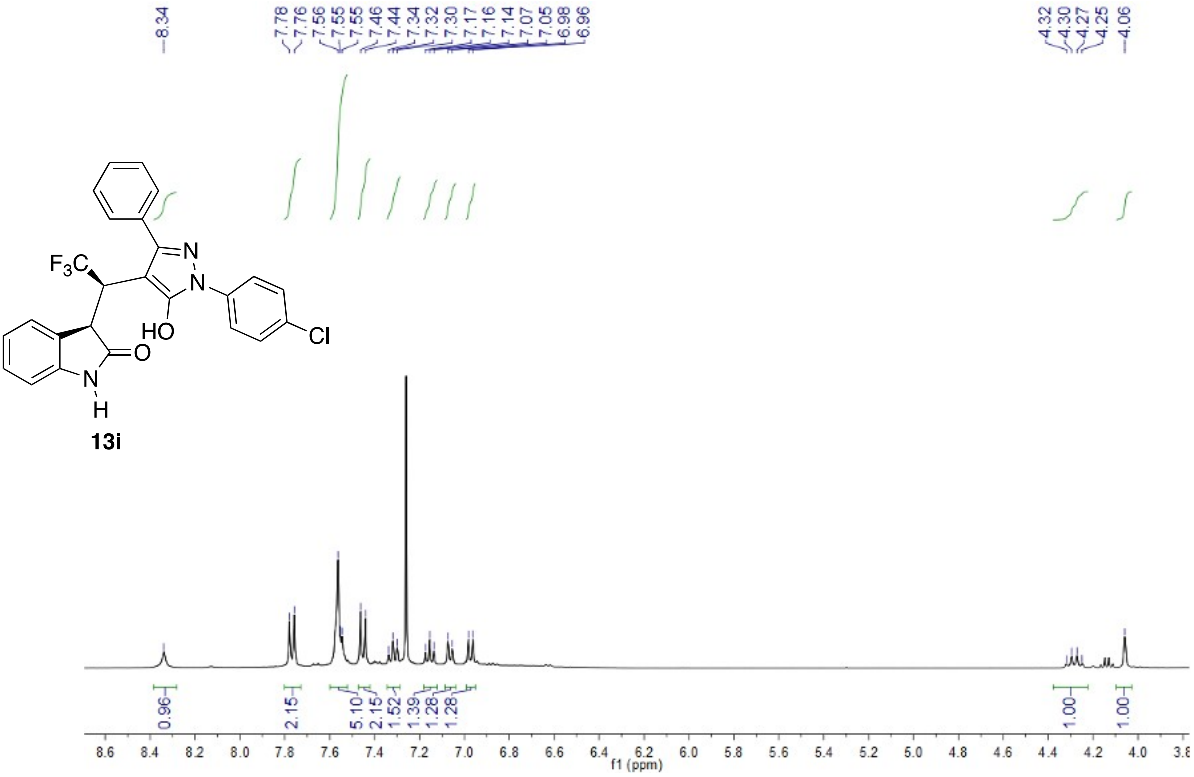


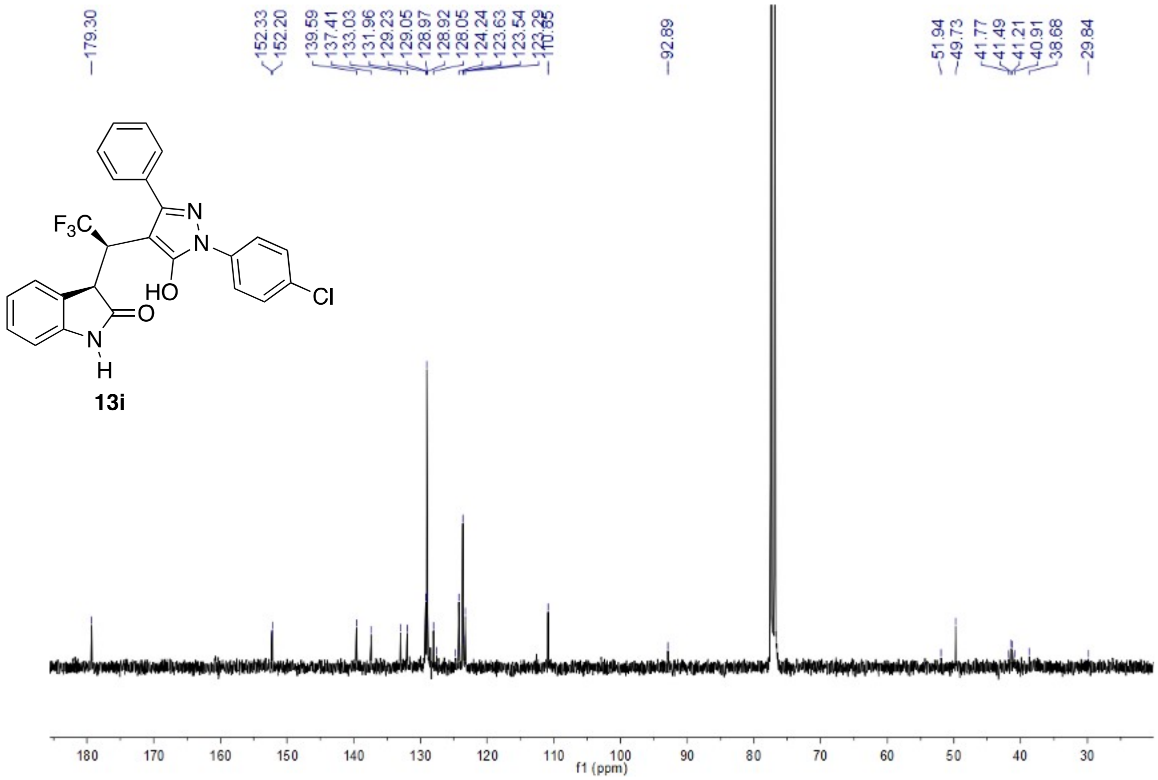

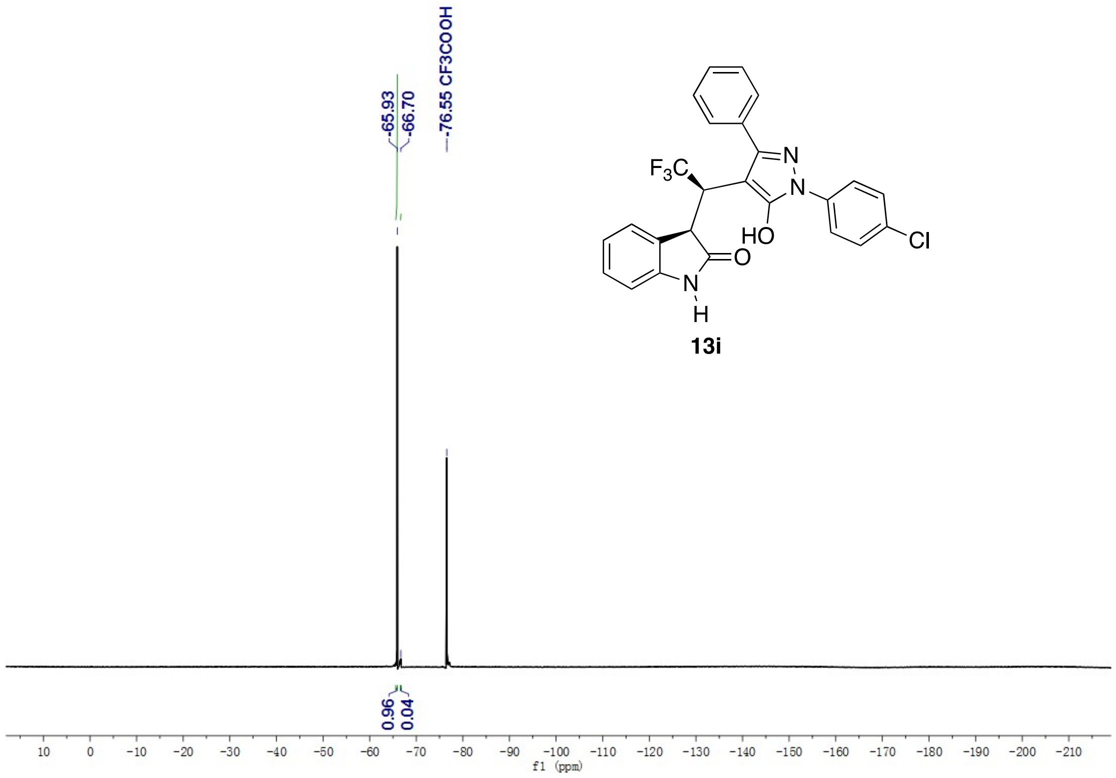


**(*R*)-3-((*R*)-1-(1-(3-chlorophenyl)-5-hydroxy-3-phenyl-1*H*-pyrazol-4-yl)-2,2,2-trifluoroethyl)indolin-2-one (13j)**

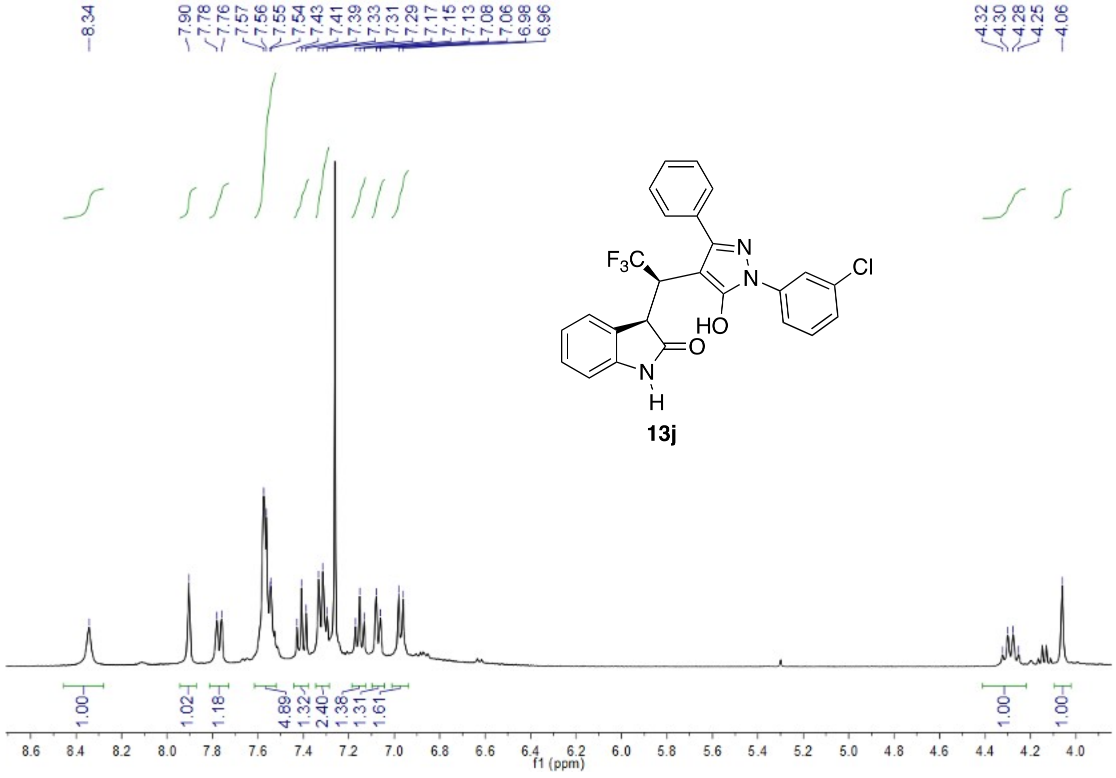

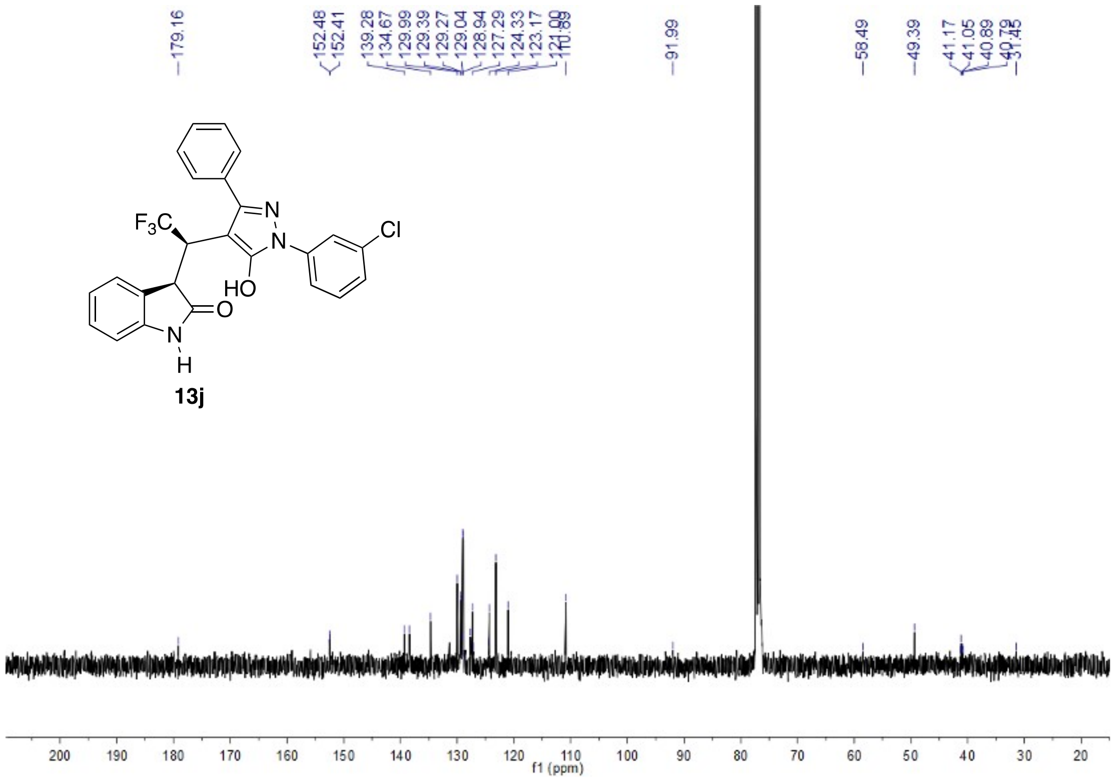
179.16
152.48
152.41
139.28
134.67
129.99
129.39
129.27
129.04
128.94
127.29
124.33
123.17
91.99
58.49
49.39
41.17
41.05
40.89
F3C
N
N
Cl
HO
O
N
H
13j
f1 (ppm)

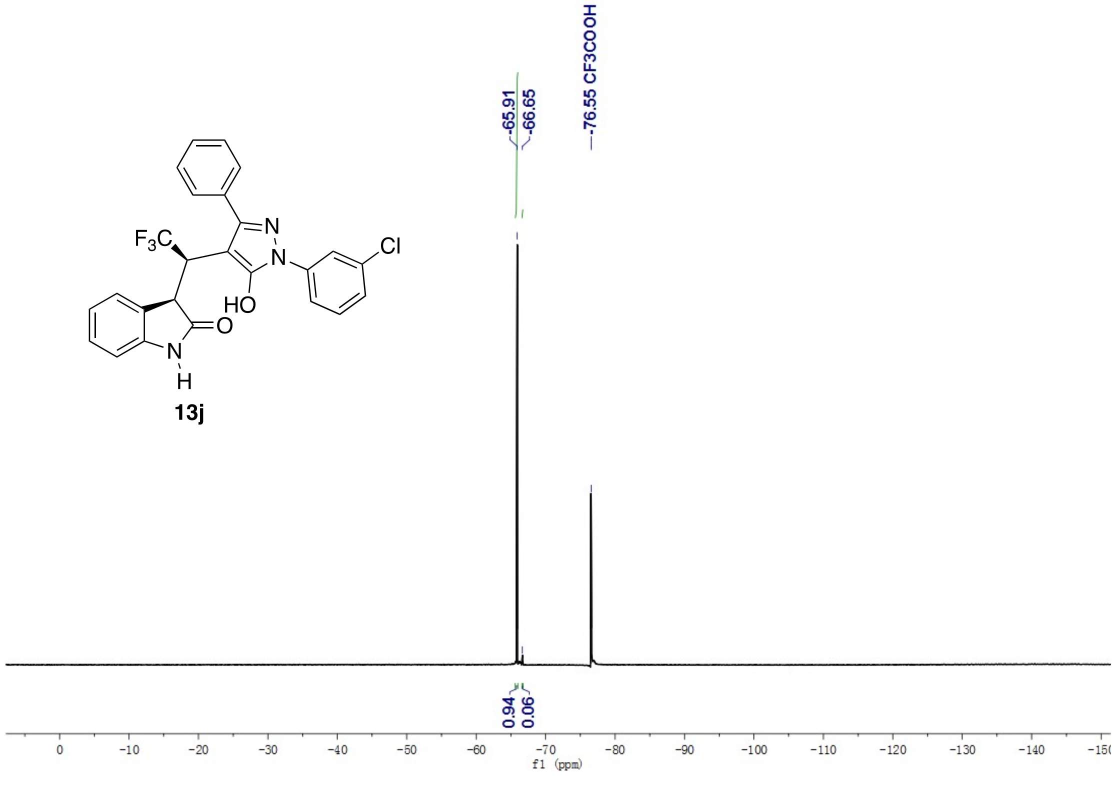
-65.91
-66.65
-76.55 CF3COOH
F3C
N
N
Cl
HO
O
N
H
13j
0.94
0.06
f1 (ppm)

**(*R*)-3-((*R*)-1-(1-(2-chlorophenyl)-5-hydroxy-3-phenyl-1*H*-pyrazol-4-yl)-2,2,2-trifluoroethyl)indolin-2-one (13k)**

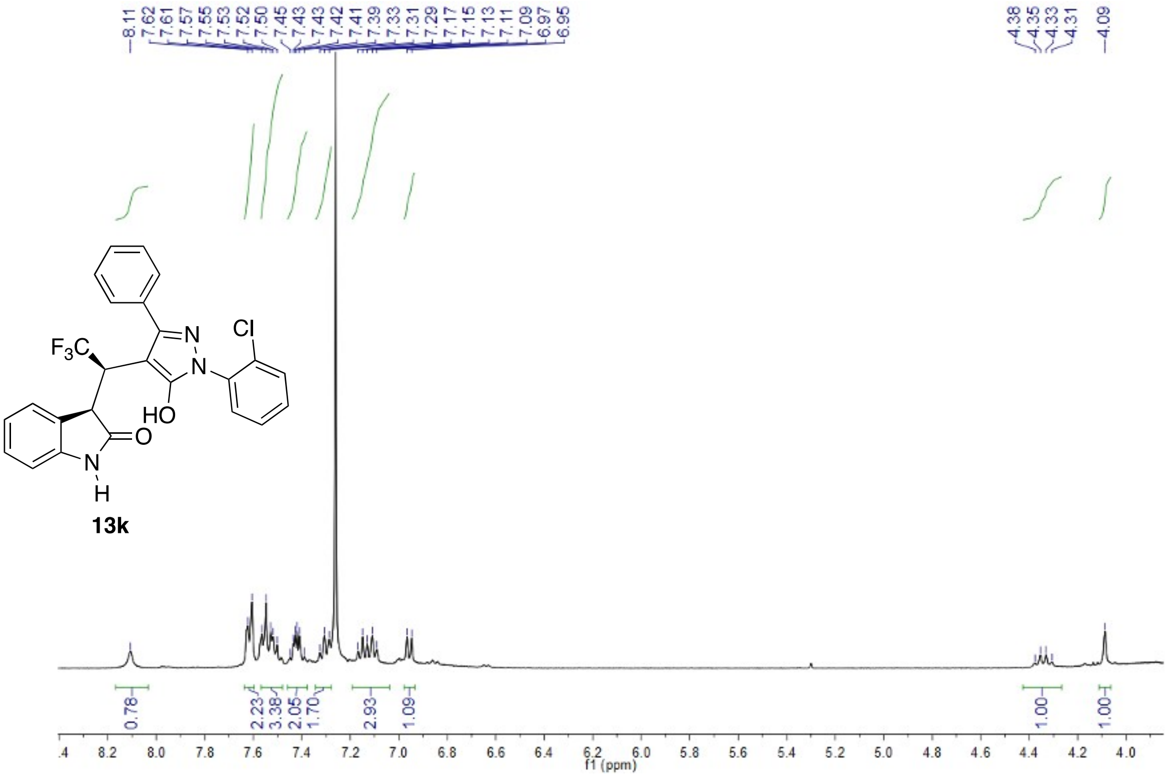


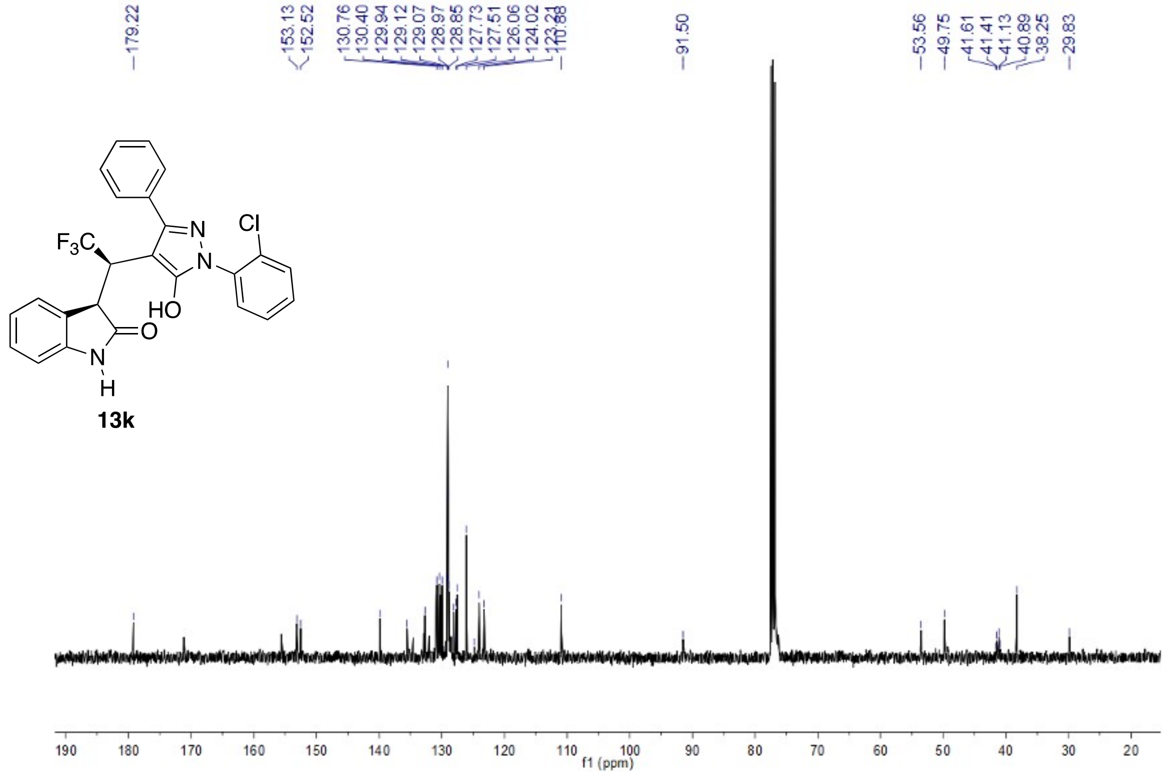

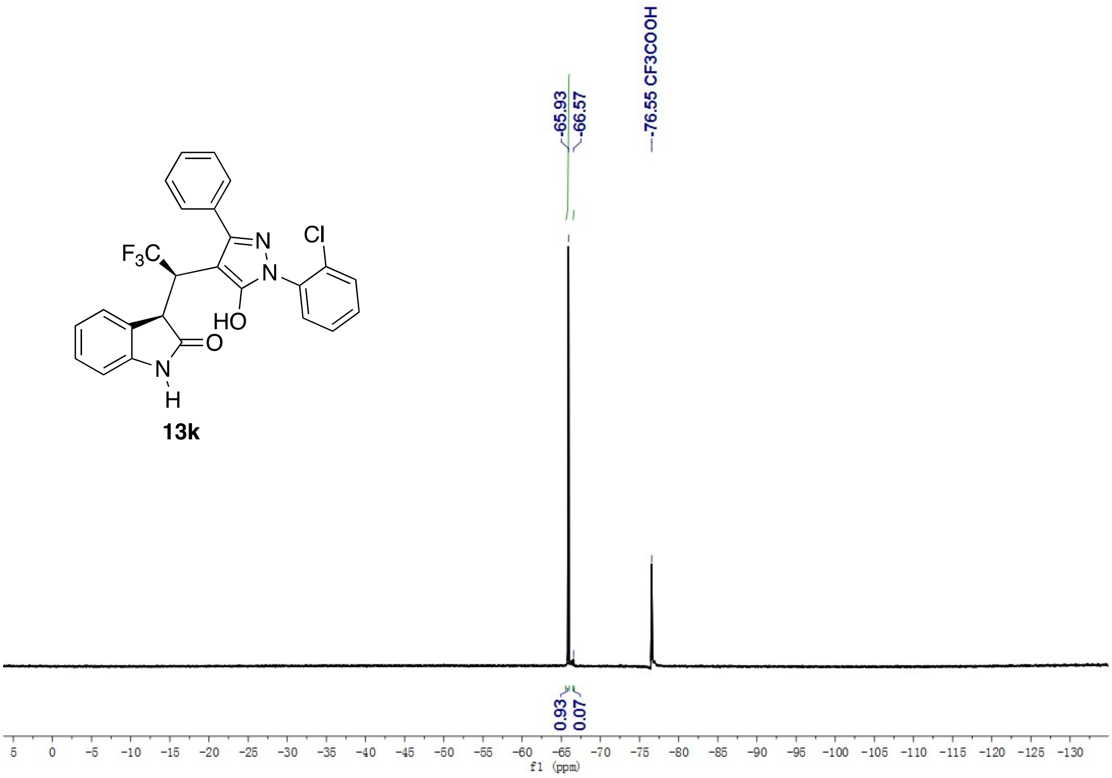


**(*R*)-4-bromo-3-((*R*)-2,2,2-trifluoro-1-(5-hydroxy-1,3-diphenyl-1*H*-pyrazol-4-yl)ethyl)indolin-2-one (13l)**

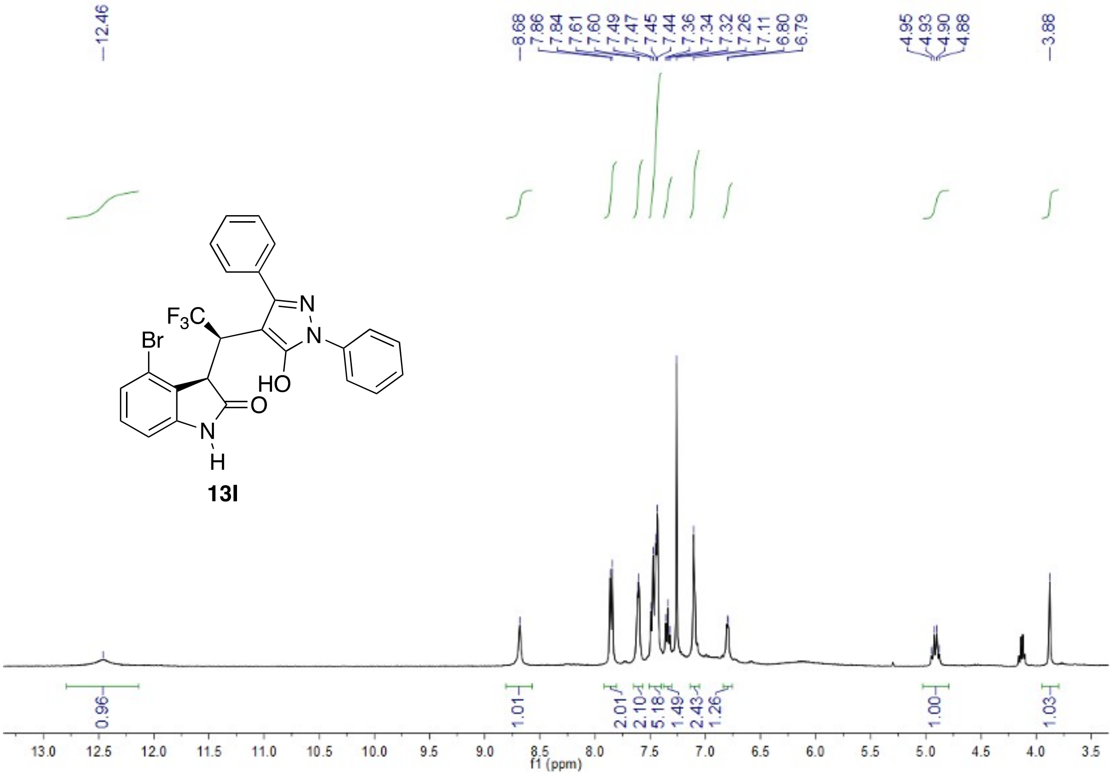

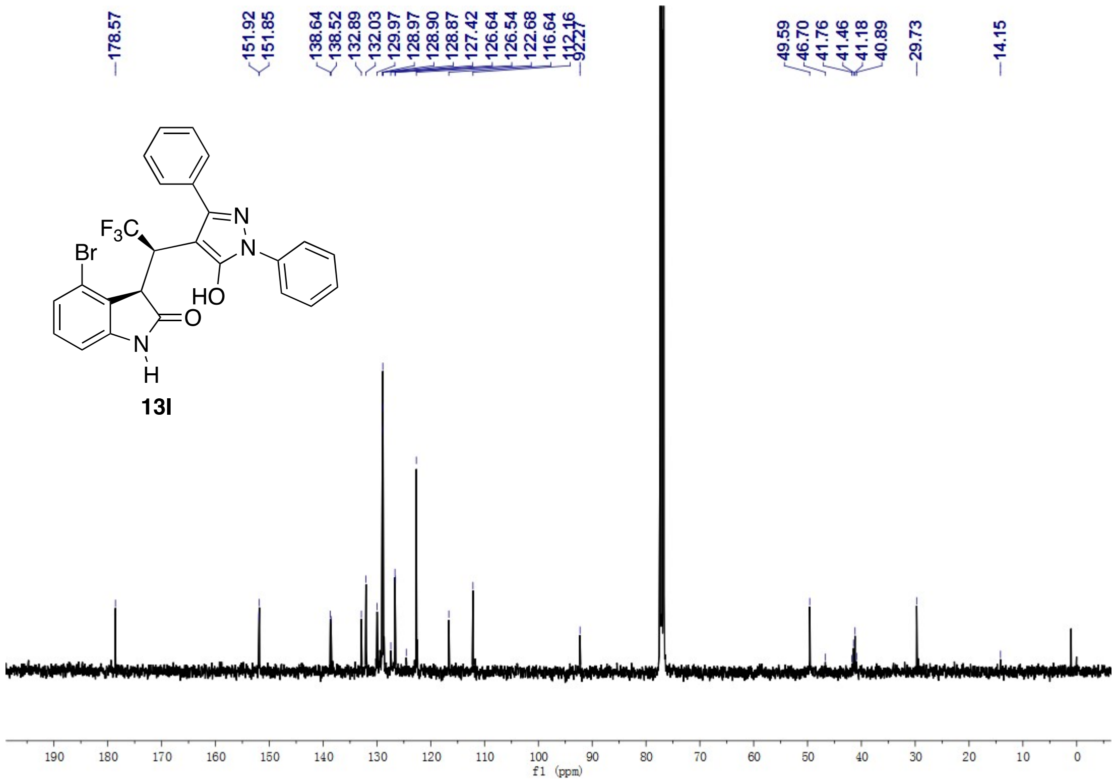
178.57
151.92
151.85
138.64
138.52
132.89
132.03
129.97
128.97
128.90
128.87
127.42
126.64
126.54
122.68
116.64
112.16
92.27
49.59
46.70
41.76
41.46
41.18
40.89
29.73
14.15
Br
$F_3C$
N
N
HO
O
N
H
13l
f1 (ppm)

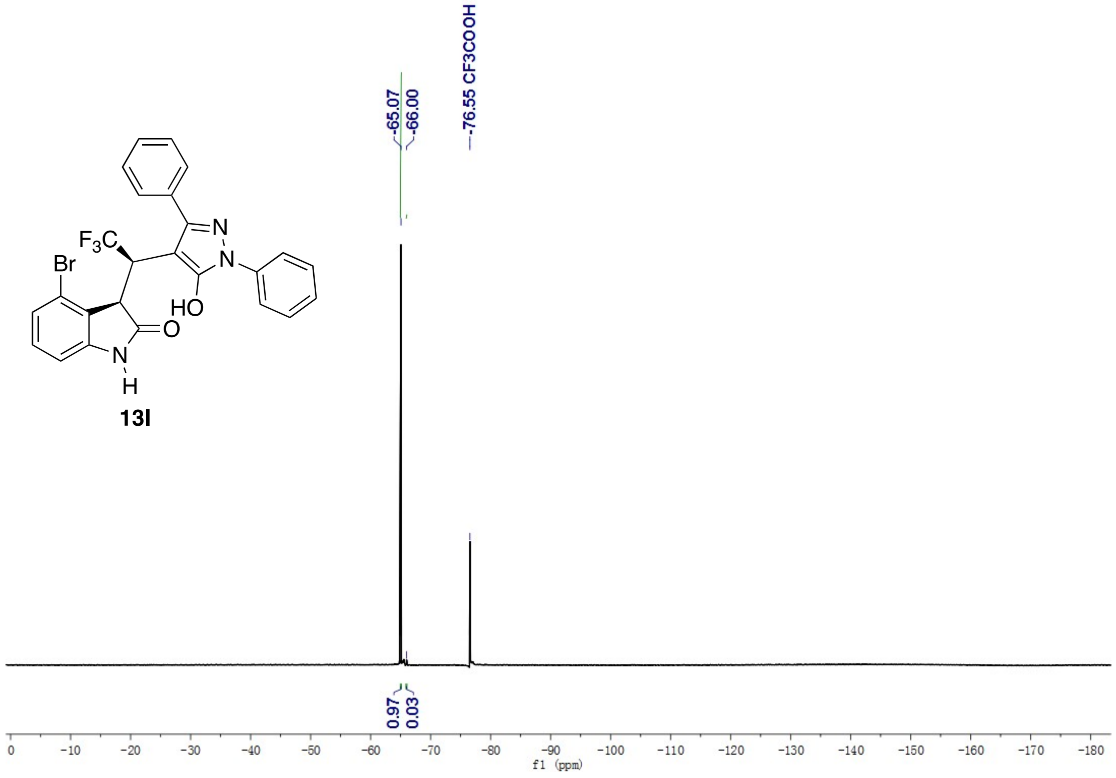
-65.07
-66.00
-76.55 CF3COOH
Br
$F_3C$
N
N
HO
O
N
H
13l
0.97
0.03
f1 (ppm)

**(*R*)-6-bromo-3-((*R*)-2,2,2-trifluoro-1-(5-hydroxy-1,3-diphenyl-1*H*-pyrazol-4-yl)ethyl)indolin-2-one (13m)**

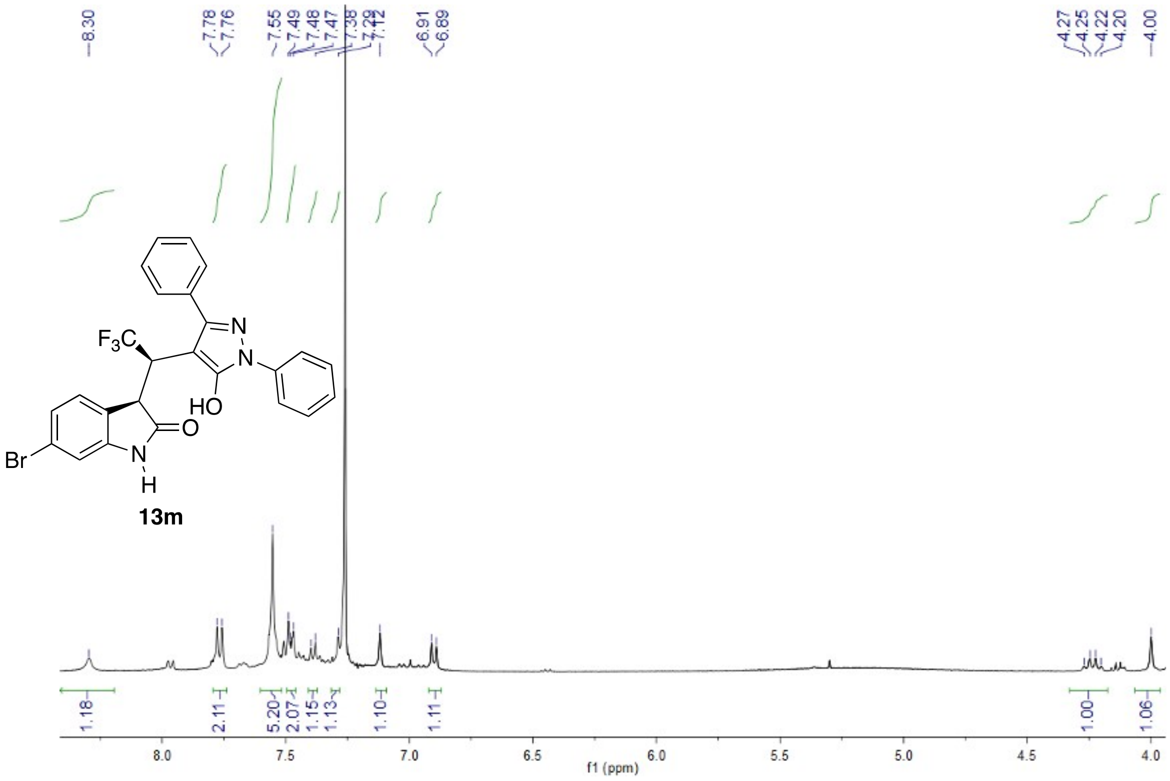


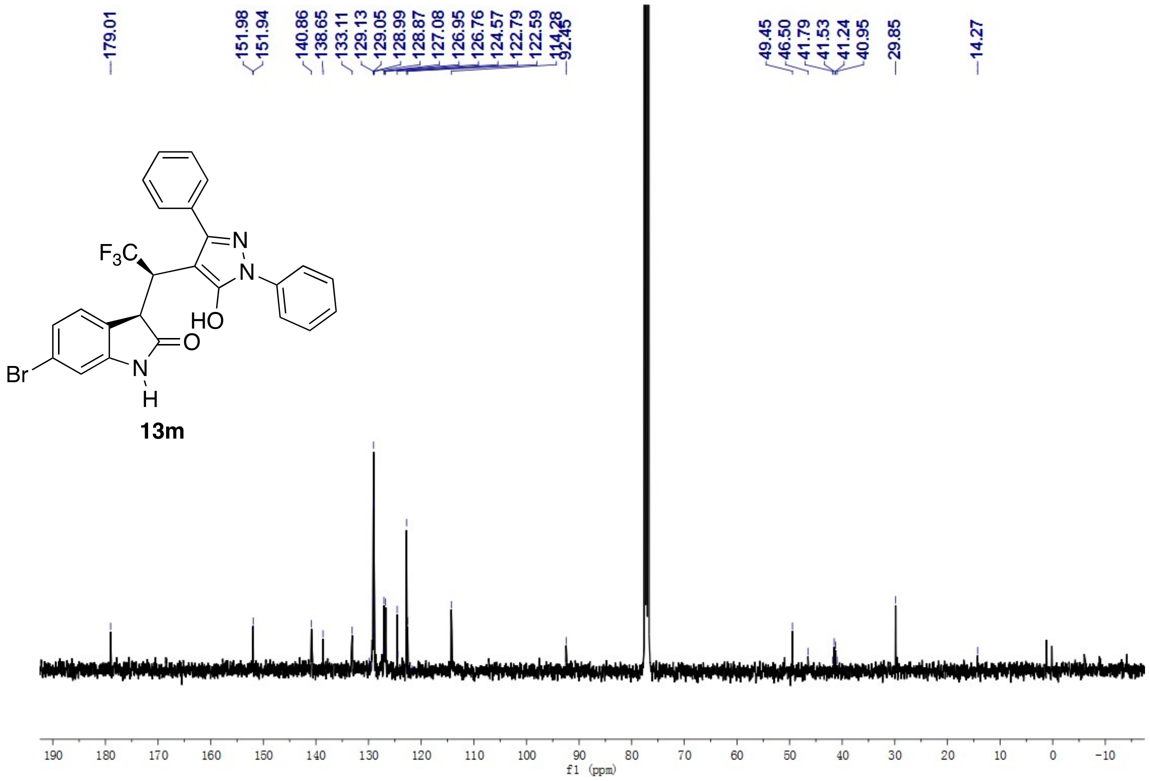

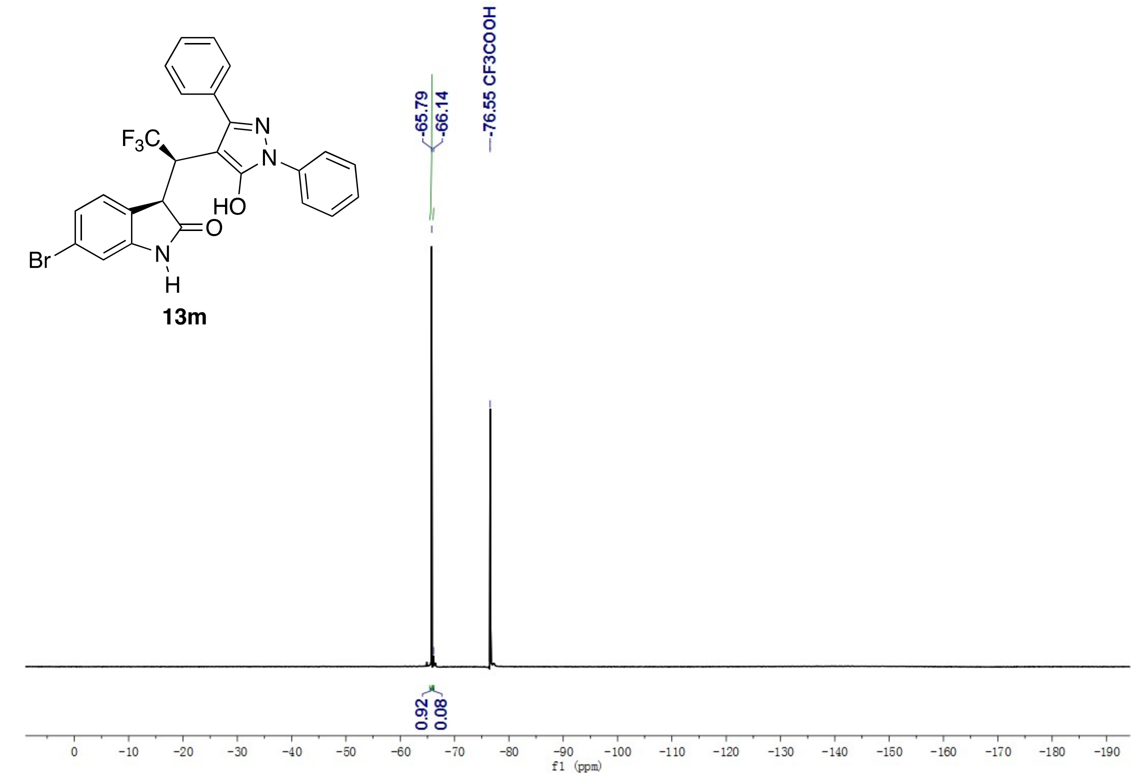


**(*R*)-4-methyl-3-((*R*)-2,2,2-trifluoro-1-(5-hydroxy-1,3-diphenyl-1*H*-pyrazol-4-yl)ethyl)indolin-2-one (13n)**

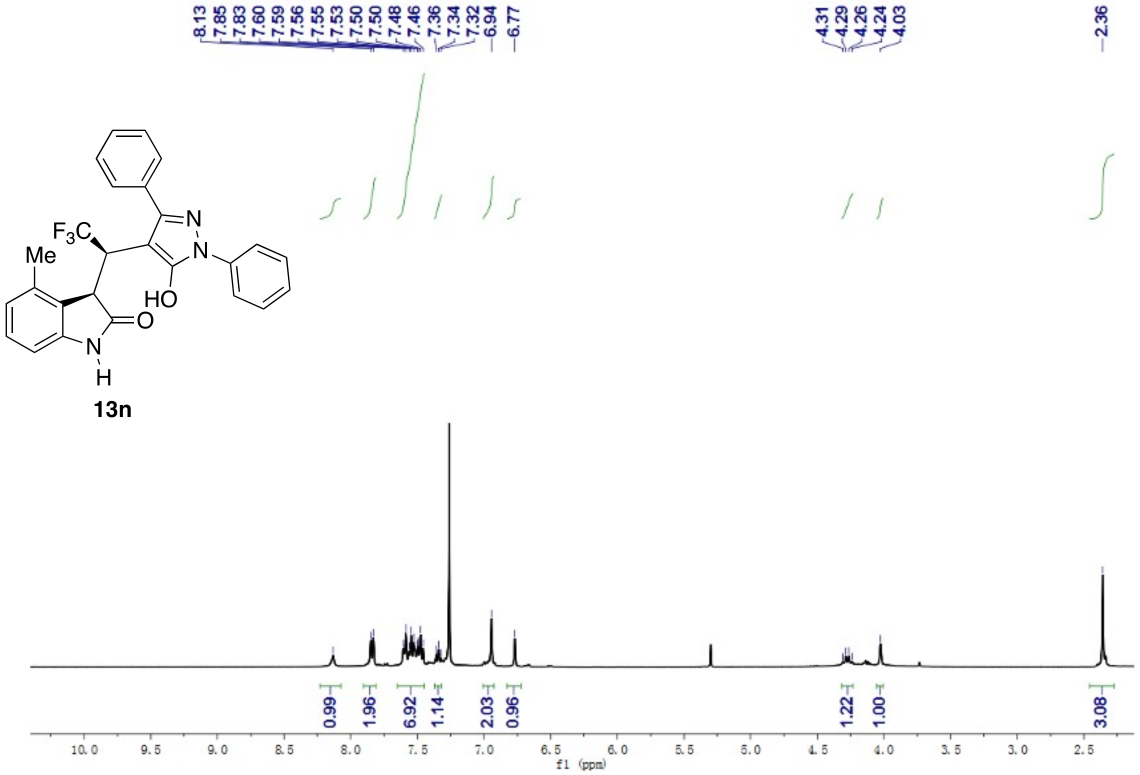

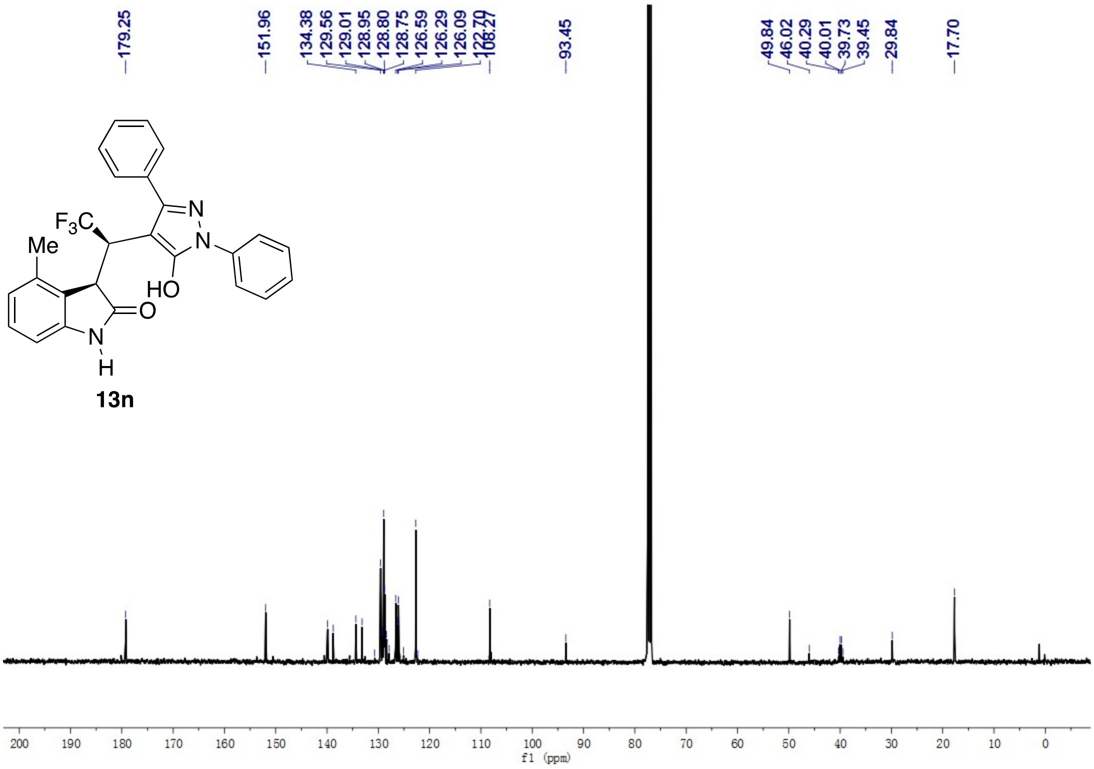
179.25
151.96
134.38
129.56
129.01
128.95
128.80
128.75
126.59
126.29
126.09
122.70
108.27
93.45
49.84
46.02
40.29
40.01
39.73
39.45
29.84
17.70
F3C
Me
N
N
HO
O
N
H
13n
f1 (ppm)

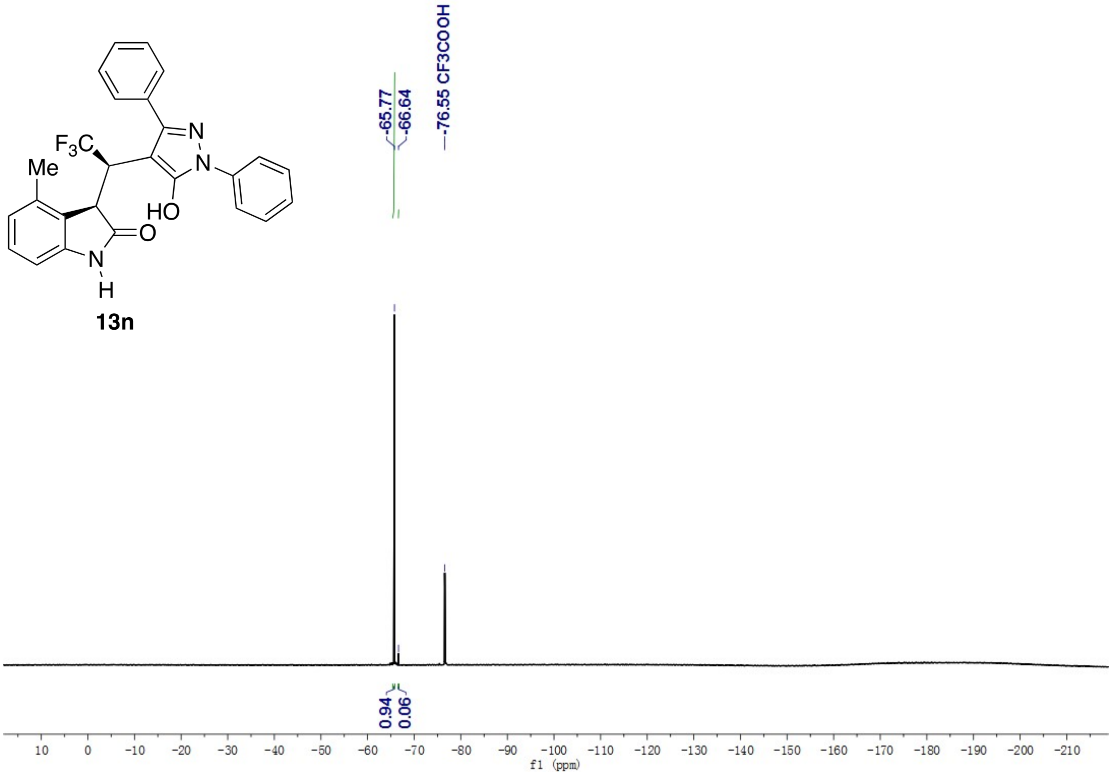
-65.77
-66.64
-76.55 CF3COOH
F3C
Me
N
N
HO
O
N
H
13n
0.94
0.06
f1 (ppm)

**Methyl (*R*)-2-(5-hydroxy-1,3-diphenyl-1*H*-pyrazol-4-yl)-2-((*R*)-2-oxoindolin-3-yl)acetate (13o)**

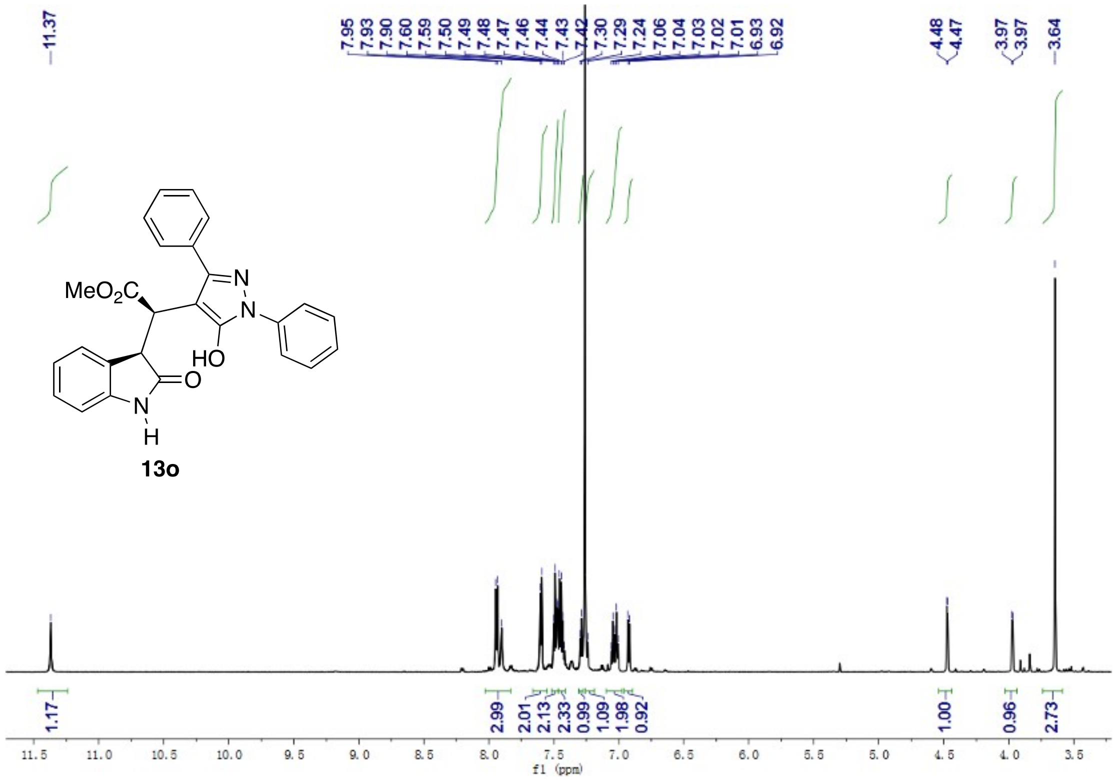


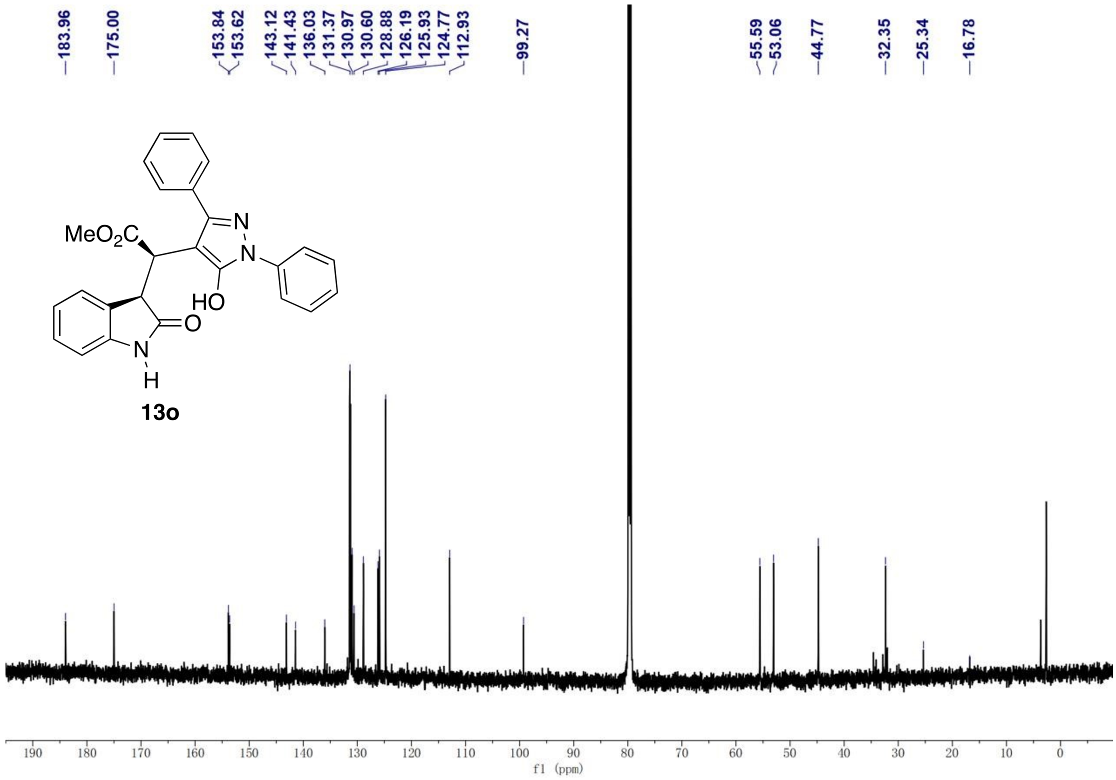

**Benzyl (*R*)-2-(5-hydroxy-1,3-diphenyl-1*H*-pyrazol-4-yl)-2-((*R*)-2-oxoindolin-3-yl)acetate (13p)**

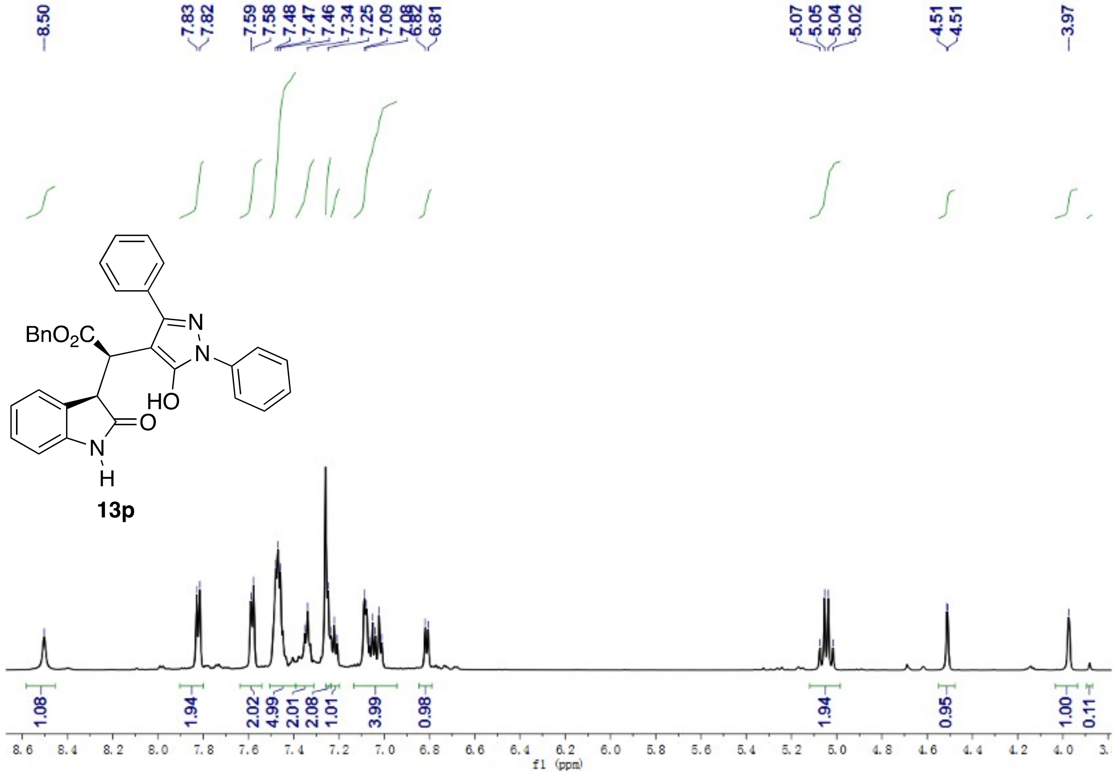


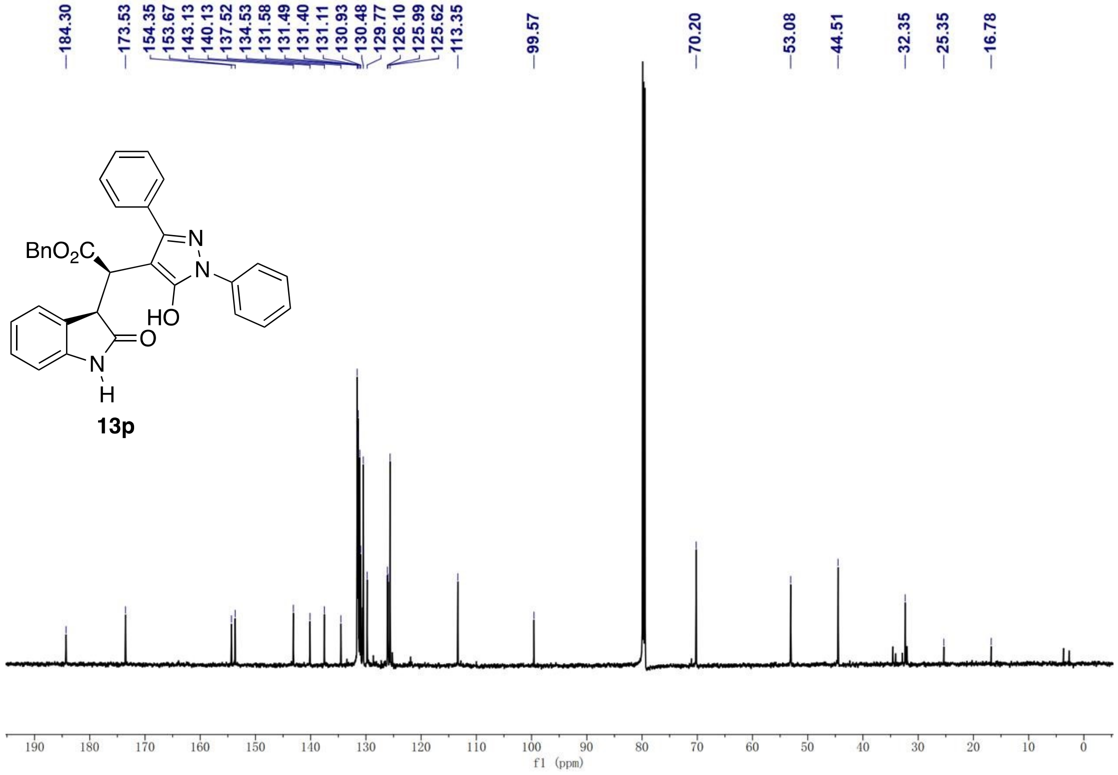

# 2 Copies of HRMS Spectra

**(*R*)-(3,3,3',3',5,5'-hexamethyl-2,2',3,3'-tetrahydro-1,1'-spirobi[indene]-7,7'-diyl)dimethanol [(*R*)-5]**

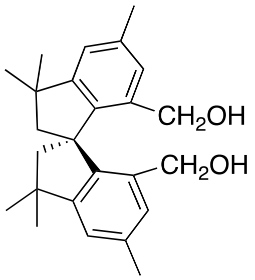


(*R*)-**5**

**Spectra**

| Fragmentor Voltage | Collision Energy | Ionization Mode |
|---|---|---|
| 180 | 0 | ESI |

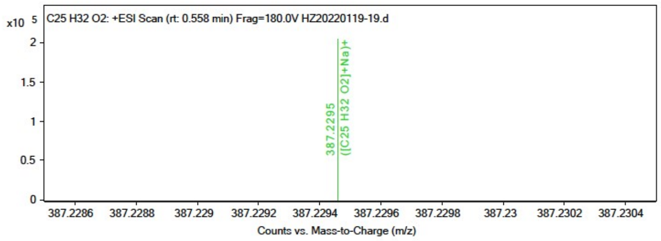


**Formula Calculator Element Limits**

| Element | Min | Max |
|---|---|---|
| C | 0 | 100 |
| H | 0 | 200 |
| O | 0 | 10 |

**Formula Calculator Results**

| Formula | Best | Measured Mass | Tgt Mass | Diff (ppm) | Score |
|---|---|---|---|---|---|
| C25 H32 Na O2 | True | 387.2295 | 387.2295 | 0.03 | 100 |

--- End Of Report ---

**(*R*)-7,7'-bis(bromomethyl)-3,3,3',3',5-pentamethyl-2,2',3,3'-tetrahydro-1,1'-spirobi[indene] [(*R*)-6]**

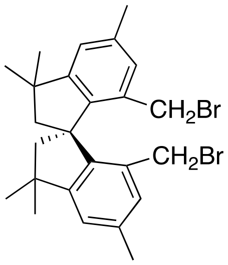


(*R*)-**6**

**Elemental Composition Report** **Page 1**

Tolerance = 5.0 mDa / DBE: min = -1.5, max = 50.0
Element prediction: Off

Monoisotopic Mass, Odd and Even Electron Ions
18 formula(e) evaluated with 1 results within limits (up to 70 closest results for each mass)
Elements Used:
C: 0-100 H: 0-200 Br: 0-2

HZ0120-20 1126 (6.083)
TOF MS EI+

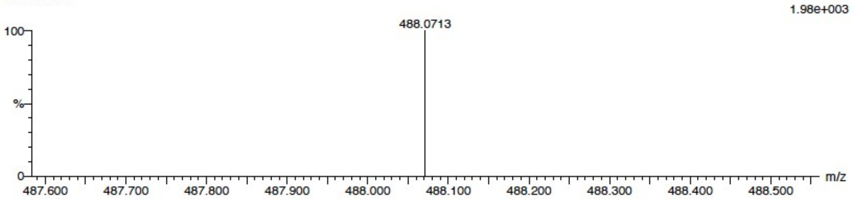


| | | | | | | |
|---|---|---|---|---|---|---|
| Minimum: | | | | -1.5 | | |
| Maximum: | | 5.0 | 10.0 | 50.0 | | |
| Mass | Calc. Mass | mDa | PPM | DBE | i-FIT | Formula |
| 488.0713 | 488.0714 | -0.1 | -0.2 | 10.0 | 5547013.5 | C25 H30 Br2 |

**(1R,2R)-2-(2,4,4,7,7,9-hexamethyl-4,5,6,7-tetrahydro-11H-diindeno[7,1-cd:1',7'-ef]azocin-12(13H)-yl)cyclohexan-1-amine (7)**

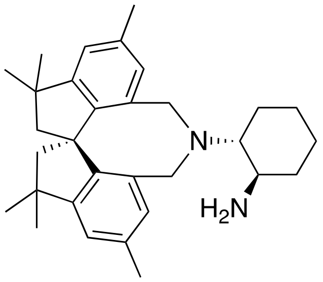

7

**Spectra**

| Fragmentor Voltage | Collision Energy | Ionization Mode |
|---|---|---|
| 180 | 0 | ESI |

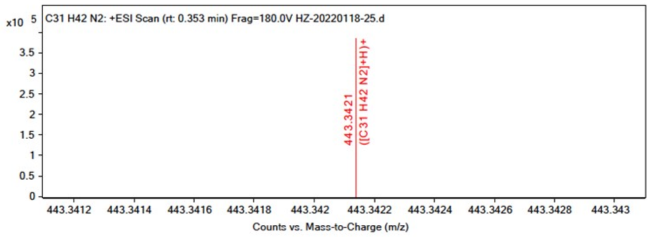


**Formula Calculator Element Limits**

| Element | Min | Max |
|---|---|---|
| C | 0 | 50 |
| H | 0 | 100 |
| O | 0 | 50 |
| N | 0 | 10 |

**Formula Calculator Results**

| Formula | Best | Measured Mass | Tgt Mass | Diff (ppm) | Score |
|---|---|---|---|---|---|
| C31 H43 N2 | True | 443.3421 | 443.3421 | 0.13 | 99.98 |

--- End Of Report ---

**3-((3,5-bis(trifluoromethyl)phenyl)amino)-4-(((1R,2R)-2-(2,4,4,7,7,9-hexamethyl-4,5,6,7-tetrahydro-11H-diindeno[7,1-cd:1',7'-ef]azocin-12(13H)-yl)cyclohexyl)amino)cyclobut-3-ene-1,2-dione (9a)**

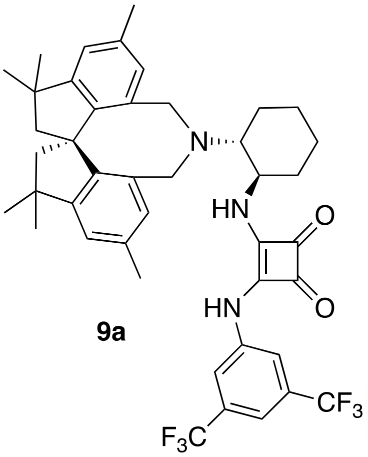

9a

**Spectra**

| Fragmentor Voltage | Collision Energy | Ionization Mode |
|---|---|---|
| 180 | 0 | ESI |

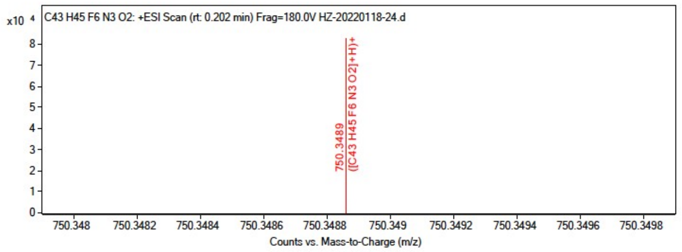


**Formula Calculator Element Limits**

| Element | Min | Max |
|---|---|---|
| C | 0 | 50 |
| H | 0 | 100 |
| O | 0 | 50 |
| N | 0 | 10 |
| F | 6 | 6 |

**Formula Calculator Results**

| Formula | Best | Measured Mass | Tgt Mass | Diff (ppm) | Score |
|---|---|---|---|---|---|
| C43 H46 F6 N3 O2 | True | 750.3489 | 750.3489 | 0.08 | 99.99 |

**2-oxo-3-(2,2,2-trifluoro-1-(5-hydroxy-1,3-diphenyl-1H-pyrazol-4-yl)ethyl)indoline-1-carboxylate (12a)**

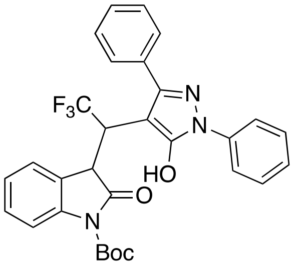


**12a**

| Fragmentor Voltage | Collision Energy | Ionization Mode |
| --- | --- | --- |
| 180 | 0 | ESI |

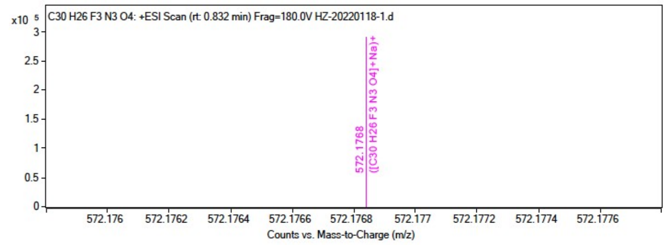


**Formula Calculator Element Limits**

| Element | Min | Max |
| --- | --- | --- |
| C | 0 | 50 |
| H | 0 | 100 |
| O | 0 | 50 |
| N | 0 | 10 |
| F | 3 | 3 |

**Formula Calculator Results**

| Formula | Best | Measured Mass | Tgt Mass | Diff (ppm) | Score |
| --- | --- | --- | --- | --- | --- |
| C30 H26 F3 N3 Na O4 | True | 572.1768 | 572.1768 | 0.02 | 100 |

--- End Of Report ---

**(*R*)-3-((*R*)-2,2,2-trifluoro-1-(5-hydroxy-1,3-diphenyl-1*H*-pyrazol-4-yl)ethyl)indolin-2-one (13a).**

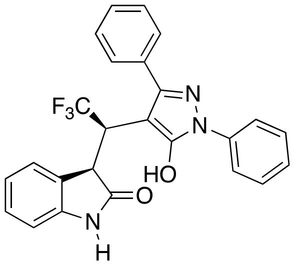


**13a**

**Spectra**

| Fragmentor Voltage | Collision Energy | Ionization Mode |
| --- | --- | --- |
| 100 | 0 | ESI |

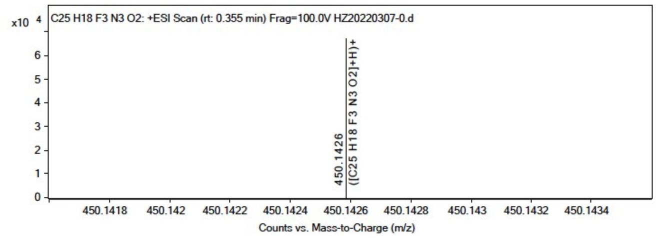


**Formula Calculator Element Limits**

| Element | Min | Max |
| --- | --- | --- |
| C | 0 | 50 |
| H | 0 | 100 |
| O | 0 | 50 |
| N | 0 | 10 |
| F | 0 | 3 |

**Formula Calculator Results**

| Formula | Best | Measured Mass | Tgt Mass | Diff (ppm) | Score |
| --- | --- | --- | --- | --- | --- |
| C25 H19 F3 N3 O2 | True | 450.1426 | 450.1424 | -0.81 | 99.35 |

--- End Of Report ---

**(*R*)-3-((*R*)-1-(3-(2-chlorophenyl)-5-hydroxy-1-phenyl-1*H*-pyrazol-4-yl)-2,2,2-trifluoroethyl)indolin-2-one (13b)**

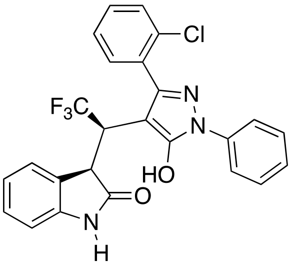


**13b**

**Spectra**

| Fragmentor Voltage | Collision Energy | Ionization Mode |
|---|---|---|
| 180 | 0 | ESI |

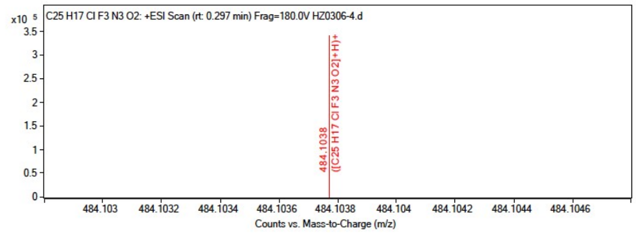


**Formula Calculator Element Limits**

| Element | Min | Max |
|---|---|---|
| C | 0 | 50 |
| H | 0 | 100 |
| O | 0 | 50 |
| N | 0 | 10 |
| F | 0 | 3 |
| Cl | 0 | 1 |

**Formula Calculator Results**

| Formula | Best | Measured Mass | Tgt Mass | Diff (ppm) | Score |
|---|---|---|---|---|---|
| C25 H18 Cl F3 N3 O2 | True | 484.1038 | 484.1034 | -0.14 | 99.98 |

--- End Of Report ---

**(*R*)-3-((*R*)-1-(3-(3-chlorophenyl)-5-hydroxy-1-phenyl-1*H*-pyrazol-4-yl)-2,2,2-trifluoroethyl)indolin-2-one (13c)**

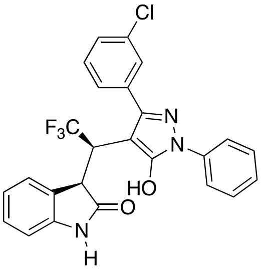


**13c**

**Spectra**

| Fragmentor Voltage | Collision Energy | Ionization Mode |
|---|---|---|
| 180 | 0 | ESI |

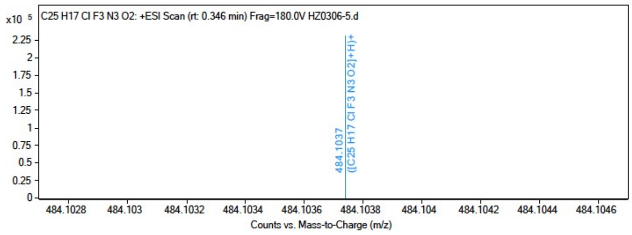


**Formula Calculator Element Limits**

| Element | Min | Max |
|---|---|---|
| C | 0 | 50 |
| H | 0 | 100 |
| O | 0 | 50 |
| N | 0 | 10 |
| F | 0 | 3 |
| Cl | 0 | 1 |

**Formula Calculator Results**

| Formula | Best | Measured Mass | Tgt Mass | Diff (ppm) | Score |
|---|---|---|---|---|---|
| C25 H18 Cl F3 N3 O2 | True | 484.1037 | 484.1034 | -0.2 | 99.96 |

--- End Of Report ---

**(*R*)-3-((*R*)-1-(3-(4-chlorophenyl)-5-hydroxy-1-phenyl-1*H*-pyrazol-4-yl)-2,2,2-trifluoroethyl)indolin-2-one (13d)**

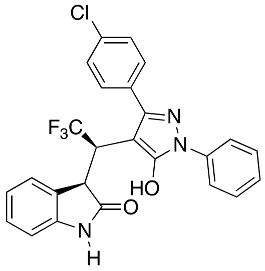


**13d**

**Spectra**

| Fragmentor Voltage | Collision Energy | Ionization Mode |
|---|---|---|
| 180 | 0 | ESI |

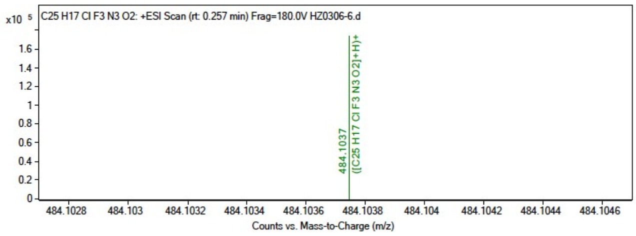


**Formula Calculator Element Limits**

| Element | Min | Max |
|---|---|---|
| C | 0 | 50 |
| H | 0 | 100 |
| O | 0 | 50 |
| N | 0 | 10 |
| F | 0 | 3 |
| Cl | 0 | 1 |

**Formula Calculator Results**

| Formula | Best | Measured Mass | Tgt Mass | Diff (ppm) | Score |
|---|---|---|---|---|---|
| C25 H18 Cl F3 N3 O2 | True | 484.1037 | 484.1034 | -0.57 | 99.66 |

--- End Of Report ---

**(*R*)-3-((*R*)-2,2,2-trifluoro-1-(5-hydroxy-1-phenyl-3-(*p*-tolyl)-1*H*-pyrazol-4-yl)ethyl)indolin-2-one (13e)**

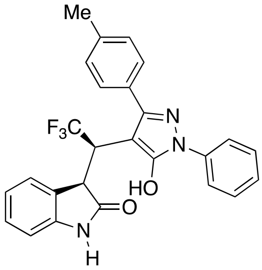


**13e**

**Spectra**

| Fragmentor Voltage | Collision Energy | Ionization Mode |
|---|---|---|
| 180 | 0 | ESI |

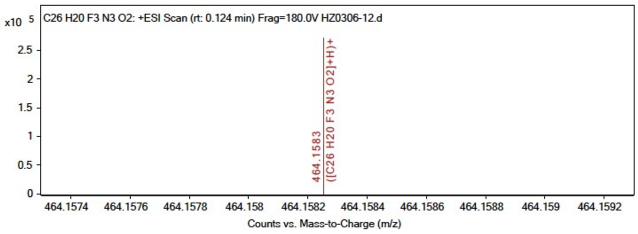


**Formula Calculator Element Limits**

| Element | Min | Max |
|---|---|---|
| C | 0 | 50 |
| H | 0 | 100 |
| O | 0 | 50 |
| N | 0 | 10 |
| F | 0 | 3 |

**Formula Calculator Results**

| Formula | Best | Measured Mass | Tgt Mass | Diff (ppm) | Score |
|---|---|---|---|---|---|
| C26 H21 F3 N3 O2 | True | 464.1583 | 464.158 | -0.11 | 99.99 |

--- End Of Report ---

**(*R*)-3-((*R*)-2,2,2-trifluoro-1-(5-hydroxy-1-phenyl-3-(*m*-tolyl)-1*H*-pyrazol-4-yl)ethyl)indolin-2-one (13f)**

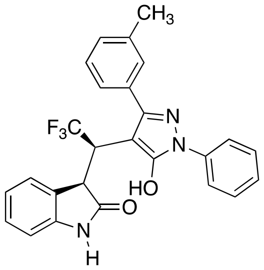


**13f**

Spectra

| Fragmentor Voltage | Collision Energy | Ionization Mode |
|---|---|---|
| 180 | 0 | ESI |

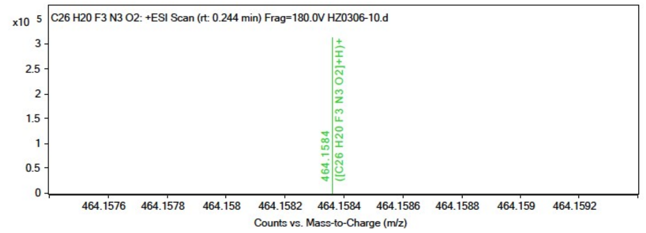


Formula Calculator Element Limits

| Element | Min | Max |
|---|---|---|
| C | 0 | 50 |
| H | 0 | 100 |
| O | 0 | 50 |
| N | 0 | 10 |
| F | 0 | 3 |

Formula Calculator Results

| Formula | Best | Measured Mass | Tgt Mass | Diff (ppm) | Score |
|---|---|---|---|---|---|
| C26 H21 F3 N3 O2 | True | 464.1584 | 464.158 | -0.51 | 99.74 |

--- End Of Report ---

**(*R*)-3-((*R*)-2,2,2-trifluoro-1-(5-hydroxy-1-(2-methoxyphenyl)-3-phenyl-1*H*-pyrazol-4-yl)ethyl)indolin-2-one (13g)**

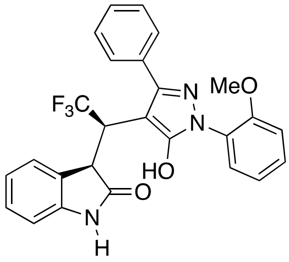


**13g**

Spectra

| Fragmentor Voltage | Collision Energy | Ionization Mode |
|---|---|---|
| 180 | 0 | ESI |

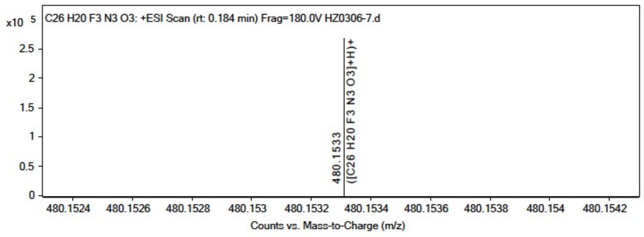


Formula Calculator Element Limits

| Element | Min | Max |
|---|---|---|
| C | 0 | 50 |
| H | 0 | 100 |
| O | 0 | 50 |
| N | 0 | 10 |
| F | 0 | 3 |

Formula Calculator Results

| Formula | Best | Measured Mass | Tgt Mass | Diff (ppm) | Score |
|---|---|---|---|---|---|
| C26 H21 F3 N3 O3 | True | 480.1533 | 480.153 | -0.46 | 99.77 |

--- End Of Report ---

**(*R*)-3-((*R*)-2,2,2-trifluoro-1-(5-hydroxy-1-(4-methoxyphenyl)-3-phenyl-1*H*-pyrazol-4-yl)ethyl)indolin-2-one (13h)**

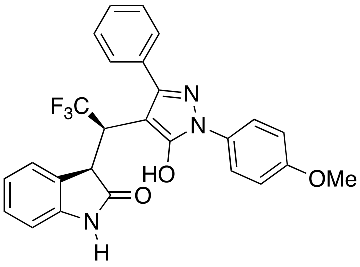


**13h**

**Spectra**

| Fragmentor Voltage | Collision Energy | Ionization Mode |
|---|---|---|
| 180 | 0 | ESI |

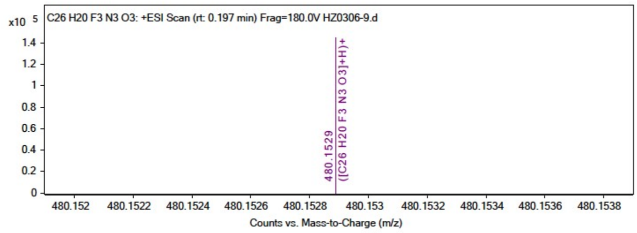


**Formula Calculator Element Limits**

| Element | Min | Max |
|---|---|---|
| C | 0 | 50 |
| H | 0 | 100 |
| O | 0 | 50 |
| N | 0 | 10 |
| F | 0 | 3 |

**Formula Calculator Results**

| Formula | Best | Measured Mass | Tgt Mass | Diff (ppm) | Score |
|---|---|---|---|---|---|
| C26 H21 F3 N3 O3 | True | 480.1529 | 480.153 | 0.19 | 99.96 |

--- End Of Report ---

**(*R*)-3-((*R*)-1-(1-(4-chlorophenyl)-5-hydroxy-3-phenyl-1*H*-pyrazol-4-yl)-2,2,2-trifluoroethyl)indolin-2-one (13i)**

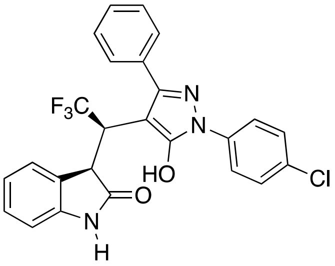


**13i**

| Fragmentor Voltage | Collision Energy | Ionization Mode |
|---|---|---|
| 180 | 0 | ESI |

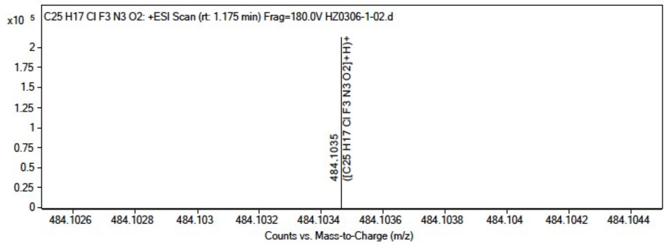


**Formula Calculator Element Limits**

| Element | Min | Max |
|---|---|---|
| C | 0 | 50 |
| H | 0 | 100 |
| O | 0 | 50 |
| N | 0 | 10 |
| F | 0 | 3 |
| Cl | 0 | 1 |

**Formula Calculator Results**

| Formula | Best | Measured Mass | Tgt Mass | Diff (ppm) | Score |
|---|---|---|---|---|---|
| C25 H18 Cl F3 N3 O2 | True | 484.1035 | 484.1034 | 0.42 | 99.81 |

--- End Of Report ---

**(*R*)-3-((*R*)-1-(1-(3-chlorophenyl)-5-hydroxy-3-phenyl-1*H*-pyrazol-4-yl)-2,2,2-trifluoroethyl)indolin-2-one (13j)**

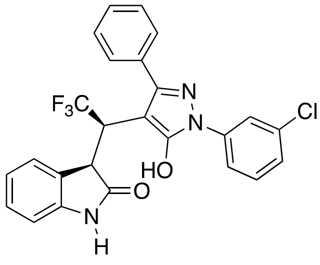


**13j**

**Spectra**

| Fragmentor Voltage | Collision Energy | Ionization Mode |
|---|---|---|
| 180 | 0 | ESI |

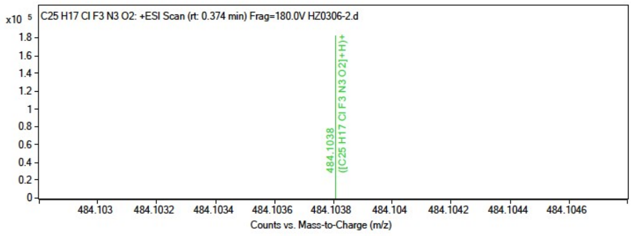


**Formula Calculator Element Limits**

| Element | Min | Max |
|---|---|---|
| C | 0 | 50 |
| H | 0 | 100 |
| O | 0 | 50 |
| N | 0 | 10 |
| F | 0 | 3 |
| Cl | 0 | 1 |

**Formula Calculator Results**

| Formula | Best | Measured Mass | Tgt Mass | Diff (ppm) | Score |
|---|---|---|---|---|---|
| C25 H18 Cl F3 N3 O2 | True | 484.1038 | 484.1034 | -0.55 | 99.68 |

--- End Of Report ---

**(*R*)-3-((*R*)-1-(1-(2-chlorophenyl)-5-hydroxy-3-phenyl-1*H*-pyrazol-4-yl)-2,2,2-trifluoroethyl)indolin-2-one (13k)**

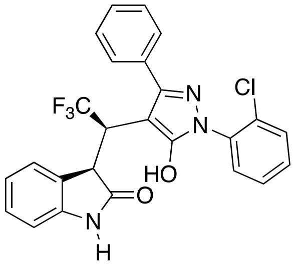


**13k**

**Spectra**

| Fragmentor Voltage | Collision Energy | Ionization Mode |
|---|---|---|
| 180 | 0 | ESI |

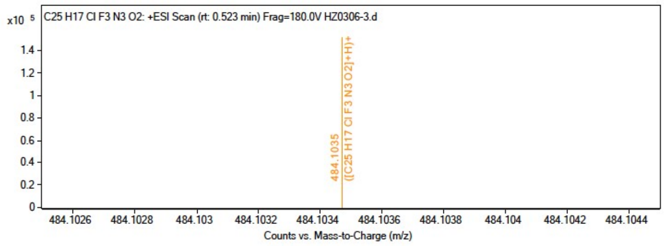


**Formula Calculator Element Limits**

| Element | Min | Max |
|---|---|---|
| C | 0 | 50 |
| H | 0 | 100 |
| O | 0 | 50 |
| N | 0 | 10 |
| F | 0 | 3 |
| Cl | 0 | 1 |

**Formula Calculator Results**

| Formula | Best | Measured Mass | Tgt Mass | Diff (ppm) | Score |
|---|---|---|---|---|---|
| C25 H18 Cl F3 N3 O2 | True | 484.1035 | 484.1034 | 0.05 | 100 |

**(*R*)-4-bromo-3-((*R*)-2,2,2-trifluoro-1-(5-hydroxy-1,3-diphenyl-1*H*-pyrazol-4-yl)ethyl)indolin-2-one (13l)**

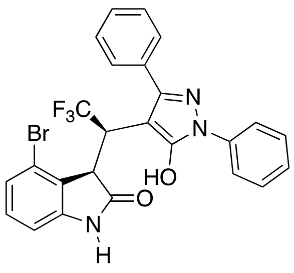

**13l**

**Spectra**

| Fragmentor Voltage | Collision Energy | Ionization Mode |
|---|---|---|
| 100 | 0 | ESI |

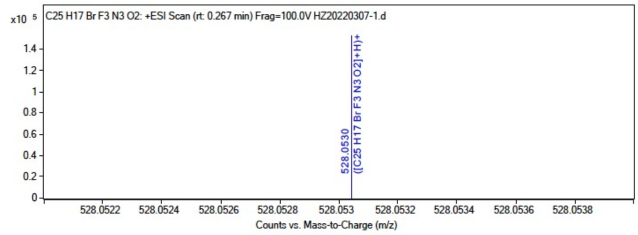


**Formula Calculator Element Limits**

| Element | Min | Max |
|---|---|---|
| C | 0 | 50 |
| H | 0 | 100 |
| O | 0 | 50 |
| N | 0 | 10 |
| F | 3 | 3 |
| Br | 1 | 1 |

**Formula Calculator Results**

| Formula | Best | Measured Mass | Tgt Mass | Diff (ppm) | Score |
|---|---|---|---|---|---|
| C25 H18 Br F3 N3 O2 | True | 528.053 | 528.0529 | -0.11 | 99.99 |

--- End Of Report ---

**(*R*)-6-bromo-3-((*R*)-2,2,2-trifluoro-1-(5-hydroxy-1,3-diphenyl-1*H*-pyrazol-4-yl)ethyl)indolin-2-one (13m)**

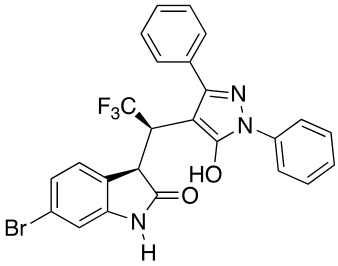

**13m**

**Spectra**

| Fragmentor Voltage | Collision Energy | Ionization Mode |
|---|---|---|
| 180 | 0 | ESI |

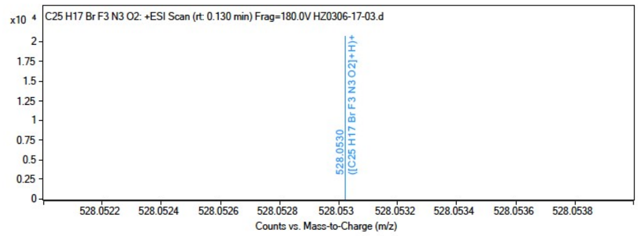


**Formula Calculator Element Limits**

| Element | Min | Max |
|---|---|---|
| C | 0 | 50 |
| H | 0 | 100 |
| O | 0 | 50 |
| N | 0 | 10 |
| F | 0 | 3 |
| Br | 0 | 1 |

**Formula Calculator Results**

| Formula | Best | Measured Mass | Tgt Mass | Diff (ppm) | Score |
|---|---|---|---|---|---|
| C25 H18 Br F3 N3 O2 | True | 528.053 | 528.0529 | 0.56 | 99.65 |

--- End Of Report ---

**(*R*)-4-methyl-3-((*R*)-2,2,2-trifluoro-1-(5-hydroxy-1,3-diphenyl-1*H*-pyrazol-4-yl)ethyl)indolin-2-one (13n)**

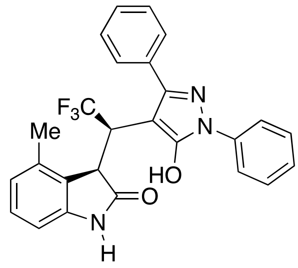

**13n**

**Spectra**

| Fragmentor Voltage | Collision Energy | Ionization Mode |
|---|---|---|
| 180 | 0 | ESI |

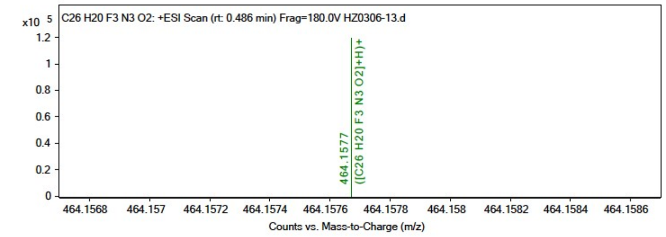


**Formula Calculator Element Limits**

| Element | Min | Max |
|---|---|---|
| C | 0 | 50 |
| H | 0 | 100 |
| O | 0 | 50 |
| N | 0 | 10 |
| F | 0 | 3 |

**Formula Calculator Results**

| Formula | Best | Measured Mass | Tgt Mass | Diff (ppm) | Score |
|---|---|---|---|---|---|
| C26 H21 F3 N3 O2 | True | 464.1577 | 464.158 | 0.87 | 99.23 |

--- End Of Report ---

**Methyl (*R*)-2-(5-hydroxy-1,3-diphenyl-1*H*-pyrazol-4-yl)-2-((*R*)-2-oxoindolin-3-yl)acetate (13o)**

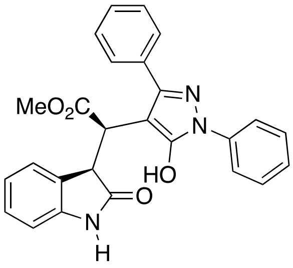

**13o**

**Spectra**

| Fragmentor Voltage | Collision Energy | Ionization Mode |
|---|---|---|
| 100 | 0 | ESI |

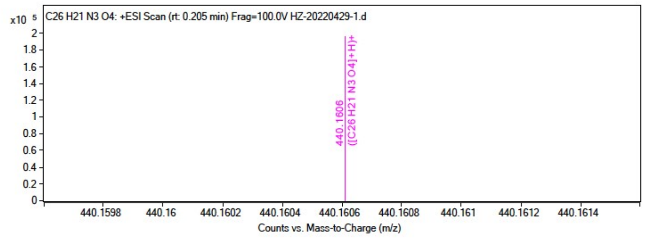


**Formula Calculator Element Limits**

| Element | Min | Max |
|---|---|---|
| C | 0 | 100 |
| H | 0 | 150 |
| O | 0 | 10 |
| N | 0 | 5 |

**Formula Calculator Results**

| Formula | Best | Measured Mass | Tgt Mass | Diff (ppm) | Score |
|---|---|---|---|---|---|
| C26 H22 N3 O4 | True | 440.1606 | 440.1605 | -0.3 | 99.91 |

--- End Of Report ---

## Benzyl (*R*)-2-(5-hydroxy-1,3-diphenyl-1*H*-pyrazol-4-yl)-2-((*R*)-2-oxoindolin-3-yl)acetate (13p)

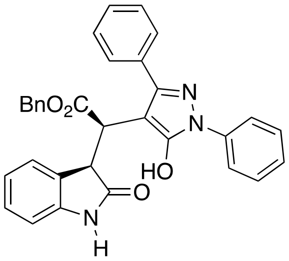


**13p**

**Spectra**

| Fragmentor Voltage | Collision Energy | Ionization Mode |
|---|---|---|
| 180 | 0 | ESI |

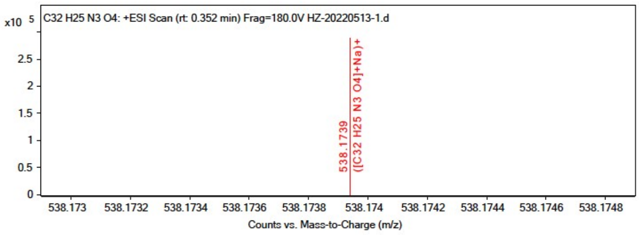


**Formula Calculator Element Limits**

| Element | Min | Max |
|---|---|---|
| C | 0 | 100 |
| H | 0 | 150 |
| O | 0 | 10 |
| N | 0 | 5 |

**Formula Calculator Results**

| Formula | Best | Measured Mass | Tgt Mass | Diff (ppm) | Score |
|---|---|---|---|---|---|
| C32 H25 N3 Na O4 | True | 538.1739 | 538.1737 | -0.24 | 99.94 |

--- End Of Report ---

# 3 Copies of HPLC spectra

**(*R*)-3-((*R*)-2,2,2-trifluoro-1-(5-hydroxy-1,3-diphenyl-1*H*-pyrazol-4-yl)ethyl)indolin-2-one (13a)**

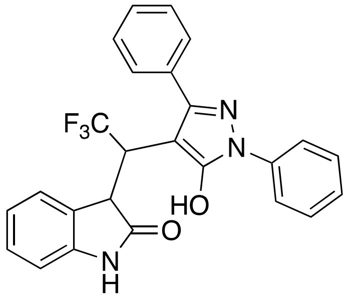


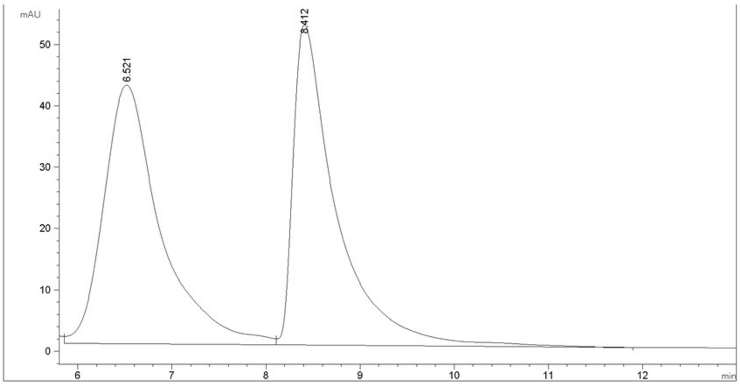


| # | [min] | | [min] | mAU *s | [mAU ] | % |
|---|---|---|---|---|---|---|
| 1 | 6.521 | VV | 0.6124 | 1769.34705 | 42.12235 | 51.5210 |
| 2 | 8.412 | VB | 0.4539 | 1664.87561 | 52.16689 | 48.4790 |

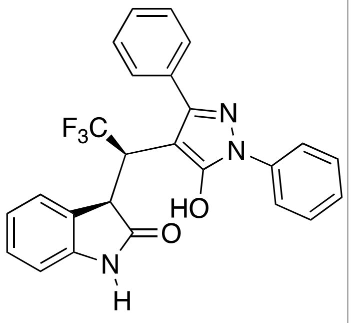


**13a**

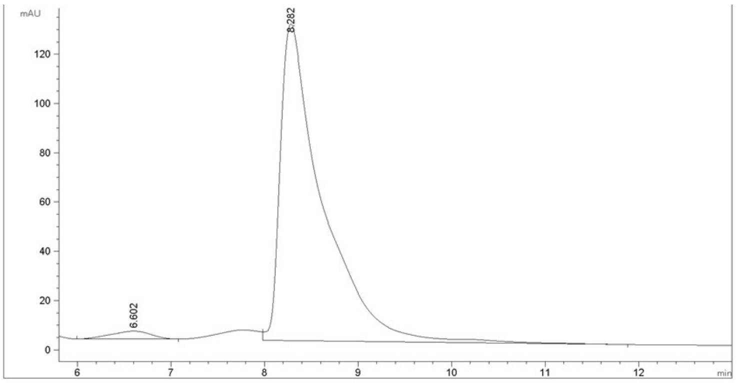


| # | [min] | | [min] | mAU *s | [mAU ] | % |
|---|---|---|---|---|---|---|
| 1 | 6.602 | BB | 0.4521 | 95.53866 | 3.20929 | 2.3195 |
| 2 | 8.282 | VB | 0.4350 | 4023.36890 | 128.54436 | 97.6805 |

(*R*)-3-((*R*)-1-(3-(2-chlorophenyl)-5-hydroxy-1-phenyl-1*H*-pyrazol-4-yl)-2,2,2-trifluoroethyl)indolin-2-one **(13b)**

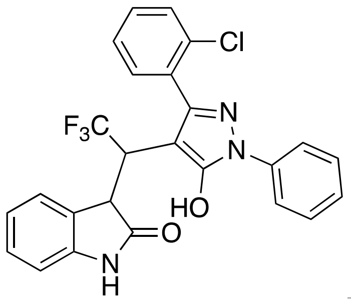


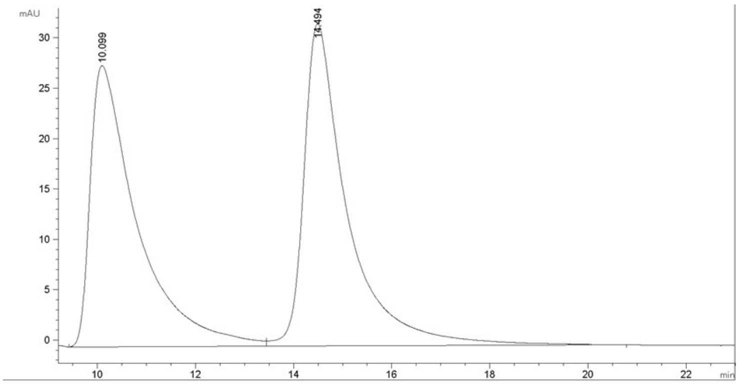


| # | [min] | | [min] | mAU *s | [mAU ] | % |
|---|---|---|---|---|---|---|
| 1 | 10.099 | BV | 0.9570 | 1842.83875 | 27.89900 | 48.3119 |
| 2 | 14.494 | VB | 0.8650 | 1971.62390 | 31.97630 | 51.6881 |

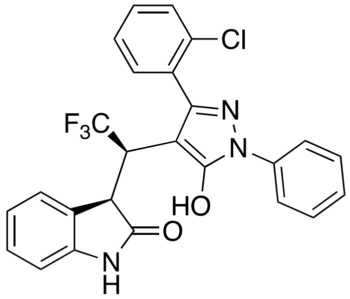


**13b**

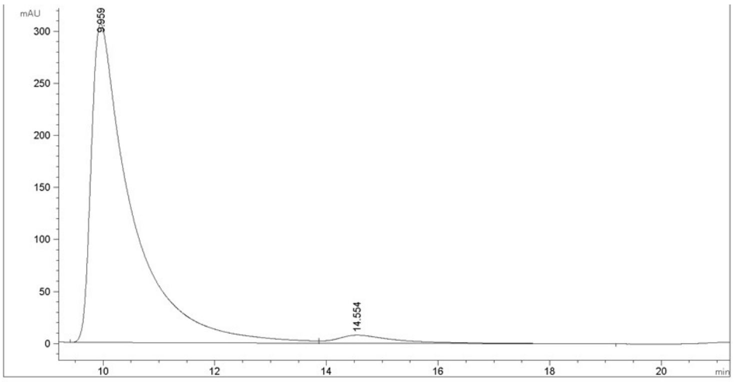


| # | [min] | | [min] | mAU *s | [mAU ] | % |
|---|---|---|---|---|---|---|
| 1 | 9.959 | BV | 0.6946 | 1.54457e4 | 306.31384 | 96.3269 |
| 2 | 14.554 | VB | 1.0500 | 588.97430 | 7.79876 | 3.6731 |

**(*R*)-3-((*R*)-1-(3-(3-chlorophenyl)-5-hydroxy-1-phenyl-1*H*-pyrazol-4-yl)-2,2,2-trifluoroethyl)indolin-2-one (13c)**

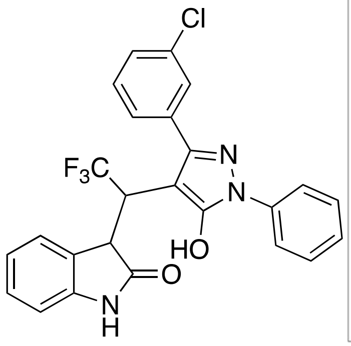


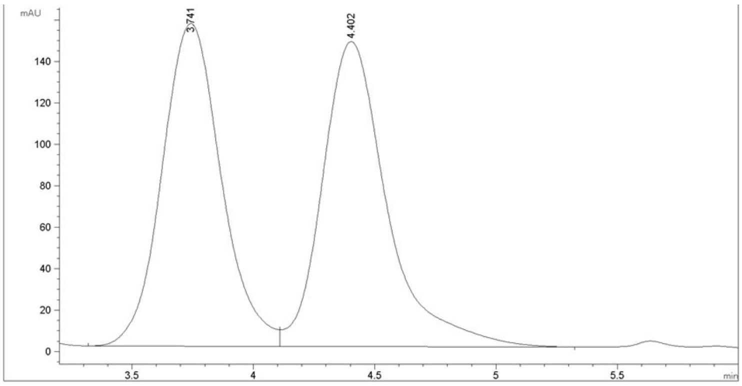


| # | [min] | | [min] | mAU *s | [mAU ] | % |
|---|---|---|---|---|---|---|
| 1 | 3.741 | BV | 0.2633 | 2639.13940 | 155.66644 | 49.2635 |
| 2 | 4.402 | VB | 0.2810 | 2718.05298 | 147.13158 | 50.7365 |

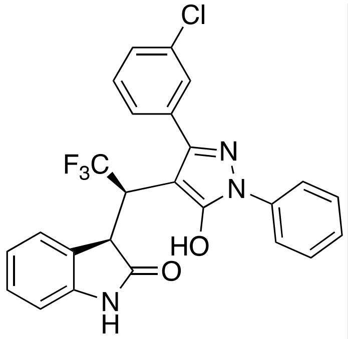


**13c**

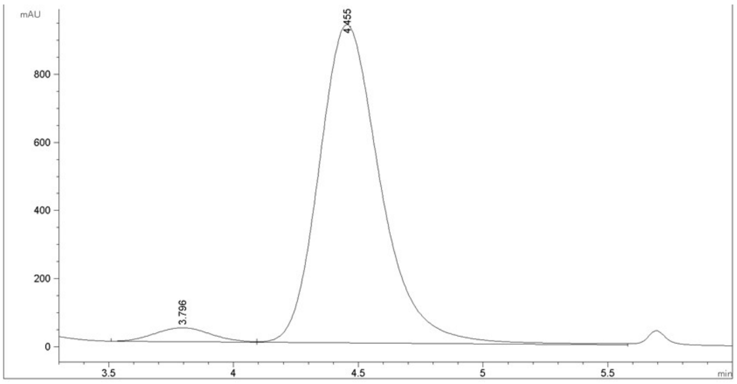


| # | [min] | | [min] | mAU *s | [mAU ] | % |
|---|---|---|---|---|---|---|
| 1 | 3.796 | BV | 0.2479 | 632.25885 | 40.77148 | 3.7987 |
| 2 | 4.455 | VV | 0.2640 | 1.60117e4 | 934.18903 | 96.2013 |

**(*R*)-3-((*R*)-1-(3-(4-chlorophenyl)-5-hydroxy-1-phenyl-1*H*-pyrazol-4-yl)-2,2,2-trifluoroethyl)indolin-2-one (13d)**

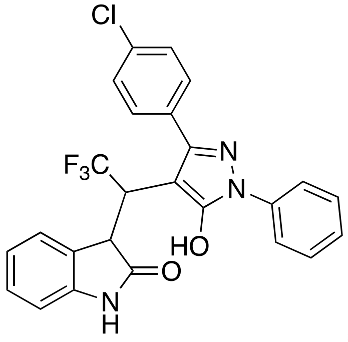


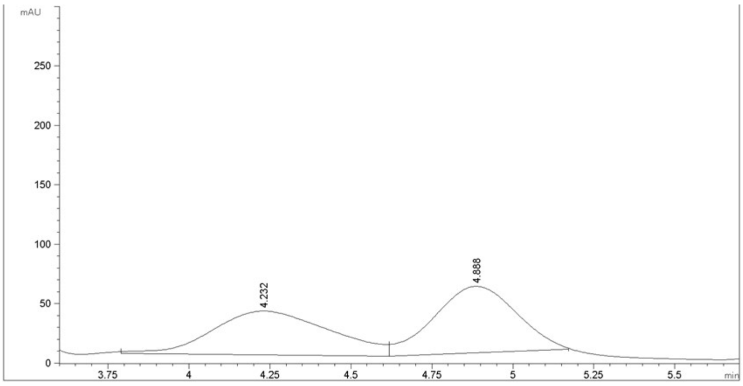


| # | [min] | | [min] | mAU *s | [mAU ] | % |
|---|---|---|---|---|---|---|
| 1 | 4.232 | MM | 0.4204 | 931.25403 | 36.91518 | 48.3801 |
| 2 | 4.888 | MM | 0.2959 | 993.61511 | 55.97254 | 51.6199 |

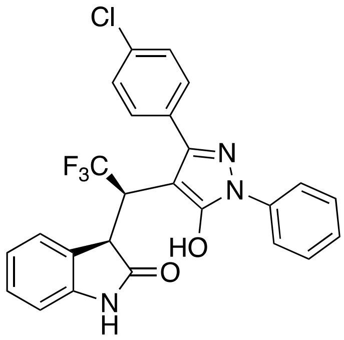


**13d**

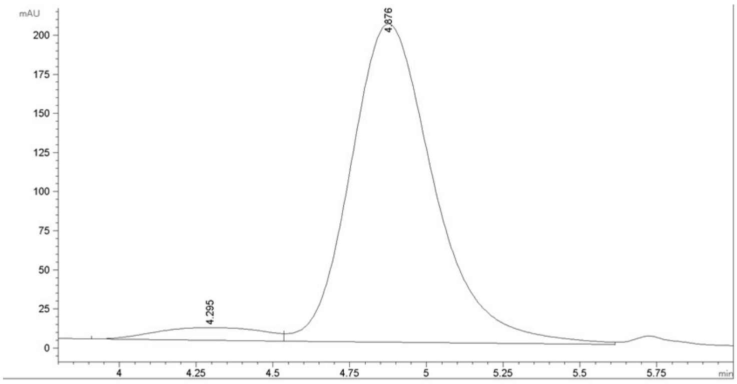


| # | [min] | | [min] | mAU *s | [mAU ] | % |
|---|---|---|---|---|---|---|
| 1 | 4.295 | BV | 0.3997 | 194.55821 | 7.93374 | 4.8360 |
| 2 | 4.876 | VV | 0.2895 | 3828.57129 | 203.42377 | 95.1640 |

**(*R*)-3-((*R*)-2,2,2-trifluoro-1-(5-hydroxy-1-phenyl-3-(*p*-tolyl)-1*H*-pyrazol-4-yl)ethyl)indolin-2-one (13e)**

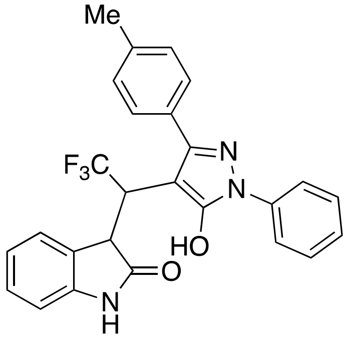


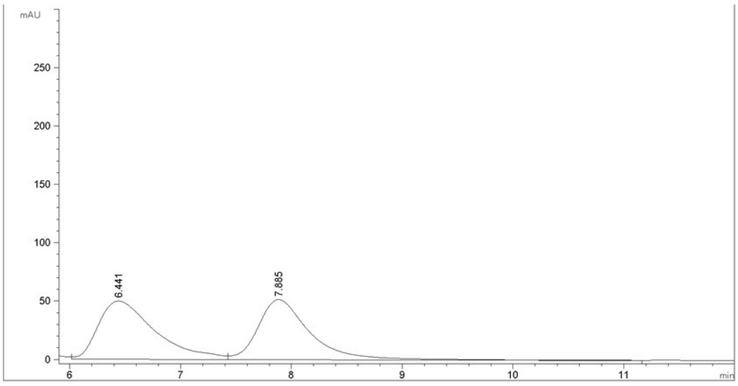


| # | [min] | | [min] | mAU *s | [mAU ] | % |
|---|---|---|---|---|---|---|
| 1 | 6.441 | VV | 0.5422 | 1787.05835 | 49.99774 | 51.5116 |
| 2 | 7.885 | VB | 0.4799 | 1682.17859 | 51.52204 | 48.4884 |

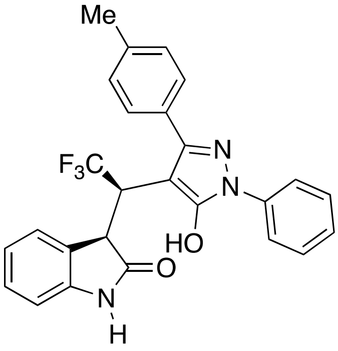


**13e**

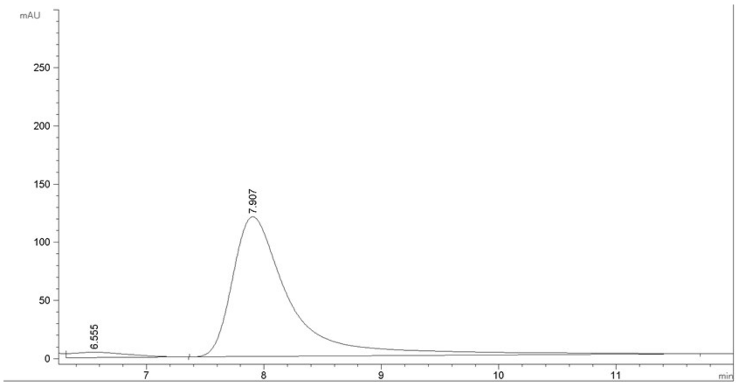


| # | [min] | | [min] | mAU *s | [mAU ] | % |
|---|---|---|---|---|---|---|
| 1 | 6.555 | VB | 0.4809 | 155.30009 | 4.63789 | 3.6008 |
| 2 | 7.907 | BB | 0.5053 | 4157.59180 | 120.23469 | 96.3992 |

**(*R*)-3-((*R*)-2,2,2-trifluoro-1-(5-hydroxy-1-phenyl-3-(*m*-tolyl)-1*H*-pyrazol-4-yl)ethyl)indolin-2-one (13f)**

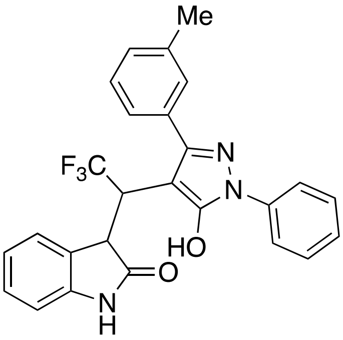


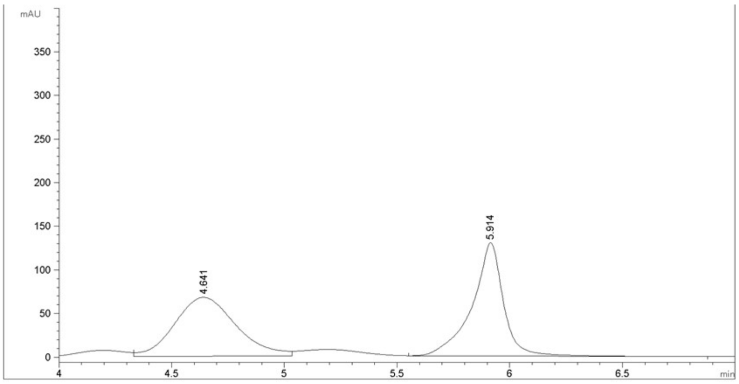


| # | [min] | | [min] | mAU *s | [mAU ] | % |
|---|---|---|---|---|---|---|
| 1 | 4.641 | VV | 0.2948 | 1285.96606 | 67.56731 | 50.6817 |
| 2 | 5.914 | BB | 0.1387 | 1251.37170 | 129.80740 | 49.3183 |

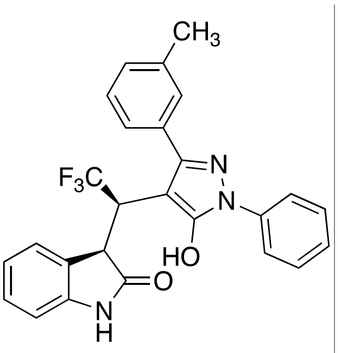


**13f**

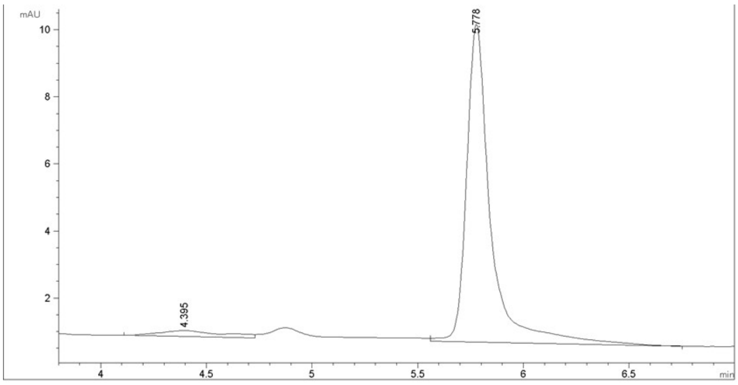


| # | [min] | | [min] | mAU *s | [mAU ] | % |
|---|---|---|---|---|---|---|
| 1 | 4.395 | BV | 0.2749 | 3.78966 | 1.77762e-1 | 4.7981 |
| 2 | 5.778 | VB | 0.1173 | 75.19232 | 9.47867 | 95.2019 |

**(*R*)-3-((*R*)-2,2,2-trifluoro-1-(5-hydroxy-1-(2-methoxyphenyl)-3-phenyl-1*H*-pyrazol-4-yl)ethyl)indolin-2-one (13g)**

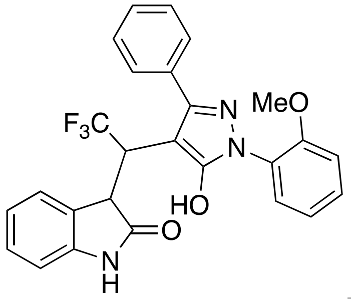


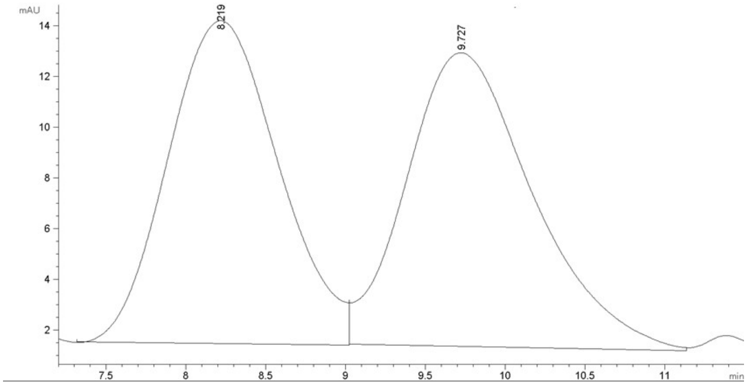


| # | [min] | | [min] | mAU *s | [mAU ] | % |
|---|---|---|---|---|---|---|
| 1 | 8.219 | MM | 0.8033 | 614.25244 | 12.74394 | 49.0322 |
| 2 | 9.727 | MM | 0.9188 | 638.50165 | 11.58276 | 50.9678 |

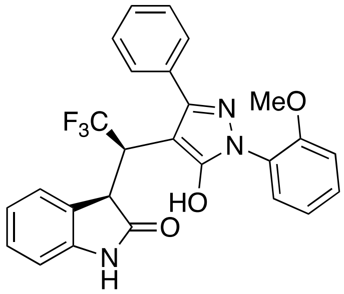


**13g**

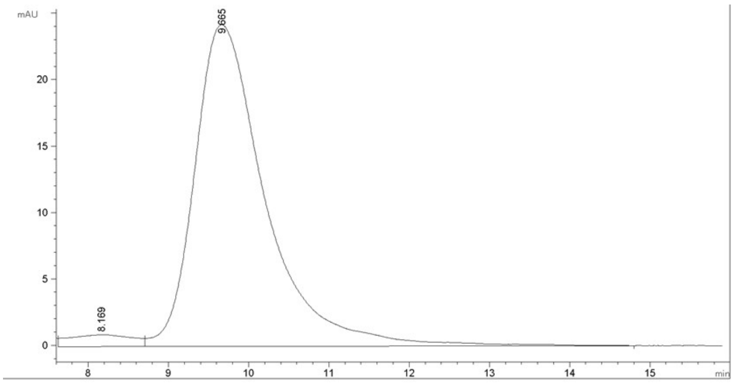


| # | [min] | | [min] | mAU *s | [mAU ] | % |
|---|---|---|---|---|---|---|
| 1 | 8.169 | VV | 0.6559 | 48.38693 | 8.76238e-1 | 3.1562 |
| 2 | 9.665 | VB | 0.9134 | 1484.71106 | 24.21462 | 96.8438 |

**(*R*)-3-((*R*)-2,2,2-trifluoro-1-(5-hydroxy-1-(4-methoxyphenyl)-3-phenyl-1*H*-pyrazol-4-yl)ethyl)indolin-2-one (13h)**

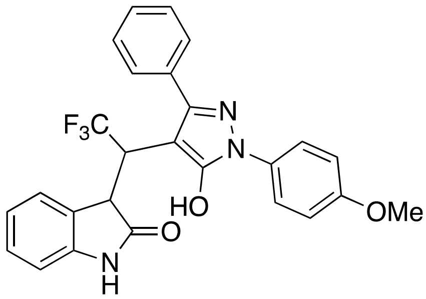


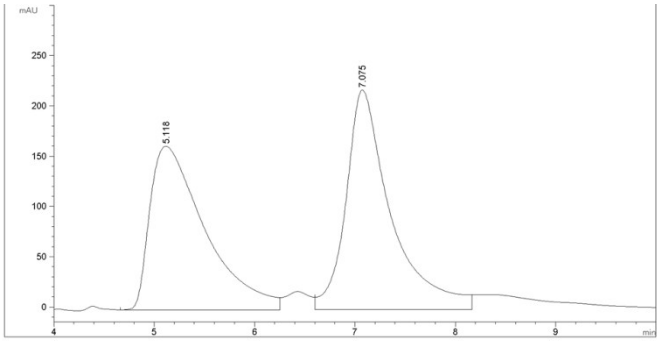


| # | [min] | | [min] | mAU *s | [mAU ] | % |
|---|---|---|---|---|---|---|
| 1 | 5.118 | BV | 0.5911 | 6213.99121 | 162.72942 | 47.7116 |
| 2 | 7.075 | VV | 0.4396 | 6810.06982 | 218.36449 | 52.2884 |

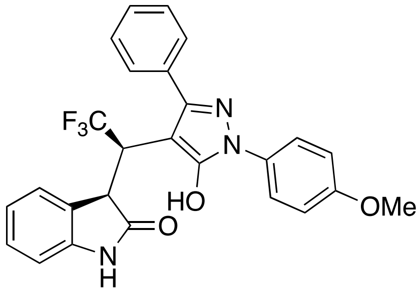


**13h**

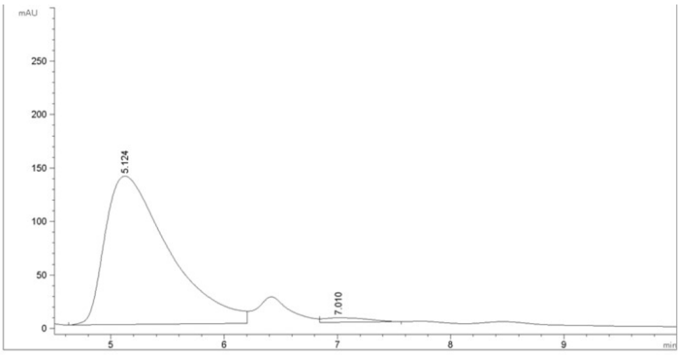


| # | [min] | | [min] | mAU *s | [mAU ] | % |
|---|---|---|---|---|---|---|
| 1 | 5.124 | BV | 0.5853 | 5435.19189 | 138.71611 | 98.0074 |
| 2 | 7.010 | VB | 0.4295 | 110.50494 | 4.19622 | 1.9926 |

**(*R*)-3-((*R*)-1-(1-(4-chlorophenyl)-5-hydroxy-3-phenyl-1*H*-pyrazol-4-yl)-2,2,2-trifluoroethyl)indolin-2-one (13i)**

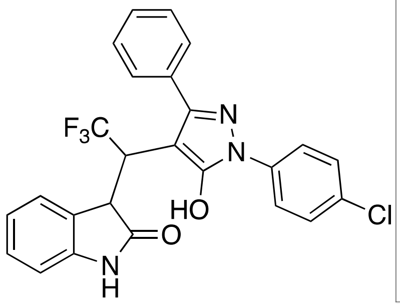


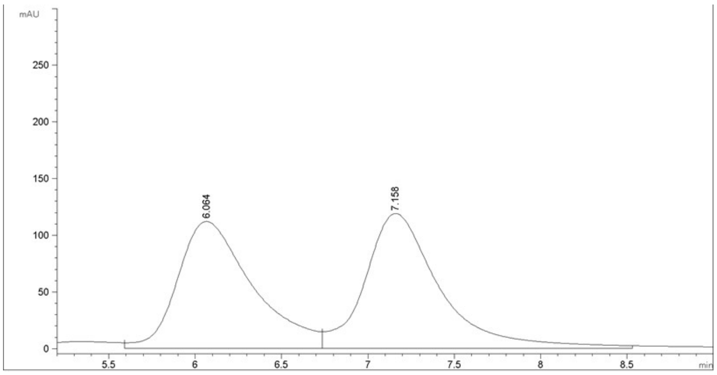


| # | [min] | | [min] | mAU *s | [mAU ] | % |
|---|---|---|---|---|---|---|
| 1 | 6.064 | VV | 0.4471 | 3365.99268 | 111.87138 | 48.4691 |
| 2 | 7.158 | MF | 0.5018 | 3578.62402 | 118.85399 | 51.5309 |

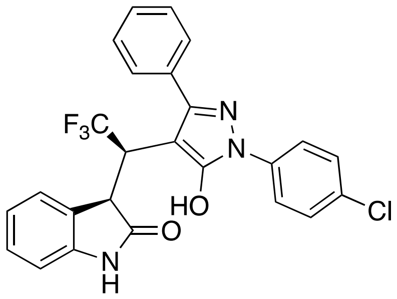


**13i**

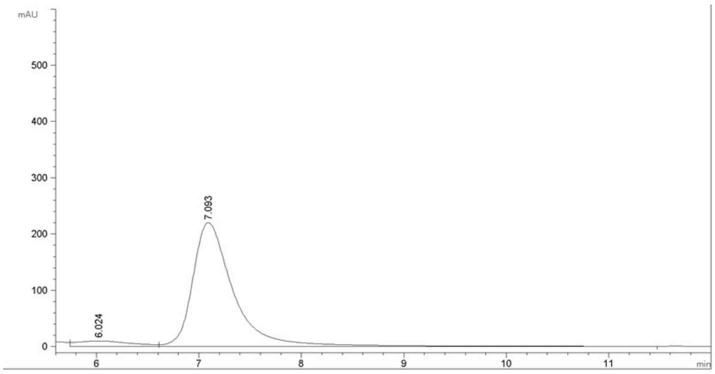


| # | [min] | | [min] | mAU *s | [mAU ] | % |
|---|---|---|---|---|---|---|
| 1 | 6.024 | VV | 0.5247 | 364.63638 | 9.99376 | 5.6476 |
| 2 | 7.093 | VB | 0.4104 | 6091.85791 | 220.03296 | 94.3524 |

(*R*)-3-((*R*)-1-(1-(3-chlorophenyl)-5-hydroxy-3-phenyl-1*H*-pyrazol-4-yl)-2,2,2-trifluoroethyl)indolin-2-one **(13j)**

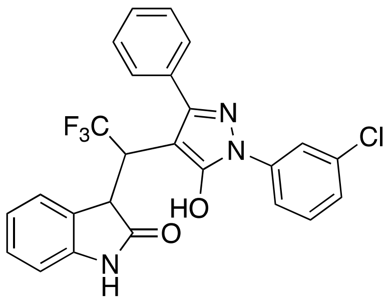


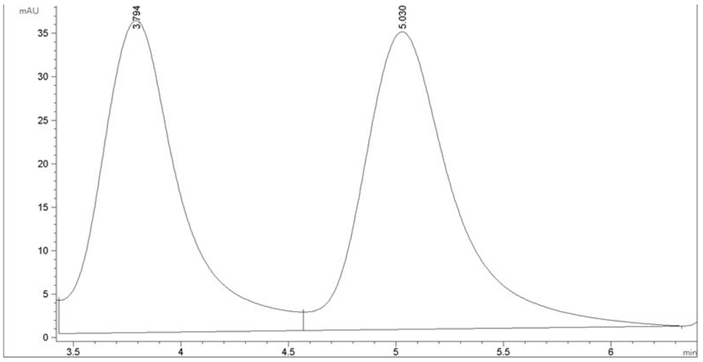


| # | [min] | | [min] | mAU *s | [mAU ] | % |
|---|---|---|---|---|---|---|
| 1 | 3.794 | VV | 0.3810 | 912.50159 | 35.89902 | 48.0653 |
| 2 | 5.030 | VB | 0.4321 | 985.96039 | 34.23189 | 51.9347 |

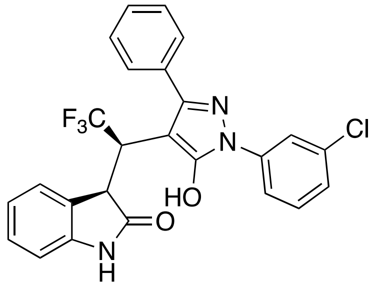


**13j**

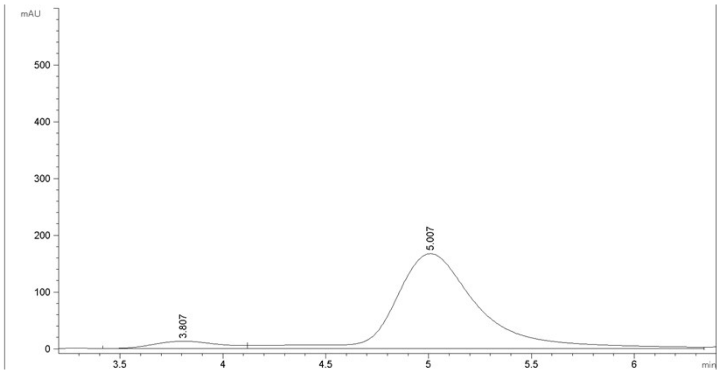


| # | [min] | | [min] | mAU *s | [mAU ] | % |
|---|---|---|---|---|---|---|
| 1 | 3.807 | BV | 0.3353 | 280.82233 | 12.76337 | 5.6598 |
| 2 | 5.007 | VV | 0.4176 | 4680.89990 | 166.84137 | 94.3402 |

**(*R*)-3-((*R*)-1-(1-(2-chlorophenyl)-5-hydroxy-3-phenyl-1*H*-pyrazol-4-yl)-2,2,2-trifluoroethyl)indolin-2-one (10k)**

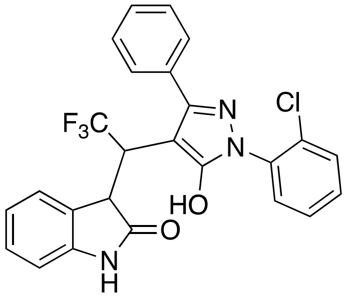


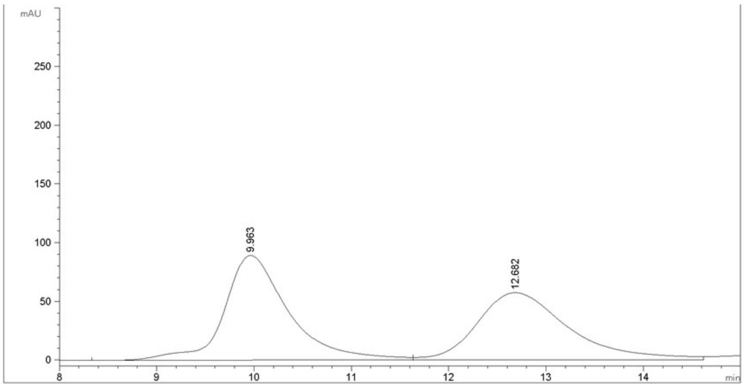


| # | [min] | | [min] | mAU *s | [mAU ] | % |
|---|---|---|---|---|---|---|
| 1 | 9.963 | BV | 0.6788 | 4125.13037 | 89.27793 | 52.4944 |
| 2 | 12.682 | VV | 0.9818 | 3733.10474 | 57.26619 | 47.5056 |

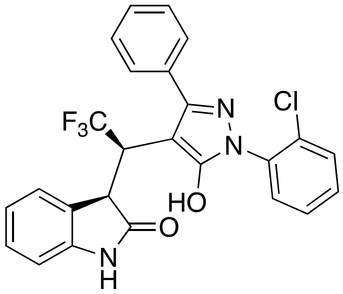


**13k**

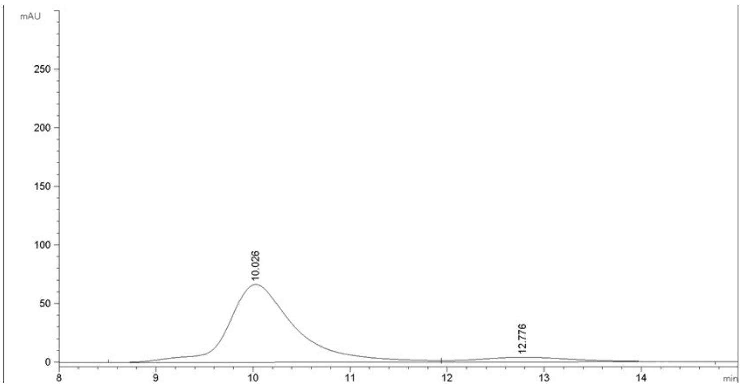


| # | [min] | | [min] | mAU *s | [mAU ] | % |
|---|---|---|---|---|---|---|
| 1 | 10.026 | BV | 0.6996 | 3171.24829 | 66.42992 | 91.7772 |
| 2 | 12.776 | VB | 1.0644 | 284.12689 | 4.07219 | 8.2228 |

**(*R*)-4-bromo-3-((*R*)-2,2,2-trifluoro-1-(5-hydroxy-1,3-diphenyl-1*H*-pyrazol-4-yl)ethyl)indolin-2-one (13l)**

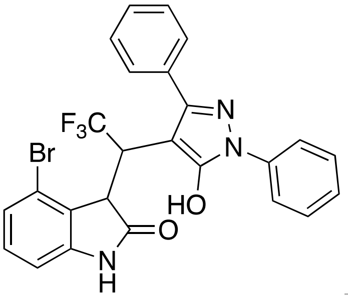


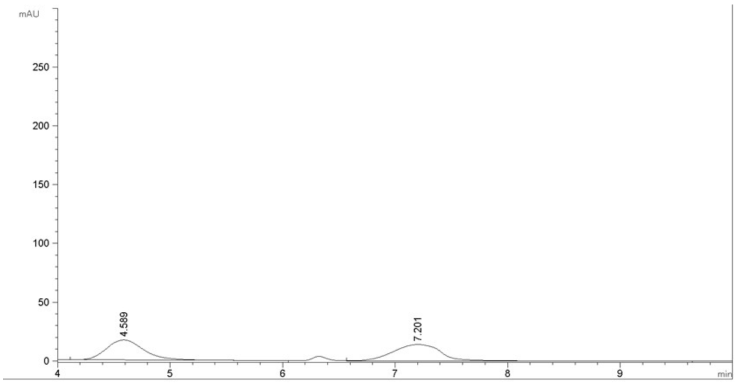


| # | [min] | | [min] | mAU *s | [mAU ] | % |
|---|---|---|---|---|---|---|
| 1 | 4.589 | BB | 0.3633 | 404.10199 | 17.08638 | 49.6677 |
| 2 | 7.201 | VB | 0.4680 | 409.50961 | 13.93612 | 50.3323 |

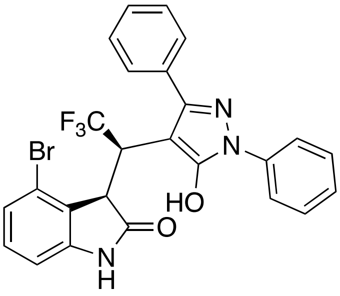


**13l**

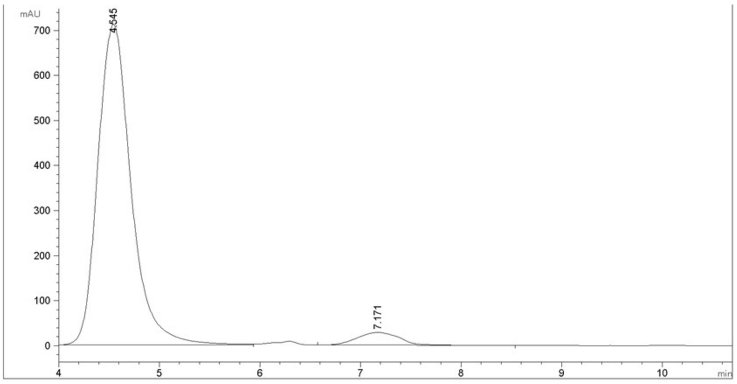


| # | [min] | | [min] | mAU *s | [mAU ] | % |
|---|---|---|---|---|---|---|
| 1 | 4.545 | BV | 0.3572 | 1.63941e4 | 712.64734 | 95.1277 |
| 2 | 7.171 | BB | 0.4820 | 839.67566 | 28.26545 | 4.8723 |

**(*R*)-6-bromo-3-((*R*)-2,2,2-trifluoro-1-(5-hydroxy-1,3-diphenyl-1*H*-pyrazol-4-yl)ethyl)indolin-2-one (13m)**

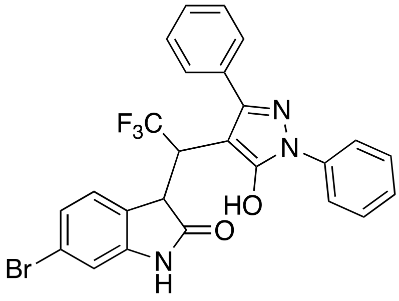


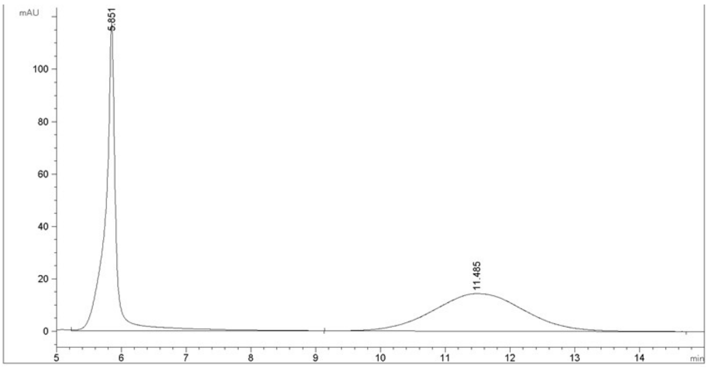


| # | [min] | | [min] | mAU *s | [mAU ] | % |
|---|---|---|---|---|---|---|
| 1 | 5.851 | VB | 0.1485 | 1322.33154 | 117.71284 | 48.6907 |
| 2 | 11.485 | BB | 1.3716 | 1393.44824 | 14.27794 | 51.3093 |

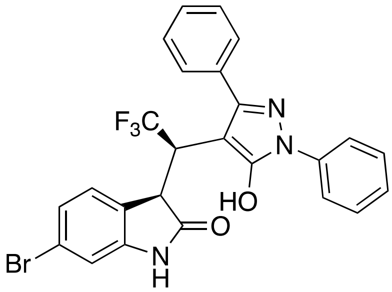


**13m**

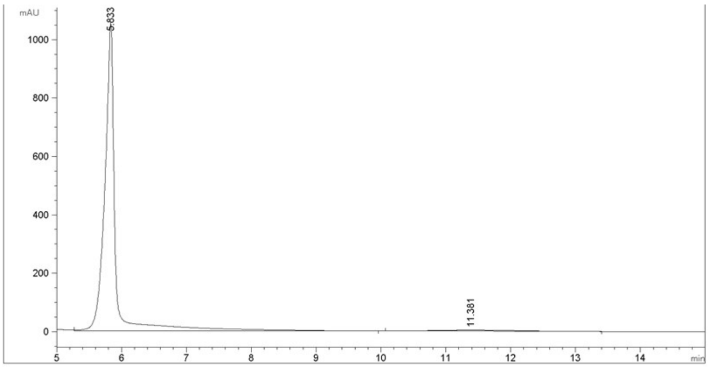


| # | [min] | | [min] | mAU *s | [mAU ] | % |
|---|---|---|---|---|---|---|
| 1 | 5.833 | VB | 0.1512 | 1.18392e4 | 1055.59583 | 97.4132 |
| 2 | 11.381 | BB | 1.0417 | 314.39594 | 3.64340 | 2.5868 |

**(*R*)-4-methyl-3-((*R*)-2,2,2-trifluoro-1-(5-hydroxy-1,3-diphenyl-1*H*-pyrazol-4-yl)ethyl)indolin-2-one (13n)**

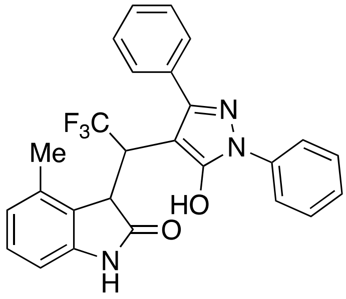

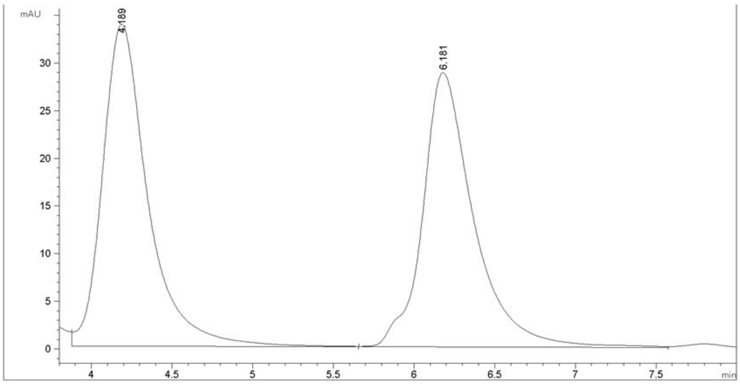


| # | [min] | | [min] | mAU *s | [mAU ] | % |
|---|---|---|---|---|---|---|
| 1 | 4.189 | VB | 0.2913 | 656.98907 | 33.73678 | 51.0095 |
| 2 | 6.181 | BV | 0.3232 | 630.98529 | 28.73274 | 48.9905 |

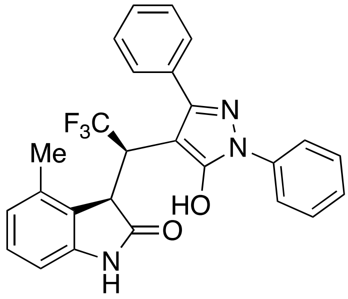

**13n**

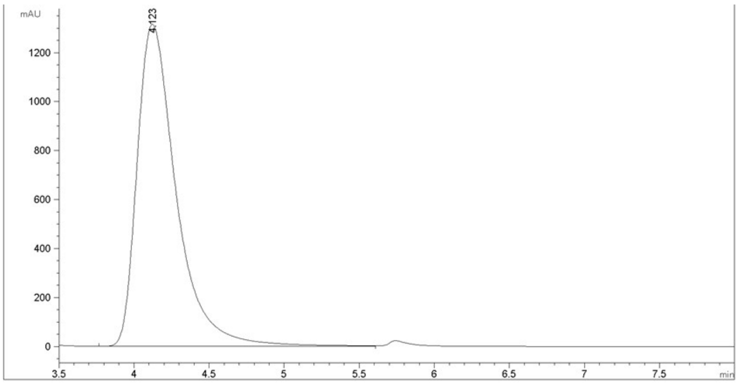


| # | [min] | | [min] | mAU *s | [mAU ] | % |
|---|---|---|---|---|---|---|
| 1 | 4.123 | BV | 0.2760 | 2.38877e4 | 1314.96423 | 100.0000 |

(*R*)-4-methyl-3-((*R*)-2,2,2-trifluoro-1-(5-hydroxy-1,3-diphenyl-1*H*-pyrazol-4-yl)ethyl)indolin-2-one **(13o)**

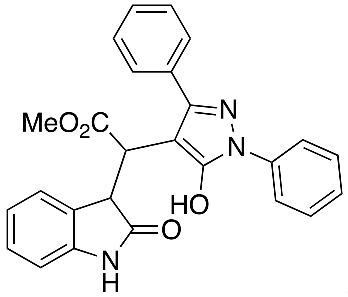


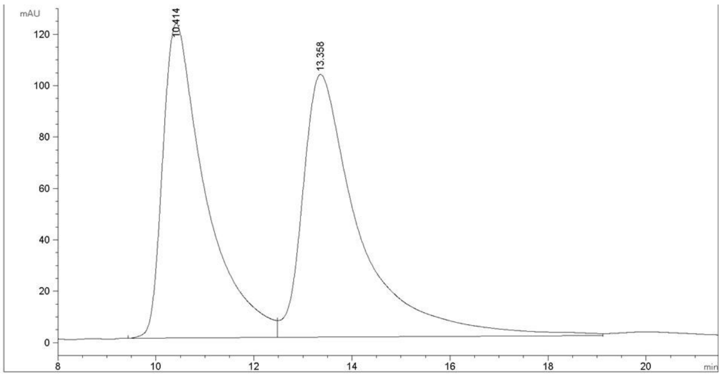


| # | [min] | | [min] | mAU *s | [mAU ] | % |
|---|---|---|---|---|---|---|
| 1 | 10.414 | BV | 0.8656 | 7423.72754 | 122.27828 | 47.4450 |
| 2 | 13.358 | VV | 1.1541 | 8223.28613 | 102.21088 | 52.5550 |

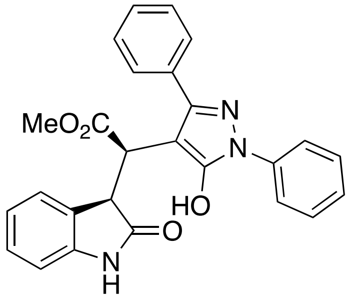


**13o**

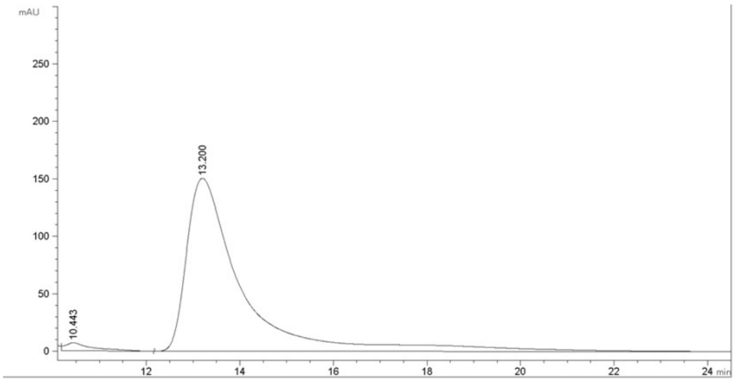


| # | [min] | | [min] | mAU *s | [mAU ] | % |
|---|---|---|---|---|---|---|
| 1 | 10.443 | VB | 0.5463 | 292.71744 | 7.12405 | 2.2981 |
| 2 | 13.200 | BBA | 1.1825 | 1.24448e4 | 150.59853 | 97.7019 |

**(*R*)-4-methyl-3-((*R*)-2,2,2-trifluoro-1-(5-hydroxy-1,3-diphenyl-1*H*-pyrazol-4-yl)ethyl)indolin-2-one (13p)**

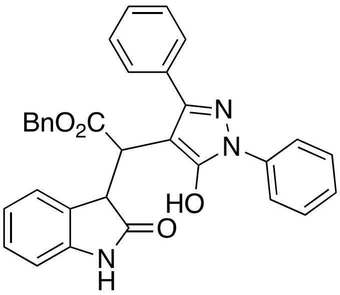


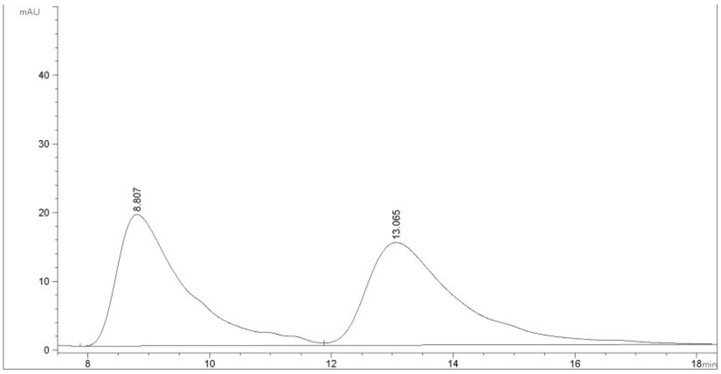


| # | [min] | | [min] | mAU *s | [mAU ] | % |
|---|---|---|---|---|---|---|
| 1 | 8.807 | BV | 1.1321 | 1488.20984 | 19.12426 | 49.1189 |
| 2 | 13.065 | VBA | 1.5108 | 1541.59973 | 14.92546 | 50.8811 |

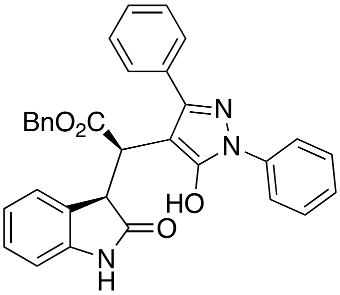


**13p**

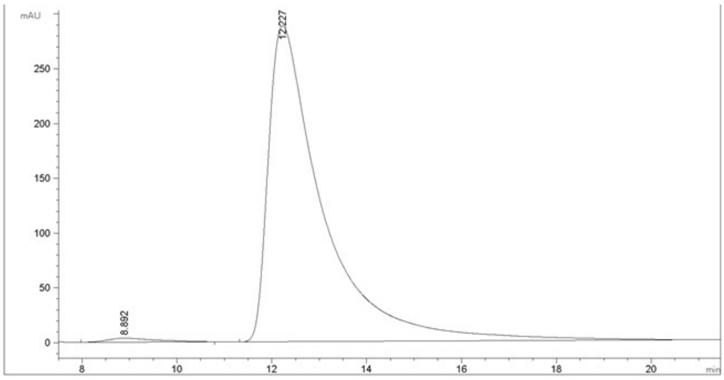


| # | [min] | | [min] | mAU *s | [mAU ] | % |
|---|---|---|---|---|---|---|
| 1 | 8.892 | BV | 1.0126 | 258.28604 | 3.55922 | 1.1024 |
| 2 | 12.227 | BBA | 1.1355 | 2.31704e4 | 288.34030 | 98.8976 |

# 4 X-ray data for compound 13e

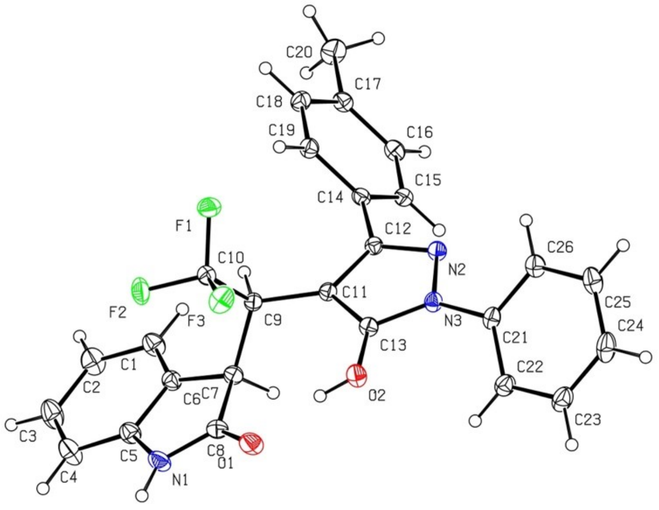


# Data block: mo_220425_hz_1_0m

| | | | |
|---|---|---|---|
| Bond precision: | C-C = 0.0031 A | | Wavelength=1.54178 |
| Cell: | a=11.5944(5) | b=7.9944(4) | c=11.8474(5) |
| | alpha=90 | beta=101.879(2) | gamma=90 |
| Temperature: | 170 K | | |

| | Calculated | Reported |
|---|---|---|
| Volume Space | 1074.62(8) | 1074.62(8) |
| group Hall | P 21 | P 1 21 1 |
| group | P 2yb | P 2yb |
| Moiety formula | C26 H20 F3 N3 O2 | C26 H20 F3 N3 O2 |
| Sum formula | C26 H20 F3 N3 O2 | C26 H20 F3 N3 O2 |
| Mr | 463.45 | 463.45 |
| Dx,g cm-3 | 1.432 | 1.432 |
| Z | 2 | 2 |
| Mu (mm-1) | 0.925 | 0.925 |
| F000 | 480.0 | 480.0 |
| F000’ | 481.68 | |
| h,k,lmax | 13,9,14 | 13,9,14 |
| Nref | 3940[ 2122] | 3929 |
| Tmin,Tmax | 0.957,0.973 | 0.661,0.753 |
| Tmin’ | 0.744 | |

Correction method= # Reported T Limits: Tmin=0.661 Tmax=0.753
AbsCorr = MULTI-SCAN

Data completeness= 1.85/1.00 Theta(max)= 68.525

R(reflections)= 0.0307( 3728) wR2(reflections)= 0.0734( 3929)

S = 1.067 Npar= 309
